# A Characterization of Scale Invariant Responses in Enzymatic Networks

**DOI:** 10.1371/journal.pcbi.1002748

**Published:** 2012-11-01

**Authors:** Maja Skataric, Eduardo D. Sontag

**Affiliations:** 1 Department of Electrical Engineering, Rutgers University, Piscataway, New Jersey, United States of America; 2 Department of Mathematics, Rutgers University, Piscataway, New Jersey, United States of America; University of Notre Dame, United States of America

## Abstract

An ubiquitous property of biological sensory systems is adaptation: a step increase in stimulus triggers an initial change in a biochemical or physiological response, followed by a more gradual relaxation toward a basal, pre-stimulus level. Adaptation helps maintain essential variables within acceptable bounds and allows organisms to readjust themselves to an optimum and non-saturating sensitivity range when faced with a prolonged change in their environment. Recently, it was shown theoretically and experimentally that many adapting systems, both at the organism and single-cell level, enjoy a remarkable additional feature: scale invariance, meaning that the initial, transient behavior remains (approximately) the same even when the background signal level is scaled. In this work, we set out to investigate under what conditions a broadly used model of biochemical enzymatic networks will exhibit scale-invariant behavior. An exhaustive computational study led us to discover a new property of surprising simplicity and generality, uniform linearizations with fast output (ULFO), whose validity we show is both *necessary and sufficient* for scale invariance of three-node enzymatic networks (and sufficient for any number of nodes). Based on this study, we go on to develop a mathematical explanation of how ULFO results in scale invariance. Our work provides a surprisingly consistent, simple, and general framework for understanding this phenomenon, and results in concrete experimental predictions.

## Author Summary

Sensory systems often adapt, meaning that certain measured variables return to their basal levels after a transient response to a stimulus. An additional property that many adapting systems enjoy is that of scale invariance: the transient response remains the same when a stimulus is scaled. This work presents a mathematical study of biochemical enzymatic networks that exhibit scale-invariant behavior.

## Introduction

The survival of organisms depends critically upon their capacity to formulate appropriate responses to sensed chemical and physical environmental cues. These responses manifest themselves at multiple levels, from human sight, hearing, taste, touch, and smell, to individual cells in which signal transduction and gene regulatory networks mediate the processing of measured external chemical concentrations and physical conditions, such as ligand concentrations or stresses, eventually leading to regulatory changes in metabolism and gene expression.

An ubiquitous property of biological sensory systems at all levels is that of *adaptation*: a step increase in stimulus triggers an initial, and often rapid, change in a biochemical or physiological response, followed by a more gradual relaxation toward a basal, pre-stimulus level [1]. Adaptation plays a role in ensuring that essential variables stay within acceptable bounds, and it also allows organisms to readjust themselves to an optimum and non-saturating sensitivity range even when faced with a prolonged change in their operating environment, thus making them capable of detecting changes in signals while ignoring background information.

 Physiological examples of adaptation in higher organisms include phenomena such as the control of the amount of light entering eyes through the contraction and relaxation of the pupil by the nervous system, which brings intensities of illumination within the retinal working range, or the regulation of key metabolites in the face of environmental variations [2]. At the single-cell level, one of the best understood examples of adaptation is exhibited by the *E. coli* chemotaxis sensory system, which responds to gradients of nutrient and ignores constant (and thus uninformative) concentrations [3], [4]. The term “exact” or “perfect” adaptation is employed to describe processes which, after a transient, return with very high accuracy to the same input-independent level. In practice, however, an approximate adaptation property is usually adequate for proper physiological response [5].

By definition, neither the concepts of perfect nor approximate adaptation address the characteristics of the transient signaling which occurs prior to a return to steady state. The amplitude and other characteristics of transient behaviors, however, are physiologically relevant. In this more general context, a remarkable phenomenon exhibited by several human and animal sensory systems is *scale invariance* or logarithmic sensing [2], [6], [7]. This means that responses are functions of ratios (in contrast to actual magnitudes), of a stimulus relative to the background. There is evidence for this phenomenon at an intracellular level as well. It appears in bacterial chemotaxis [8], [9], in the sensitivity of *S. cerevisiae* to fractional rather than absolute pheromone gradients [10], and in two mammalian signaling systems: transcriptional as well as embryonic phenotype responses to 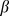-catenin levels in Wnt signaling pathways [11], and nuclear ERK localization in response to EGF signaling [12]. Scale invariance allows systems to react to inputs ranging over several orders of magnitude, and is speculated to help make behaviors robust to external noise as well as to stochastic variations in total expressed concentrations of signaling proteins [13].

Mathematically, scale invariance is defined by the following property of transient behaviors [13]: if a stimulus changes from a background level 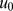 to a new level 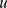, then the entire time response of the system is the same as if the stimulus had changed, instead, from a background level 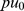 to 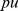. In other words, only the ratio (or “fold-change”) 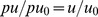 is relevant to the response; the “scale” 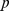 is irrelevant. For this reason, the term “fold change detection” is interchangeably used instead of scale-invariance. Scale invariance implies adaptation, but not every adaptive system is scale invariant [13]. A mathematical analysis of scale-invariance was initiated in [13], [14]. Predictions regarding scale-invariance of *E. coli* chemotaxis were subsequently experimentally verified [15]. While adaptation can be often understood in terms of control-theoretic tools based on linearizations [16], [17], [18], [19], [20], scale invariance is a genuinely **nonlinear** property; as a matter of fact, a linear system can never display scale-invariance, since the response to an input scaled by 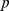 will also be scaled by this same factor 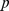.

In this work, we focus on enzymatic signal transduction systems, which involve the activation/deactivation cycles that typically mediate transmission of external signals to transcription factors and other effectors. Networks involving such enzymatic cycles are involved in signal transduction networks from bacterial two-component systems and phosphorelays [21], [22] to actin treadmilling [23], guanosine triphosphatase cycles [24], glucose mobilization [25], metabolic control [26], cell division and apoptosis [27], cell-cycle checkpoint control [28], and the eukaryotic Mitogen-Activated Protein Kinase (MAPK) cascades which mediate growth factor inputs and determine proliferation, differentiation, and apoptosis [29], [30], [31], [32], [33].

Given the biological importance of these processes, and the already observed scale-invariance in some of these pathways [11], [12], we pose here the following question: which enzymatic networks do not merely adapt, but also display scale invariance? In order to answer this question, we performed an exhaustive computational study of all 3-node networks, finely sampled in parameter space. Only about 0.01% of these networks are capable of (approximate) adaptation. Testing which of these adapting networks also display scale-invariant behavior, we found that only about 0.15% of them did. Once that this small subclass was identified, we turned to the problem of determining what network characteristics would explain the results of these numerical experiments. We discovered a surprisingly simple and general property, which we call uniform linearizations with fast output (ULFO), that is displayed by all the networks in this subclass, and here we provide a theoretical framework that explains conceptually why this property is both *necessary and sufficient* for scale invariance of such three-node enzymatic networks. The condition is also sufficient for networks with larger numbers of nodes. As an application (with more than three nodes), we consider a recently published model [34] of an eukaryotic enzymatic system, specifically the pathway involved in the social amoeba *Dictyostelium discoideum*'s chemotactic response to cAMP, and show that our conditions are satisfied in appropriate ranges of cAMP input.

Characterizations of this sort allow one to understand which networks are robust to scale uncertainty, and constitute a powerful tool in allowing one to discard putative mechanisms that are not consistent with experimentally observed scale-invariant behaviors [14], [15].

## Results

### Three-node enzymatic networks

We consider networks consisting of three types of enzymes, denoted respectively as 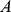, 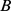, and 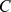. Each of these enzymes can be in one of two states, active or inactive. The fractional concentration of active enzyme 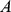 is represented by a variable 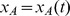, so 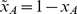 is the fraction of inactive enzyme 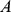. Similar notations are used for 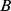 and 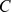. Only enzyme 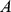 is directly activated by an external input signal, and the response of the network is reported by the fraction of active 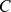. Enzyme 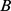 acts as an auxiliary element. Each enzyme may potentially act upon each other through activation (positive regulation), deactivation (negative regulation), or not at all. If a given enzyme is not deactivated by any of the remaining two, we assume that it is constitutively deactivated by a specific enzyme; similarly, if a given enzyme is not activated by any other, there is a constitutively activating enzyme for it. One represents networks by 3-node directed graphs, with nodes labeled 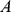, 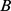, 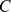, and with edges between two nodes labeled 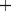 and 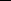 (or “
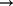
” and “
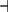
”) to denote positive or negative regulation respectively; no edge is drawn if there is no action. There are 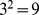 potential directed edges among the three nodes (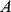 to 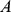, 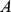 to 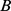, etc.), each of whose labels may be 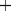, 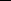, or “none” if there is no edge. This gives a total of 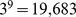 possible graphs. One calls each of these possible graphs a *topology*. Discarding the 3,645 topologies that have no direct or indirect links from the input to the output, there remain 16,038 topologies.

The restriction to three-node networks is made for both practical and biological reasons. As argued in several papers that use a similar approach [20], [35], [36], even though adaptation (as well as scale-invariant) behaviors can, and do, arise in larger networks, the coarse-graining involved in restricting the computational search to minimal networks leads to a tractable search problem, and allows also one to intuitively understand the basic principles. The same motifs are observed in larger networks, in which several nodes may represent a single node in the three-node networks that we study. In fact, the necessary property that we discover for three-node networks turns out to be sufficient, as well, for networks with arbitrary numbers of nodes. The discussion section elaborates further on this point, and an illustration of this reduction is given by an example discussed below of a 6-variable model published in [34] to represent the adaptation kinetics of a chemotaxis signaling pathway in *Dictyostelium discoideum*.

### Specification of a dynamic model

**Figure 1:**
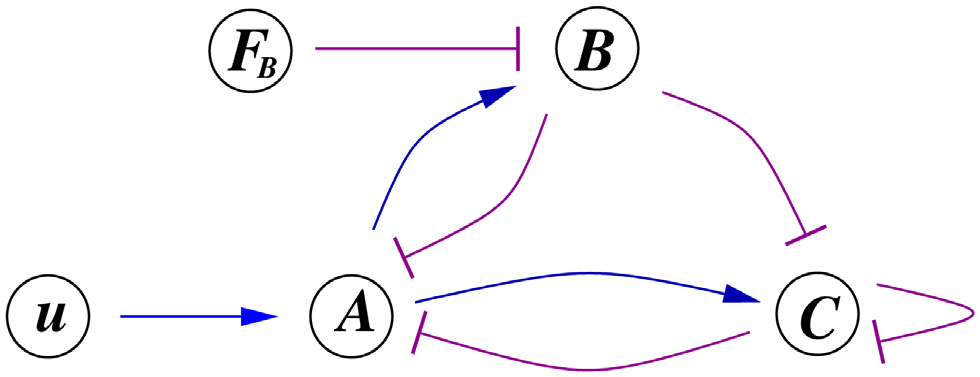
Topology 2293. An example of a topology.

We quantify the effects of each existing regulatory interaction by a Michaelis-Menten term and write a three-variable ordinary differential equation (ODE) that describes the time evolution of 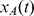, 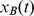, and 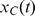:
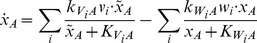
(1a)

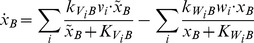
(1b)

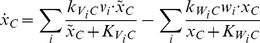
(1c)The 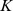
's denote Michaelis-Menten, and the 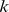
's catalytic, rate constants associated to each regulatory interaction. All the summations range over 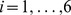. Each “
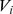
” represents one of 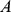, 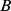, 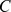, 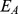, 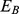, 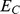, the activating enzymes in the respective equations, and each “
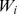
” one of 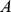, 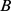, 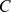, 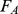, 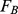, 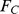, the deactivating enzymes; 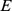 and 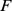 are the constitutively activating and deactivation enzymes, buffered at constant concentrations. (Lower-case variables 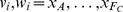 denote active fractions) As an exception, the equation for node 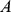 does not include an 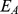 term, but instead includes a term 
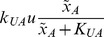
 that models activation of 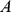 by an external input whose strength at time 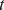 is given by 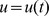 and whose values 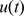 stay within a range 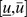. No enzyme appears both an activator and as a deactivator of any given component, that is, 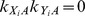, 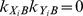, and 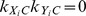, and constitutive enzymes are included only if the reaction would be otherwise irreversible. For example, the topology shown in Fig. 1 is described by the following set of ODE's:
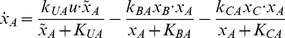
(2a)

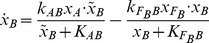
(2b)

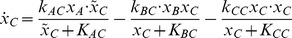
(2c)The term *circuit* is used to refer to a given topology together with a particular choice of the 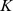 and 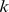 parameters. The three-node model in Eq.1 was employed by Ma et al. [20], in order to classify the minimal enzymatic circuits that adapt. (With the model in [20] that we adopted, there is no direct connection from the input to the output node, and two-node networks are not sufficient for adaptation, while larger adapting networks contain these three-node networks [20]. If one allows direct connections from input to outputs, then two-node networks are able to display adaptation.) The same paradigm has since been used to investigate other network characteristics as well [35], [36].

### Adaptation

Following [37], we define adaptation behavior in terms of two functional metrics. The first metric quantifies the following effect: if we start at steady state, and then step the input at time 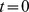 from a value 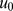 to a different constant value 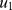, then the system's output, as reported by a response variable 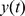 (where 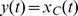 in Eq.1), should return asymptotically to a value that is close to the original value 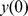. The relative difference in initial and final response 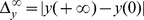 provides a measure of adaptation precision. We say that a system is (approximately) adaptive provided that, for all inputs in the valid range, 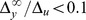, where 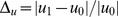 is the relative change in input. In particular, exact or perfect adaptation means that 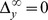. The 10% error tolerance is natural in applications, and the qualitative conclusions are not changed by picking a smaller cutoff [20]. A second metric relies upon the maximal transient difference in output, normalized by the steady-state output, 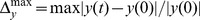. A *signal-detection* property for adaptation [18], [38], should be imposed in order to rule out the trivial situation 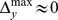 in which a system's output is independent of the input. To avoid having to pick an arbitrary threshold, in this study we follow the convention in [20] of requiring the *sensitivity* 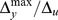 to be greater than one.

### Scale invariance

Scale invariance is the property that if a system starts from a steady state that was pre-adapted (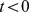) to a certain background level 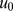, and the input is subsequently set to a new level 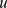 at 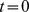, then the entire time response of the system 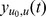 is the same as the response 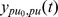 that would result if the stimulus had changed, instead, from 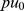 to 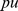. This property should hold for scale changes 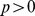 that respect the bounds 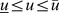 on inputs. For example, recent microfluidics and FRET experimental work [15] verified scale-invariance predictions that had been made in [13] for bacterial chemotaxis under the nonmetabolizable attractant 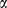-methylaspartate (MeAsp) as an input. In these experiments, *E. coli* bacteria were pre-adapted to input concentrations and then tested in new nutrient gradients, and it was found experimentally that there were two different ranges of inputs 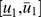 and 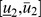 in which scale-invariance holds, the “FCD1” and “FCD2” regimes, repectively. (The term fold-change detection, or FCD, is used to reflect the fact that only the ratio or fold-change 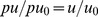 can be detected by the response 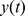.) More generally, the mathematical definition of (perfect) scale invariance [14] imposes the ideal requirement that the same response invariance property is exhibited if 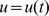, 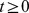 is any time-varying input. The experiments in [15] included excitation by certain oscillatory inputs, for example. In practice, however, this property will always break down for high-frequency inputs, since there are limits to the speed of response of biological systems.

### Adaptive systems need not be scale-invariant

As an illustration of a (perfectly) adaptive yet not scale-invariant system, consider the following equations:
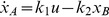
(3a)

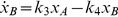
(3b)

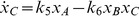
(3c)which is a limiting case of the system described by Eq.2 when 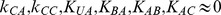, 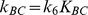, 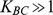 (so 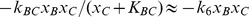), and 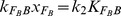 and 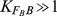. This network perfectly adapts, since at steady state the output is 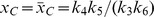, no matter what is the magnitude of the constant input 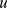, and in fact the system returns to steady state after a step change in input 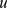, with 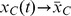 as 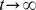 (general stability properties of feedforward circuits shown in [39]). On the other hand, the example in Eq.3 does not display scale invariance. Indeed, consider the solution from an initial state pre-adapted to an input level 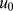, that is 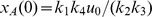, 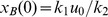, and 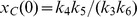, and the input 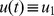 for 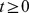. Then, 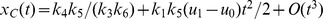 for small 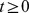. Since the 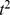 coefficient in this Taylor expansion gets multiplied by 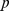 when 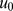 is replaced by 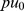 and 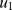 is replaced by 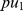, it follows that the transient behavior of the output 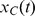 depends on 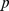. Interestingly, if the equation for the third node is replaced by 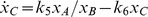, that is to say the activation of 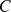 is repressed by 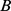, instead of its de-activation being enhanced by 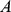, then scale invariance does hold true, because 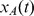 and 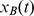 both scale by 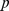 when 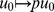, 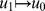, and 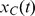 depends on the ratio of these two functions (in particular, the 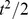 term is 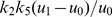). Such a repression is typical of genetic interaction networks, but is not natural in enzymatic reactions.

**Figure 2:**
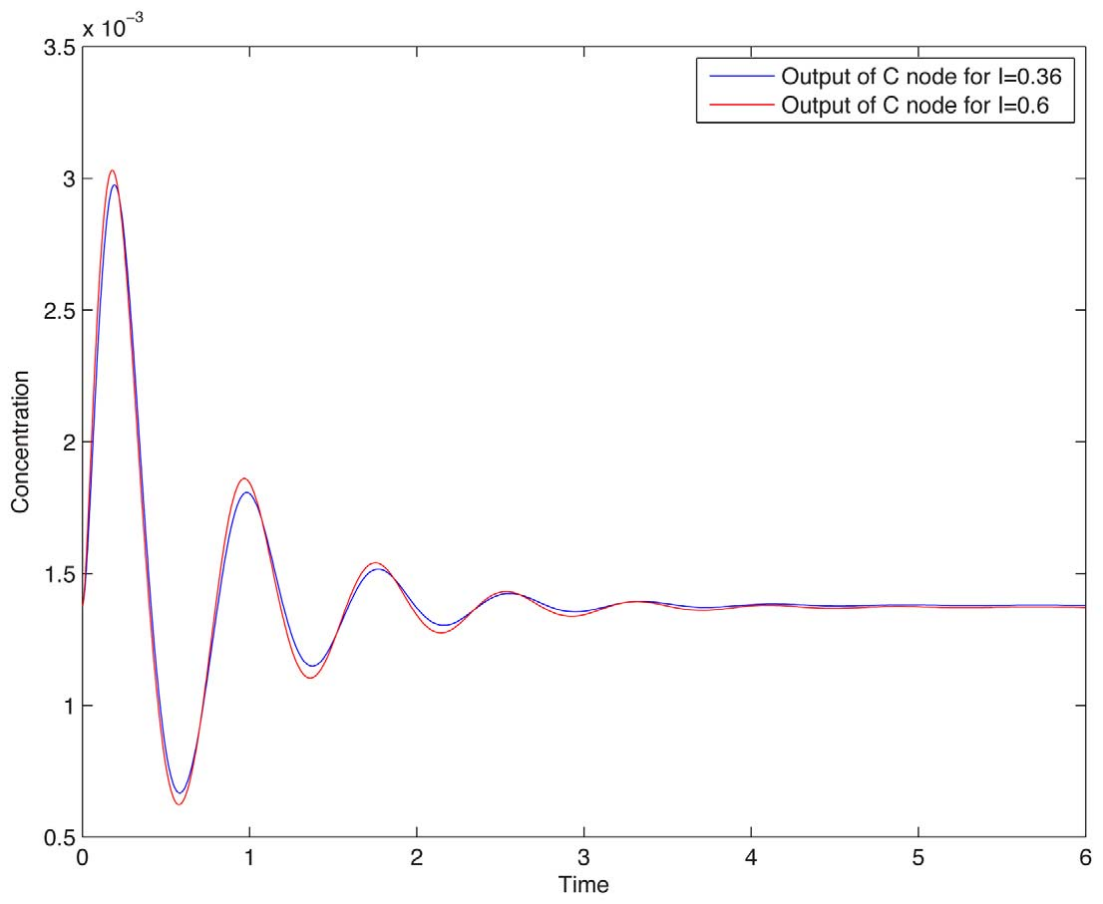
Scale-invariance. Plots overlap, for responses to steps 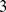 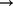 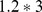 and 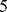 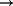 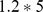. Network is the one described by Eq.2. Random parameter set: 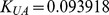 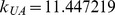, 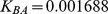 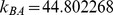, 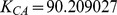 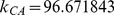, 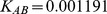 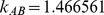, 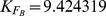 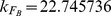, 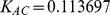 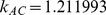, 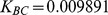 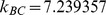, 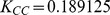 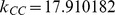.

It turns out that the example described by Eq.3 is typical: no enzymatic network described by Eq.1 can display perfect scale-invariant behavior. This fact is a consequence of the equivariance theorem proved in [14] (see *Materials and Methods*). Thus, a meaningful study of enzymatic networks, even for perfectly adaptive ones, must rely upon a test of approximate scale invariance. Instead of asking that 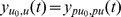, as was the case in the theory developed in [13], [14], one should require only that the difference be small. To investigate this issue, we computationally screened all 3-node topologies through a high-throughput random parameter scan, testing for small differences in responses to scaled steps. We found that approximately 0.01% of the samples showed adaptation, but of them, only about 0.15% passed the additional criterion of approximate scale invariance (see *Materials and Methods*). These samples belonged to 21 (out of 16,038 possible) topologies. As an example of the behavior of one of these, Fig. 2 shows a response resulting from a 20% step, from 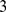 to 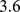, compared to the response obtained when stepping from 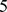 to 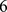; the graphs are almost indistinguishable. (See *Text S1* for an enumeration of circuits and corresponding plots). In the following discussion, we will refer to these surviving circuits, and their topologies, as being “approximately scale invariant” (ASI).

We found that all ASI networks possess a feedforward motif, meaning that there are connections (positively or negatively signed) 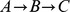 and as well as 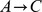. Such feedforward motifs have been the subject of extensive analysis in the systems biology literature [1] and are often involved in detecting changes in signals [40]. They appear in pathways as varied as *E. coli* carbohydrate uptake via the carbohydrate phosphotransferase system [41], control mechanisms in mammalian cells [42], nitric oxide to NF-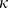
B activation [43], [44], EGF to ERK activation [45], [46], glucose to insulin release [47], [48], ATP to intracellular calcium release [49], and microRNA regulation [50]. The feedforward motifs in all ASI networks are incoherent, meaning such that the direct effect 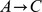 has an opposite sign to the net indirect effect through 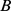. An example of an incoherent feedforward connection is provided by the simple system described by Eq.3 , where the direct effect of 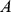 on 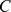 is positive, but the indirect effect is negative: 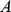 activates 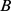 which in turn deactivates 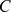. (Not every incoherent feedforward network provides scale invariance; a classification of those that provide exact scale invariance is known [14].)

It is noteworthy that all ASI circuits have a positive regulation from 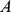 to 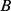 and a negative regulation from 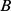 to 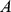. Thus, they all include a negative feedback loop which is nested inside the incoherent feedforward loop. In addition, as discussed below, all ASI circuits and have only a weak (or no) self-loop on the response node 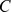.

We then discovered another surprising common feature among all ASI circuits. This feature can best be explained by a further examination of the example in Eq.3 .

### Approximate scale invariance

Continuing with example in Eq.3 , let us suppose that 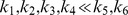, so that the output variable 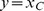 reaches its steady state much faster than 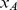 and 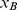 do. Then, we may approximate the original system by the planar linear system represented by the differential equations for 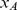 and 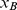 together with the new output variable 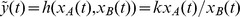, where 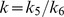. This reduced planar system, obtained by a quasi-steady state approximation, has a perfect scale-invariance property: replacing the input 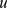 by 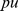 results in the solution 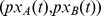, and thus the output is the same: 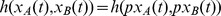. The exact invariance of the reduced system translates into an approximate scale invariance property for the original three-dimensional system because, except for a short boundary-layer behavior (the relatively short time for 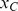 to reach equilibrium), the outputs of both systems are essentially the same, 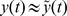. The assumption 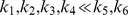 is often written symbolically as 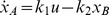, 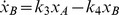, 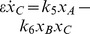, where 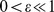 and where 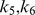 are now the original 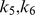 multiplied by 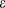. The quality of approximate scale invariance will depend on how small “
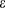
” is.

### Generality of the planar reduction

**Figure 3:**
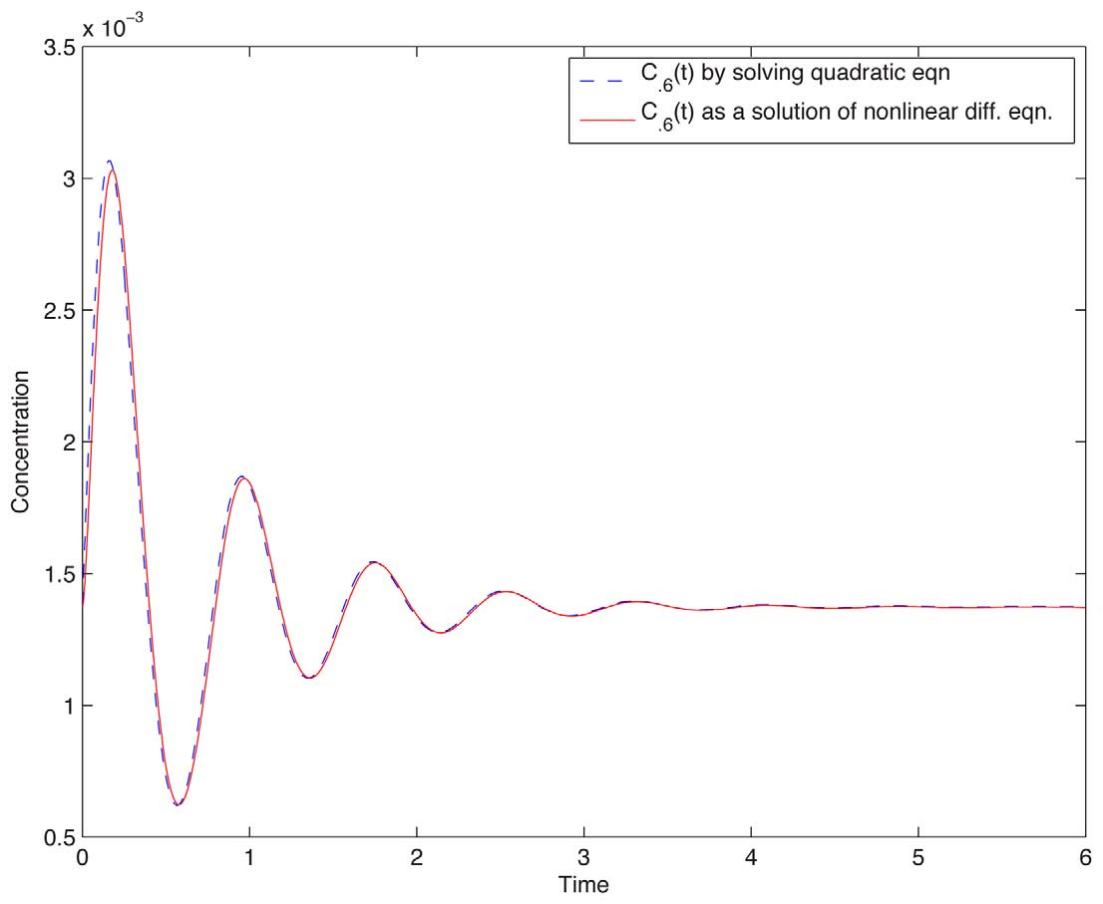
QSS quadratic approximation. Network is the one described by Eq.2. Random parameter set is as in Fig. 2.

 We found that, just as in the example in Eq.3 when 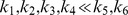, in every ASI circuits the time scale of node 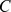 is much shorter than that of 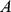 and 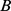. Therefore, the same two-dimensional reduction is always valid. It follows that one can drop the last equation, approximating these circuits by planar systems that are described by only the two state variables 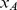 and 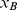, where every occurence of 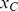 in the first two equations of the right-hand side of Eq.1 is replaced by 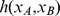, the function obtained by setting the right-hand side of the third equation in Eq.1 to zero and solving for the unique root in the interval 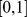 of the quadratic equation. This reduced system, with 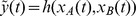 as an output, provides an excellent approximation of the original dynamics. Fig. 3 compares the true response with the response obtained by the quasi-steady state approximation, for one ASI circuit (see *Text S1* for all comparisons).

### Generality of dependence on 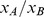


**Figure 4:**
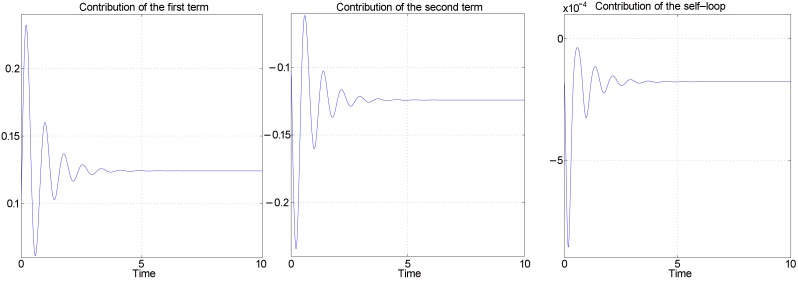
Relative contribution of terms in the equation for node C. The first two terms range in 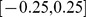 but self-loop magnitude is always less than 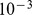. i.e. contribution or self-loop to 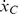 is less than 1%. Similar results hold for all ASI circuits. Network is the one described by Eq.2. Random parameter set is as in Fig. 2 . Similar results are available for all ASI circuits.

In the example given by Eq.3 , there were two additional key mathematical properties that made the planar reduction scale-invariant (and hence the original system approximately so). The first property was that, at equilibrium, the variable 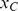 must be a function of the ratio 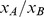, and the second one was that each of 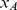 and 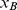 must scale by the same factor when the input scales by 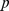. Neither of these two properties need to hold, even approximately, for general networks. Surprisingly, however, we discovered that both are valid with very high accuracy for every ASI circuit. The equilibrium value of 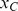 is obtained from setting the last right-hand side of Eq.1 to zero and solving for 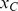. A solution 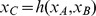 in the interval 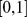 always exists, because at 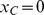 one has 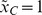 and thus the term is positive, and at 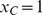 one has 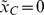 and so the term is negative. This right-hand side has the general form 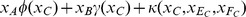, where 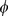 and 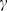 are increasing functions, each a constant multiple of a function of the form 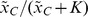 or 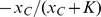. If the term 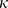 is negligible, then 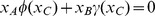 means that also 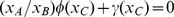, and therefore 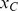 at equilibrium is a (generally nonlinear) function of the ratio 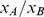. There is no a priori reason for the term 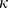 to be negligible. However, we discovered that in every ASI circuit, 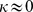. More precisely, there is no dependence on the constitutive enzymes, and this “self-loop” link, when it exists, contributes to the derivative 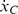 much less than the 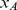 and 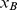 terms, see Fig. 4.

### Generality of homogeneity of 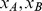


**Figure 5:**
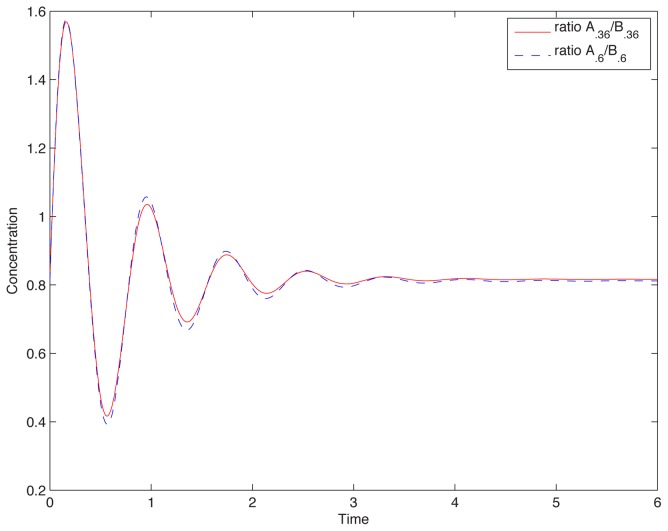
Constant A/B ratio in responses to 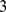 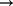 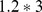 **and** 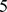 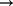 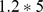. Network is the one described by Eq.2. Random parameter set is as in Fig. 2. Similar results are available for all ASI circuits (see *Text S1*).

The last ingredient of the example given by Eq.3 that plays a role in approximate scale invariance is that each of 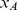 and 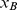 must scale proportionately when the input is scaled. In that example, the property holds simply because the equations for these two variables are linear. In general, however, the dynamics of 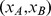 are described by nonlinear equations. Thus it is remarkable that, in all ASI circuits, the property holds. We tested the property by plotting 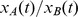 in a set of experiments in which a system was pre-adapted to an input value 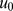 and the input was subsequently set to a new level 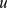 at 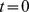. When going from 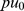 to 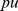, we found that the new value 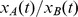 was almost the same, meaning that 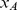 and 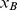 scaled in the same fashion. A representative plot is shown in Fig. 5.

### A new property: uniform linearizations with fast output

The (approximate) independence of 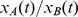 on input scalings is not due to linearity of the differential equations for 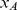 and 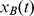. Instead, the analysis of this question led us to postulate a new property, which we call *uniform linearizations with fast output (ULFO)*. To define this property, we again drop the last equation, and approximate circuits by the planar system that has only the state variables 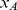 and 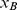, where every occurence of 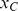 in their differential equations shown in Eq.1 is replaced by 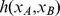. We denote by 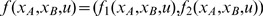 the result of these substitutions, so that the reduced system is described in vector form by 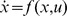, 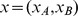. We denote by 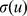 the unique steady state corresponding to a constant input 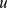, that is, the solution of the algebraic equation 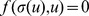. We denote by 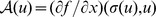 the Jacobian matrix of 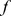 with respect to 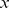, and by 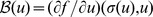 the Jacobian vector of 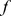 with respect to 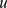.

The property ULFO is then defined by requiring the following properties:

1. time-scale separation for 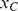;

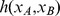
 depends only on the ratio 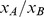;
3. for every 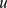 , 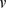, and 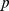 such that 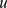, 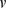, and 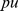 are in the range 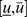:
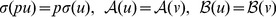
(4)

Notice that we are not imposing the far stronger property that the Jacobian matrices should be constant. We are only requiring the same matrix at every steady state.

The first condition in Eq.4 means that the vector 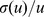 should be constant. We verified that this requirement holds with very high accuracy in every one of the ASI circuits. With 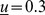 and 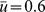, we have the following 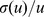 values, rounded to 3 decimal digits: 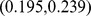, 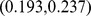, 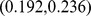, 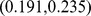 when 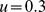, 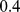, 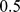, and 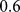 respectively, for the network described by Eq.2 and the random parameter set in Fig. 2 . Similar results are available for all ASI circuits (see *Text S1*).

The Jacobian requirements in Eq.10 are also verified with high accuracy for all the ASI circuits. We illustrate this with the same network and parameter set. Let us we compute the linearizations 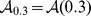, 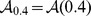, … , 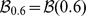 and the average relative differences
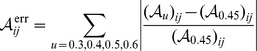
and we define similarly 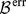. These relative differences are very small (shown to 3 decimal digits):
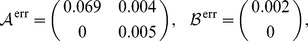
thus justifying the claim that the Jacobians are practically constant. Similar results are available for all ASI circuits (see *Text S1*).

The key theoretical fact is that the property ULFO implies approximate scale-invariance, see *Materials and Methods*.

Intuitively, the conditions in Eq.4 mean that the “memory” of past inputs, represented by the activity level (phosphorylation, methylation, etc.) of the pre-adapted steady state, is proportional to the input, indicating an integration mechanism, and that the small-signal behavior from different pre-adapted levels is the same. The term “uniform” refers to the fact that the linearizations at every steady state are the same. If the linearizations are not all the same, it is easy to see that scale invariance does not hold. The uniformity of linearizations provides a “global” way to tie together behaviors at different scales. The conditions give us the approximate homogeneity property 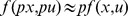 when near steady states, because, for 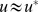 and 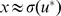:
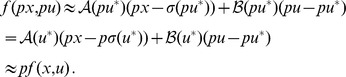



These conditions are satisfied in various combinations of parameter regimes. As a purely theoretical example, consider the following system (denoting 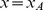, 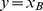, 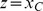):
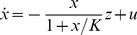


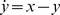


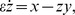
which can be viewed as a limiting case of the system described by Eq.2 when
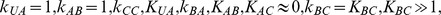


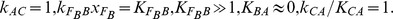



Substituting 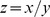 in the first equation, we have:
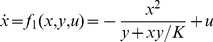
The linearization of the system evaluated at a steady state corresponding to a constant input 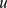 has
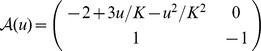
(and 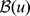 constant), and is therefore approximately constant provided that 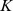 is large or that the input 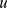 is small in relative magnitude. Similarly, if we use 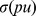 as initial state and 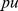 as inputs, we get a similar expression (with 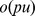 instead of 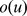 and the 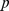
's in the fraction canceling out).

### A concrete biological model

In a recent paper [34] Takeda and collaborators studied the adaptation kinetics of a eukaryotic chemotaxis signaling pathway, employing a microfluidic device to expose *Dictyostelium discoideum* to changes in chemoeffector cyclic adenosine monophosphate (cAMP). Specifically, they focused on the dynamics of activated Ras (Ras-GTP), which was in turn reported by RBD-GFP (the Ras binding domain of fluorescently tagged human Raf1), and showed almost perfect adaptation of previously unstimulated cells to cAMP concentrations ranging from 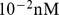 to 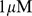. Furthermore, inspired by [20], the authors proposed alternative models for adaptation, and concluded that the best fit was obtained by using an incoherent feedforward structure. The model that they identified is given by the following system of 6 differential equations:
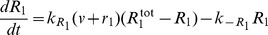


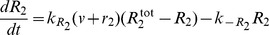


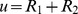


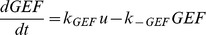


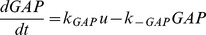


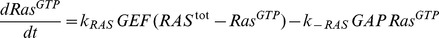


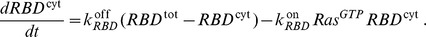



The symbol 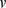 stands for the chemoeffector cAMP, and the authors assumed the existence of two different receptor populations (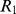 and 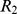, with very different 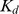
's) which when bound pool their signals to downstream components (through 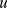). The constants 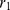 and 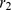 represent levels of constitutive activation. The variables 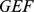 and 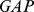 represent activation and deactivation of RasGEF and RasGAP, 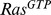 represents the activated Ras, and 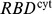 describes the cytosolic reporter molecule RBD-GFP.

**Figure 6:**
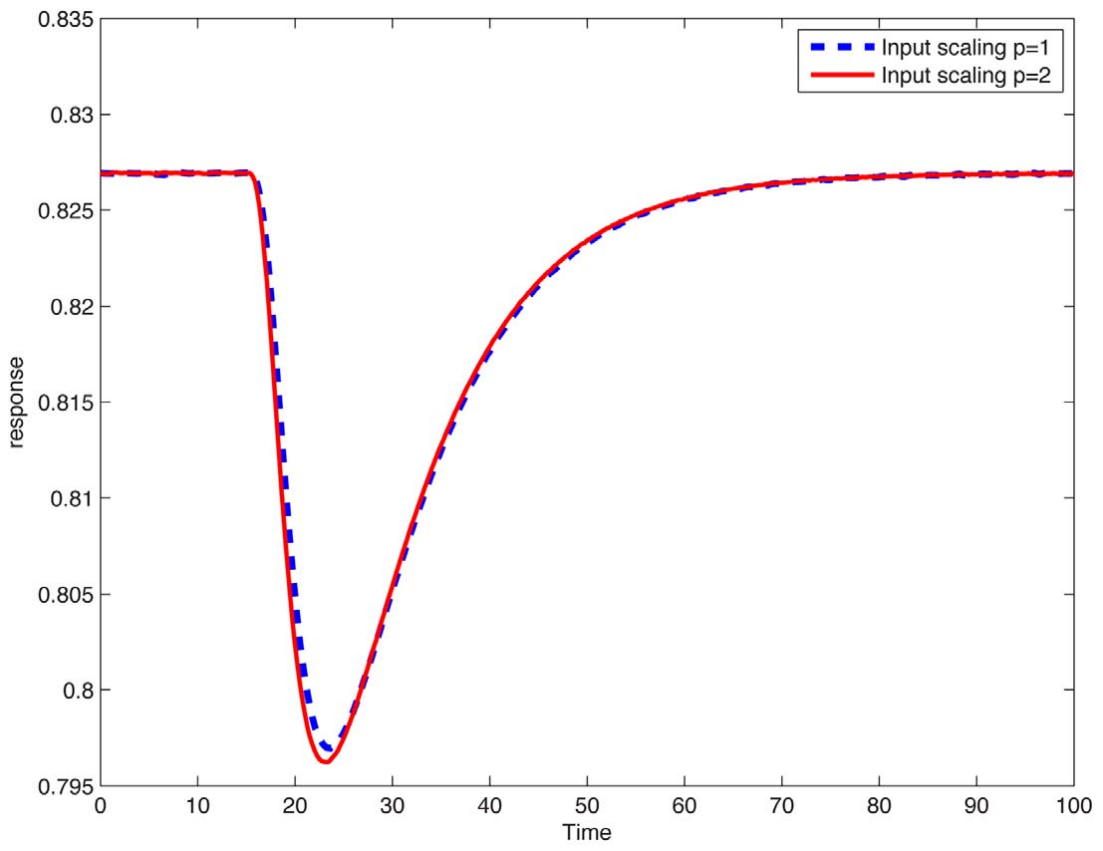
Scale-invariance computed when using the model in [**34**]: Responses to steps 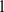 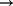 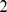 and 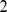 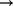 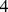 coincide.

The best-fit parameters obtained in [34] are as follows: 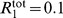, 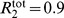, 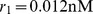, 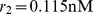, 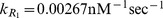, 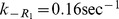, 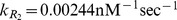, 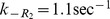, 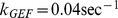, 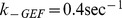, 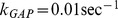, 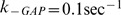, 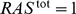, 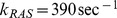, 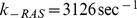, 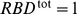, 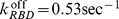, 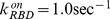. With these parameters, and cAMP concentrations which are small yet also satisfy 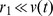 and 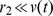, it follows that 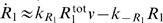 and 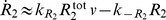, so we may view 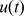 as an input (linearly dependent on the external 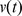) to the three-variable system described by 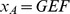, 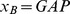, 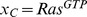. Since 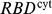 depends only on 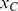, we may view 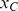 as the output. This three-variable system (interpreted as having limiting values of Michaelis-Menten constants) has the ULFO property provided that the dynamics of 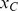 are fast compared to 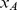 and 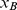, which the identified parameters insure. So, we expect scale-invariant behavior. Indeed, Fig. 6 shows a simulation of the entire six-dimensional system (not merely of our 3-dimensional reduction) when using a step from 1 to 2 nM of cAMP, and shows that essentially the same response is obtained when stepping from 2 to 4 nM. This prediction of scale-invariant behavior is yet to be tested experimentally.

## Discussion

Work in molecular systems biology seeks to unravel the basic dynamic processes, feedback control loops, and signal processing mechanisms in single cells and entire organisms, both for basic scientific understanding and for guiding drug design. One of the key questions is: how can one relate phenotype (function) to interaction maps (gene networks, protein graphs, and so forth) derived from experimentation, especially those obtained from high-throughput tools? Answers to this question provide powerful tools for guiding the reverse-engineering of networks, by focusing on mechanisms that are consistent with experimentally observed behaviors, and, conversely, from a synthesis viewpoint, allow one to design artificial biological systems that are capable of adaptation [51] and other objectives. In particular, scale-invariance, a property that has been observed in various systems [11], [12], can play a key role in this context, helping to discard putative mechanisms that are not consistent with experimentally observed scale-invariant behaviors [15]. Through a computational study, we identified a set of simple mathematical conditions that are used to characterize three-node scale invariant enzymatic networks.

The conditions that we obtained for three-node networks are also sufficient for an arbitrary number of nodes, in the following sense. Suppose that we consider a set of 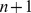 nodes, where 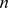 nodes are described by variables 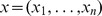 and an additional node is described by a variable 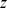. Suppose that the 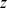 variable evolves at a faster time scale than the 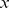 variables. Then, the ULFO property implies approximate scale invariance (see *Materials and Methods*). A variation of this situation is that in which a three-node network already displays scale invariance through an output node 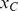, and this output feeds into an additional node 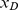 which evolves in a linear mode; then the entire four-node network will display scale invariance as well. Yet another variation is that in which an input is processed linearly before being fed into a three-node network. The discussed example of a published chemotaxis pathway in *Dictyostelium discoideum* combines these variations. One could ask, of course, whether there exist large networks that are scale invariant yet are not built in this fashion. We carried out a limited computational search with four-node networks and have found none so far, leading us to conjecture that the ULFO mechanism is indeed necessary as well as sufficient in larger networks. However, a complete proof of necessity for arbitrary networks is outside the scope of this paper, and is most likely a very difficult if not impossible problem. A full computational screen as performed for three-node networks is already infeasible for four-node networks, due to the combinatorial explosion in the number of possible networks and of parameters to be randomly tested. A theoretical proof is also very difficult to envision, because (a) exact scale invariance is impossible for enzymatic networks, as shown in this paper, and (b) approximate adaptation and scale invariance are mathematically very hard to formalize in such a manner that impossibility can be rigorously proved for systems that do not satisfy our characterizations. In any event, as has been argued in other recent papers dealing with biological adaptation by enzymatic networks [20], [35], [36], a restriction to three-node networks is biologically reasonable, both as a coarse-graining of the problem and because many eukaryotic biological pathways, such as MAPK pathways, have at their core a three-component architecture.

## Materials and Methods

### Computational screen

We generalized and extended the computational protocol developed for adaptation in [20] to an investigation of approximate scale invariance. MATLAB scripts were used, in conjunction with the software developed in [20]. In order to test inputs in ranges of the form 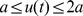, redefining the constant 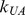 if needed, we take simply 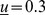 and 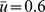. We considered 160,380,000 circuits, obtained from the 16,038 nontrivial 3-node topologies, each one with 10,000 parameters sampled in logarithmic scale using the Latin hypercube method [52]. (We picked the ranges 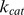 = 0.1–10 and 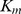 = 0.001–100. A finer sampling does not affect conclusions in any significant way [20].) Of these, 0.01% (16,304) circuits showed adaptation, meaning that, as in [20], when making a 20% step from 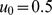 to 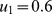 the precision is 10% or better, and the sensitivity is at least unity. Approximate scale invariance (ASI) was then tested by also performing a 20% step experiment from 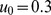 to 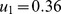 and requiring that the relative difference between the responses be at most 10%: 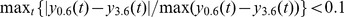


Of the adapting circuits, about 0.15% (25 circuits, classified into 21 different topologies) were determined to be ASI.

### ULFO implies approximate scale invariance, for any number of nodes

Consider a system of 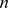 differential equations with input signal 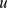,
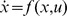
with the variables 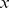 evolving on some closed bounded set and 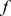 differentiable, and suppose that for each constant input 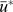 there is a unique steady state 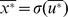 with the conditions in Eq.10 and an output
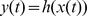
such that 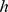 is differentiable and homogeneous of degree zero (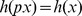 for nonzero 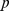). We view 3-node enzymatic networks as obtained from a set of 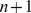 equations
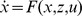


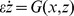
with 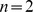, 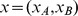, and 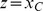 (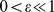 represents the faster time scale for 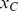), and we are studying the reduced system 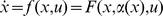 obtained by solving 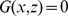 for 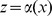 and substituting in 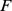. Consider a time interval 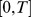, a constant input 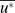, and a possibly time-varying input 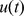, 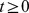, as well as a scaling 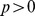, such that all values 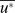, 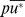, 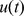, 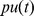 are in the input range 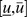. The solutions of 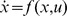 with initial condition 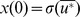 and of 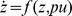 with initial condition 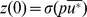 are denoted respectively by 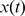 and 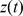, and the respective outputs are 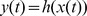 and 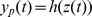. We wish to show that these two responses are approximately equal on 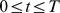.

More precisely, we will prove that the relative error
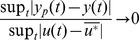
as a function of the input perturbation 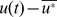.

Write 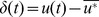. From Theorem 1 in [16] we know that
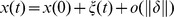
where 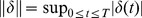 and 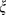 is the solution of the variational system
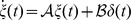
with 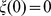, and that
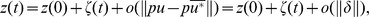
where
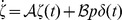
with 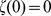. Recall that 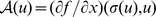 is the Jacobian matrix of 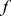 with respect to 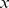, and 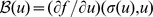 is the Jacobian vector of 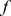 with respect to 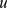, and the assumptions are that these matrices are in fact independent of 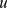. By linearity, 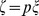. Using 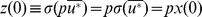, we have that 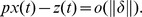 Thus,
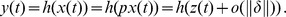



If 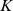 is an upper bound on the gradient of 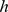, then
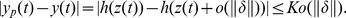



Thus, the relative error 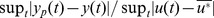 converges to zero as a function of the input perturbation 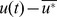, as claimed.

As a numerical illustration, we consider again the the network described by Eq.2 and the random parameter set in Fig. 2 . We compare the relative error between the original nonlinear system, with initial state 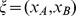 corresponding to 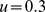, and applied input 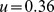, and the approximation is 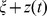, where the 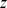 solves the linear system with initial condition zero and constant input 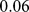. The maximum approximation error is about 5% (to 3 decimal places, 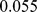 for 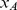 and 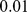 for 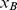). When stepping from 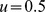 to 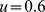, the error is less than 3% (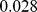 and 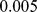 respectively). Similar results are available for all ASI circuits (see *Text S1*).

### Impossibility of perfect scale-invariance

Consider any system with state 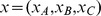, output 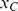, and equations of the general form:
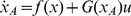


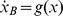


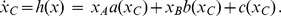



It is assumed that 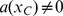 for all 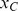, 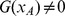 for all 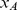, 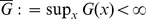, and the system is irreducible [14]. We now prove that such a system cannot be scale-invariant. Suppose by way of contradiction that it would be, and pick any fixed 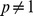. The main theorem in [14] insures that there are two differentiable functions 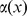 and 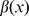 such that the algebraic identities:
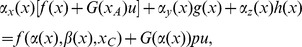


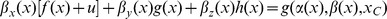


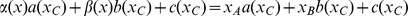
hold for all constant 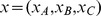 and 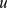, and the vector function 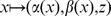 is one-to-one and onto, which implies in particular that
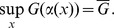



Dividing by 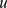 and taking the limit as 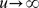 in the first identity, we conclude that 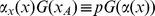. Doing the same in the second identity, we conclude that 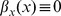. Finally, taking partial derivatives with respect to 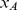 in the third identity:
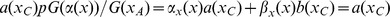
is true for all 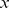. Since *a*(*x_C_*)≢0, it follows that
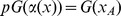
for all 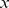. We consider two cases: (a) 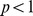 and (b) 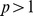. Suppose 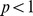. Pick any sequence of points 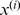 with 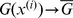 as 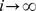. Then 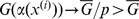, contradicting 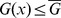. If 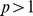, picking a sequence such that 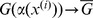 as 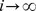 gives the contradiction 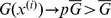. This shows that the FCD property cannot hold.

## Supporting Information

Text S1Supplementary Text describes the dynamics of 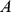 and 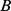 nodes for the linearized models, as well as the ratio between 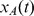 and 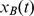.(PDF)

## References

[1] Alon U (2006) An Introduction to Systems Biology: Design Principles of Biological Circuits. Chapman & Hall.

[2] Keener J, Sneyd J (1998) Mathematical Physiology. New York: Springer.

[3] Block SM, Segall JE, Berg HC (1983) Adaptation kinetics in bacterial chemotaxis. J Bacteriol 154: 312–323. 6339475 10.1128/jb.154.1.312-323.1983 PMC217461

[4] Shimizu TS, Tu Y, Berg HC (2010) A modular gradient-sensing network for chemotaxis in Escherichia coli revealed by responses to time-varying stimuli. Mol Syst Biol 6: 382. 20571531 10.1038/msb.2010.37 PMC2913400

[5] Mello BA, Tu Y (2003) Perfect and near-perfect adaptation in a model of bacterial chemotaxis. Biophys J 84: 2943–2956. 12719226 10.1016/S0006-3495(03)70021-6 PMC1302857

[6] Laming D (1986) Sensory Analysis. London: Academic Press.

[7] Thompson R (1967) Foundations of physiological psychology. New York: Harper and Row.

[8] Kalinin YV, Jiang LL, Tu YH, Wu M (2009) Logarithmic sensing in Escherichia coli bacterial chemotaxis. Biophysical Journal 96: 2439–2448. 19289068 10.1016/j.bpj.2008.10.027 PMC2989150

[9] R Mesibov GWO, Adler J (1973) The range of attractant concentrations for bacterial chemotaxis and the threshold and size of response over this range. J Gen Physiol 62: 203–223. 4578974 10.1085/jgp.62.2.203 PMC2226111

[10] Paliwal S, Iglesias PA, Campbell K, Hilioti Z, Groisman A, et al (2007) MAPK-mediated bimodal gene expression and adaptive gradient sensing in yeast. Nature 446: 46–51. 17310144 10.1038/nature05561

[11] Goentoro L, Kirschner MW (2009) Evidence that fold-change, and not absolute level, of *β* -catenin dictates Wnt signaling. Molecular Cell 36: 872–884. 20005849 10.1016/j.molcel.2009.11.017 PMC2921914

[12] Cohen-Saidon C, Cohen AA, Sigal A, Liron Y, Alon U (2009) Dynamics and variability of ERK2 response to EGF in individual living cells. Molecular Cell 36: 885–893. 20005850 10.1016/j.molcel.2009.11.025

[13] Shoval O, Goentoro L, Hart Y, Mayo A, Sontag E, et al (2010) Fold change detection and scalar symmetry of sensory input _elds. Proc Natl Acad Sci U S A 107: 15995–16000. 20729472 10.1073/pnas.1002352107 PMC2936624

[14] Shoval O, Alon U, Sontag E (2011) Symmetry invariance for adapting biological systems. SIAM Journal on Applied Dynamical Systems 10: 857–886.

[15] Lazova MD, Ahmed T, Bellomo D, Stocker R, Shimizu TS (2011) Response-rescaling in bacterial chemotaxis. Proc Natl Acad Sci U S A 108: 13870–13875. 21808031 10.1073/pnas.1108608108 PMC3158140

[16] Sontag E (1998) Mathematical Control Theory. Deterministic Finite-Dimensional Systems, volume 6 of Texts in Applied Mathematics, second edition. New York: Springer-Verlag. xvi+531 pp.

[17] Yi TM, Huang Y, Simon M, Doyle J (2000) Robust perfect adaptation in bacterial chemotaxis through integral feedback control. Proc Natl Acad Sci U S A 97: 4649–4653. 10781070 10.1073/pnas.97.9.4649 PMC18287

[18] Sontag E (2003) Adaptation and regulation with signal detection implies internal model. Systems Control Lett 50: 119–126.

[19] Iglesias P (2003) Feedback control in intracellular signaling pathways: Regulating chemotaxis in dictyostelium discoideum. European J Control 9: 216–225.

[20] Ma W, Trusina A, El-Samad H, Lim WA, Tang C (2009) Defining network topologies that can achieve biochemical adaptation. Cell 138: 760–773. 19703401 10.1016/j.cell.2009.06.013 PMC3068210

[21] Bijlsma J, Groisman E (2003) Making informed decisions: regulatory interactions between two-component systems. Trends Microbiol 11: 359–366. 12915093 10.1016/s0966-842x(03)00176-8

[22] Grossman A (1995) Genetic networks controlling the initiation of sporulation and the development of genetic competence in bacillus subtilis. Annu Rev Genet 29: 477–508. 8825484 10.1146/annurev.ge.29.120195.002401

[23] Chen H, Bernstein B, Bamburg J (2000) Regulating actin filament dynamics in vivo. Trends Biochem Sci 25: 19–23. 10637608 10.1016/s0968-0004(99)01511-x

[24] Donovan S, Shannon K, Bollag G (2002) GTPase activating proteins: critical regulators of intracellular signaling. Biochim Biophys Acta 1602: 23–45. 11960693 10.1016/s0304-419x(01)00041-5

[25] Karp G (2002) Cell and Molecular Biology. Wiley.

[26] Stryer L (1995) Biochemistry. Freeman.

[27] Sulis M, Parsons R (2003) PTEN: from pathology to biology. Trends Cell Biol 13: 478–483. 12946627 10.1016/s0962-8924(03)00175-2

[28] Lew D, Burke D (2003) The spindle assembly and spindle position checkpoints. Annu Rev Genet 37: 251–282. 14616062 10.1146/annurev.genet.37.042203.120656

[29] Asthagiri A, Lauffenburger D (2001) A computational study of feedback effects on signal dynamics in a mitogen-activated protein kinase (mapk) pathway model. Biotechnol Prog 17: 227–239. 11312698 10.1021/bp010009k

[30] Chang L, Karin M (2001) Mammalian MAP kinase signaling cascades. Nature 410: 37–40. 11242034 10.1038/35065000

[31] Huang CY, Jr JF (1996) Ultrasensitivity in the mitogen-activated protein kinase cascade. Proc Natl Acad Sci U S A 93: 10078–10083. 8816754 10.1073/pnas.93.19.10078 PMC38339

[32] Widmann C, Spencer G, Jarpe M, Johnson G (1999) Mitogen-activated protein kinase: Conservation of a three-kinase module from yeast to human. Physiol Rev 79: 143–180. 9922370 10.1152/physrev.1999.79.1.143

[33] Angeli D, Ferrell JE, Sontag E (2004) Detection of multistability, bifurcations, and hysteresis in a large class of biological positive-feedback systems. Proc Natl Acad Sci U S A 101: 1822–1827. 14766974 10.1073/pnas.0308265100 PMC357011

[34] Takeda K, Shao D, Adler M, Charest P, Loomis W, et al (2012) Incoherent feedforward control governs adaptation of activated Ras in a eukaryotic chemotaxis pathway. Sci Signal 5 205: ra2. 22215733 10.1126/scisignal.2002413 PMC3928814

[35] Shah NA, Sarkar CA (2011) Robust network topologies for generating switch-like cellular responses. PLoS Comput Biol 7: e1002085. 21731481 10.1371/journal.pcbi.1002085 PMC3121696

[36] Yao G, Tan C, West M, Nevins JR, You L (2011) Origin of bistability underlying mammalian cell cycle entry. Mol Syst Biol 7: 485. 21525871 10.1038/msb.2011.19 PMC3101952

[37] Francois P, Siggia ED (2008) A case study of evolutionary computation of biochemical adaptation. Phys Biol 5: 026009. 18577806 10.1088/1478-3975/5/2/026009

[38] Andrews B, Sontag E, Iglesias P (2008) An approximate internal model principle: Applications to nonlinear models of biological systems. In: Proceedings of the 17th IFAC World Congress; 6–11 July 2008; Seoul, Korea. pp. Paper FrB25.3, 6 pages.

[39] Sontag E (2010) Remarks on feedforward circuits, adaptation, and pulse memory. IET Systems Biology 4: 39–51. 20001091 10.1049/iet-syb.2008.0171

[40] Mangan S, Itzkovitz S, Zaslaver A, Alon U (2006) The incoherent feed-forward loop accelerates the response-time of the gal system of Escherichia coli. J Mol Biol 356: 1073–1081. 16406067 10.1016/j.jmb.2005.12.003

[41] Kremling A, Bettenbrock K, Gilles ED (2008) A feed-forward loop guarantees robust behavior in escherichia coli carbohydrate uptake. Bioinformatics 24: 704–710. 18187443 10.1093/bioinformatics/btn010

[42] Ma'ayan A, Jenkins SL, Neves S, Hasseldine A, Grace E, et al (2005) Formation of regulatory patterns during signal propagation in a Mammalian cellular network. Science 309: 1078–1083. 16099987 10.1126/science.1108876 PMC3032439

[43] Mahaut-Smith MP, Ennion SJ, Rolf MG, Evans RJ (2000) ADP is not an agonist at P2X(1) receptors: evidence for separate receptors stimulated by ATP and ADP on human platelets. Br J Pharmacol 131: 108–114. 10960076 10.1038/sj.bjp.0703517 PMC1572284

[44] Marsigliante S, Elia MG, Di Jeso B, Greco S, Muscella A, et al (2002) Increase of [Ca(2+)](i) via activation of ATP receptors in PC-Cl3 rat thyroid cell line. Cell Signal 14: 61–67. 11747990 10.1016/s0898-6568(01)00208-x

[45] Sasagawa S, Ozaki Y, Fujita K, Kuroda S (2005) Prediction and validation of the distinct dynamics of transient and sustained ERK activation. Nat Cell Biol 7: 365–373. 15793571 10.1038/ncb1233

[46] Nagashima T, Shimodaira H, Ide K, Nakakuki T, Tani Y, et al (2007) Quantitative transcriptional control of ErbB receptor signaling undergoes graded to biphasic response for cell differentiation. J Biol Chem 282: 4045–4056. 17142811 10.1074/jbc.M608653200

[47] Menè P, Pugliese G, Pricci F, Di Mario U, Cinotti GA, et al (1997) High glucose level inhibits capacitative Ca2+ inux in cultured rat mesangial cells by a protein kinase C-dependent mechanism. Diabetologia 40: 521–527. 9165219 10.1007/s001250050710

[48] Nesher R, Cerasi E (2002) Modeling phasic insulin release: immediate and time-dependent effects of glucose. Diabetes 51 Suppl 1 S53–59. 11815459 10.2337/diabetes.51.2007.s53

[49] Ridnour LA, Windhausen AN, Isenberg JS, Yeung N, Thomas DD, et al (2007) Nitric oxide regulates matrix metalloproteinase-9 activity by guanylyl-cyclase-dependent and -independent pathways. Proc Natl Acad Sci U S A 104: 16898–16903. 17942699 10.1073/pnas.0702761104 PMC2040425

[50] Tsang J, Zhu J, van Oudenaarden A (2007) MicroRNA-mediated feedback and feedfor-ward loops are recurrent network motifs in mammals. Mol Cell 26: 753–767. 17560377 10.1016/j.molcel.2007.05.018 PMC2072999

[51] Bleris L, Xie Z, Glass D, Adadey A, Sontag E, et al (2011) Synthetic incoherent feed-forward circuits show adaptation to the amount of their genetic template. Nature Molecular Systems Biology 7: 519. 10.1038/msb.2011.49 PMC3202791 21811230

[52] Iman RL (2001) Appendix A : Latin Hypercube Sampling 1. Encyclopedia of Statistical Sciences, Update 3: 408–411.

