# Supplementary material for: A Characterization of Scale Invariant Responses in Enzymatic Networks
Source: PLoS Comput Biol. 2012 Nov 1;8(11):e1002748. doi: 10.1371/journal.pcbi.1002748 (PMC3486845; doi:10.1371/journal.pcbi.1002748)
Supplement: Text S1 — Supplementary Text describes the dynamics of and nodes for the linearized models, as well as the ratio between and . (PDF) [file pcbi.1002748.s001.pdf]

Supplementary Material

A characterization of scale invariant responses in enzymatic networks

1 Circuits that exhibit ASI

We list here the results of the computational screen as described in the Main Text. Equations and parameters for the 25 identified ASI circuits (21 topologies) are given. Firstly, we give graphical representation of the 25 circuits.

For each circuit, four plots are shown:

(a) a comparison between the plots of $x_A(t)$ and $x_B(t)$ for the original nonlinear system and the respective plots for the linearized approximations,

(b) the plots showing scale-invariant behavior for step inputs,

and the comparison between the plots of $x_C(t)$ for the original nonlinear system and for the quasi-steady state approximation, for

(c) step input change from 0.3 to 0.36 and

(d) step input change from 0.5 to 0.6.

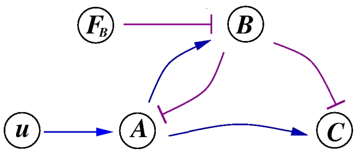

(a) Circuit 1.

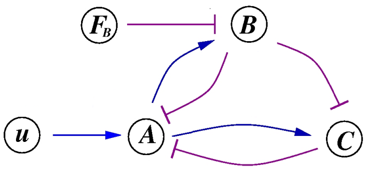

(b) Circuit 2.

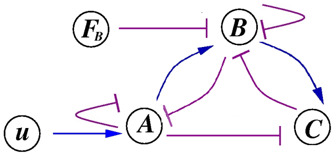

(c) Circuit 3.

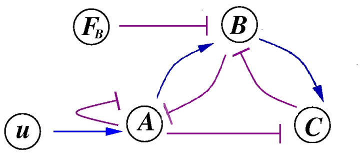

(d) Circuit 4.

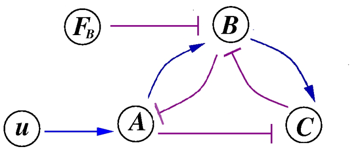

(e) Circuit 5.

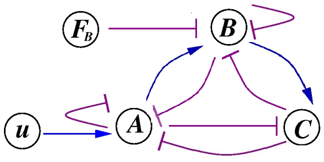

(f) Ciircuit 6.

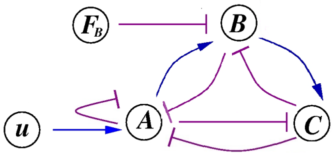

(g) Circuit 7.

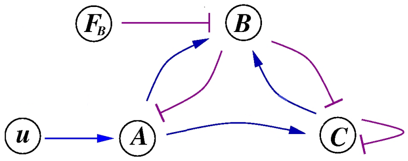

(h) Circuit 8.

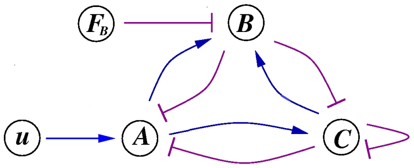

(i) Circuit 9.

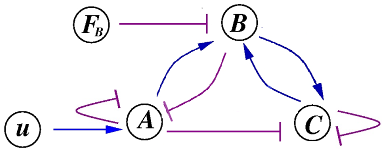

(j) Circuit 10

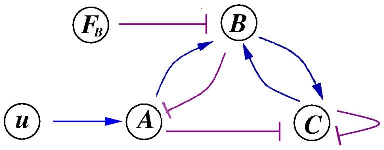

(k) Circuit 11.

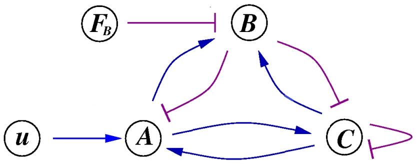

(l) Circuit 12.

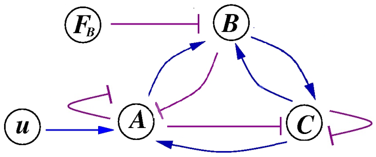

(m) Circuit 13.

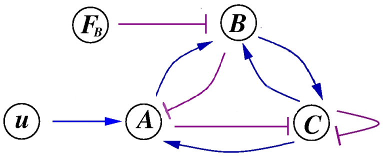

(n) Circuit 14.

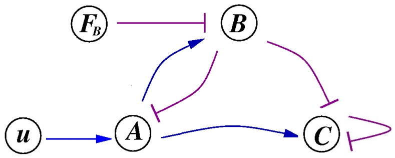

(o) Circuits 15 -17

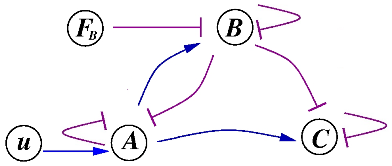

(p) Circuit 18.

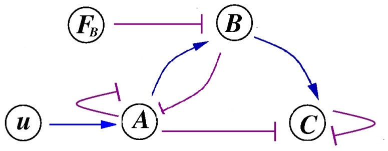

(q) Circuit 19.

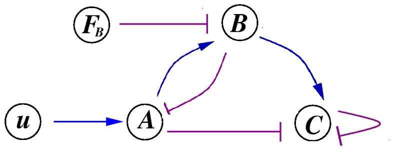

(r) Circuit 20.

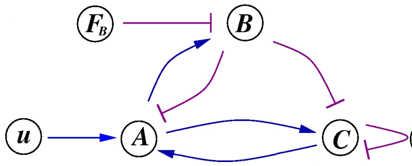

(s) Circuit 21 - 22

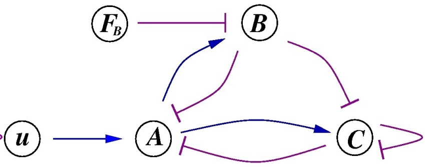

(t) Circuit 23.

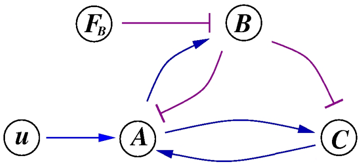

(u) Circuit 24 - 25

Figure S1. Identified ASI Circuits

Circuit 1.

$$
\begin{aligned}
\dot{x}_A &= k_{uA} u \frac{\widetilde{x}_A}{\widetilde{x}_A + K_{uA}} - k_{BA} x_B \frac{x_A}{x_A + K_{BA}} \\
\dot{x}_B &= k_{AB} x_A \frac{\widetilde{x}_B}{\widetilde{x}_B + K_{AB}} - k_{F_B B} x_{F_B} \frac{x_B}{x_B + K_{F_B B}} \\
\dot{x}_C &= k_{AC} x_A \frac{\widetilde{x}_C}{\widetilde{x}_C + K_{AC}} - k_{BC} x_B \frac{x_C}{x_C + K_{BC}}
\end{aligned}
$$

Parameters: $K_{AB} = 0.001191$; $k_{AB} = 1.466561$; $K_{AC} = 0.113697$; $k_{AC} = 1.211993$; $K_{BA} = 0.001688$; $k_{BA} = 44.802268$; $K_{BC} = 0.009891$; $k_{BC} = 7.239357$; $K_{uA} = 0.093918$; $k_{uA} = 11.447219$; $k_{AC} = 1.211993$; $K_{AC} = 0.1136927$; $K_{F_B} = 9.424319$; $k_{F_B} = 22.745736$

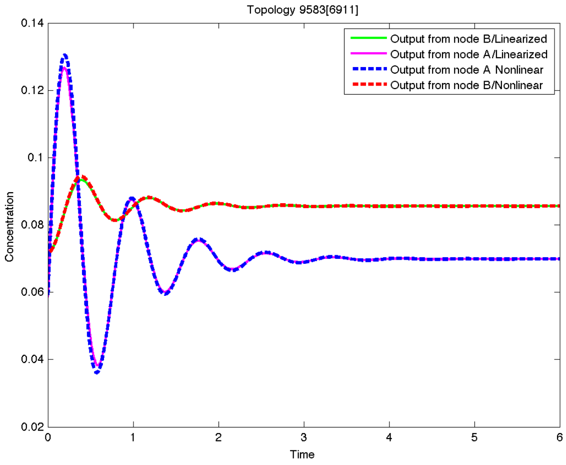

(a) Dynamics of A and B in linearized model

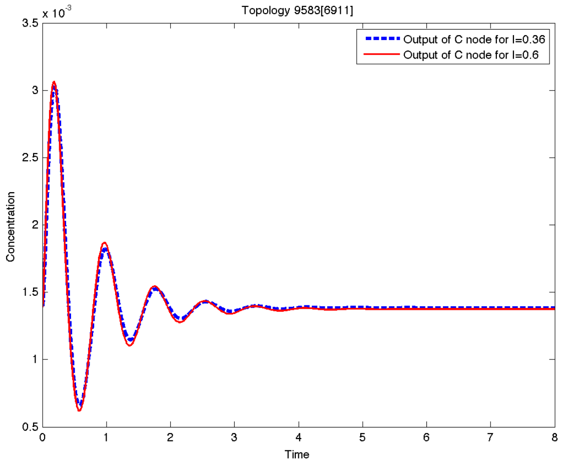

(b) Output from C nonlinear model

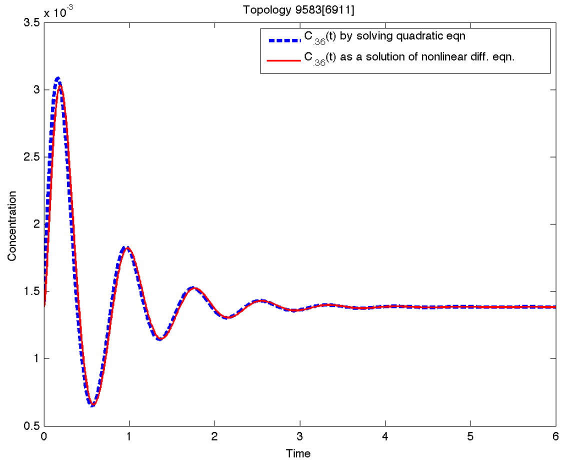

(c) Quadratic approx. and output of nonlinear system, I=0.36

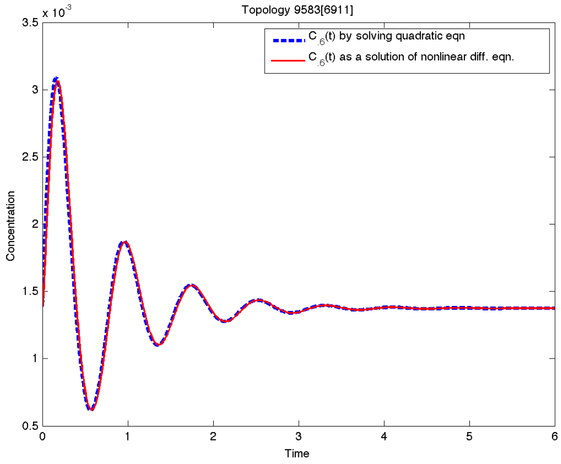

(d) Quadratic approx. and output of nonlinear system, I=0.6

Figure S2. Circuit 1.

Circuit 2.

$$\begin{aligned}
\dot{x}_A &= k_{uA} u \frac{\widetilde{x}_A}{\widetilde{x}_A + K_{uA}} - k_{BA} x_B \frac{x_A}{x_A + K_{BA}} - k_{CA} x_C \frac{x_A}{x_A + K_{CA}} \\
\dot{x}_B &= k_{AB} x_A \frac{\widetilde{x}_B}{\widetilde{x}_B + K_{AB}} - k_{F_B B} x_{F_B} \frac{x_B}{x_B + K_{F_B B}} \\
\dot{x}_C &= k_{AC} x_A \frac{\widetilde{x}_C}{\widetilde{x}_C + K_{AC}} - k_{BC} x_B \frac{x_C}{x_C + K_{BC}}
\end{aligned}$$

Parameters: $K_{uA} = 0.093918$; $k_{uA} = 11.447219$; $K_{BA} = 0.001688$; $k_{BA} = 44.802268$; $K_{CA} = 90.209027$; $k_{CA} = 96.671843$; $K_{AB} = 0.001191$; $k_{AB} = 1.466561$; $K_{F_B} = 9.424319$; $k_{F_B} = 22.745736$; $K_{AC} = 0.113697$; $k_{AC} = 1.211993$; $K_{BC} = 0.009891$; $k_{BC} = 7.239357$

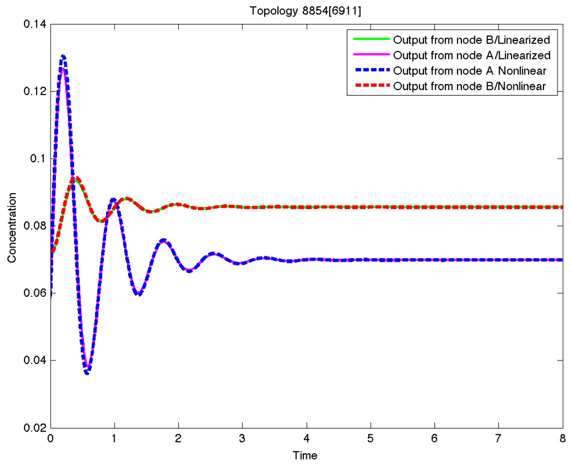

(a) Dynamics of A and B in linearized model

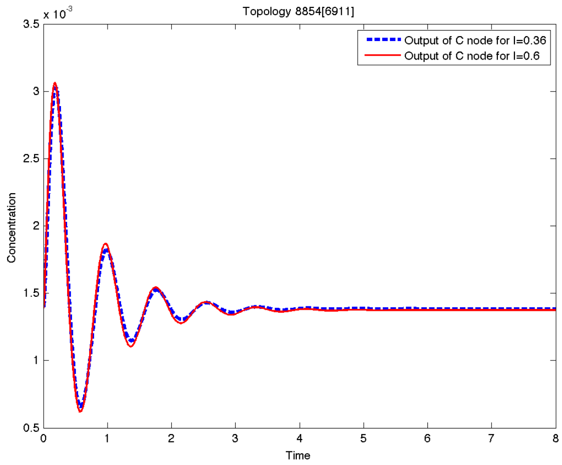

(b) Output from C nonlinear model

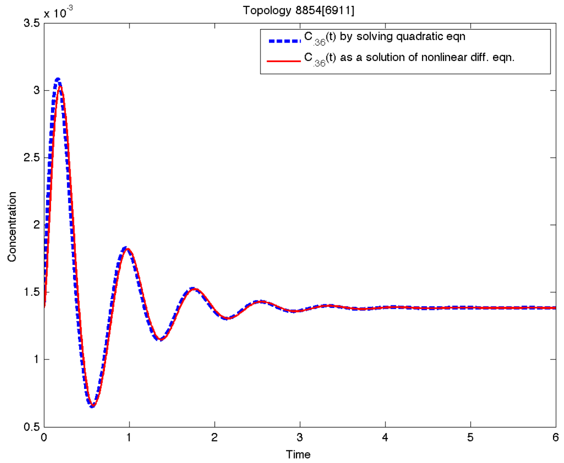

(c) Quadratic approx. and output of nonlinear system

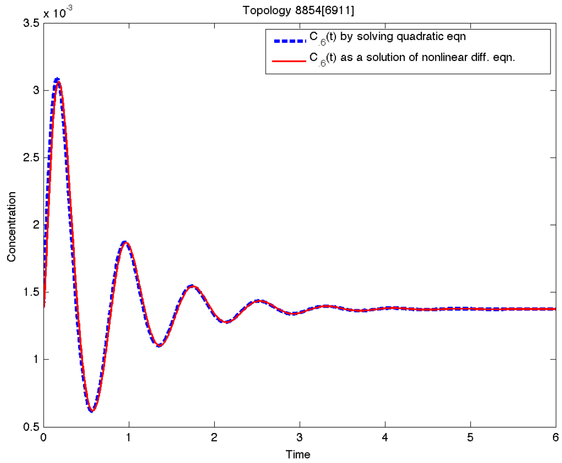

(d) Quadratic approx. and output of nonlinear system

Figure S3. Circuit 2.

Circuit 3.

$$
\begin{aligned}
\dot{x}_A &= k_{uA} u \frac{\widetilde{x}_A}{\widetilde{x}_A + K_{uA}} - k_{BA} x_B \frac{x_A}{x_A + K_{BA}} - k_{AA} x_A \frac{x_A}{x_A + K_{AA}} \\
\dot{x}_B &= k_{AB} x_A \frac{\widetilde{x}_B}{\widetilde{x}_B + K_{AB}} - k_{CB} x_B \frac{x_B}{x_B + K_{CB}} - k_{BB} x_B \frac{x_B}{x_B + K_{BB}} \\
\dot{x}_C &= k_{BC} x_B \frac{\widetilde{x}_C}{\widetilde{x}_C + K_{BC}} - k_{AC} x_A \frac{x_C}{x_C + K_{AC}}
\end{aligned}
$$

Parameters: $K_{AA} = 7.633962$; $k_{AA} = 86.238263$; $K_{AB} = 20.265158$; $k_{AB} = 5.428752$; $K_{AC} = 0.258375$; $k_{AC} = 62.416585$; $K_{BA} = 0.003960$; $k_{BA} = 17.705166$; $K_{BB} = 31.604578$; $k_{BB} = 3.692326$; $K_{BC} = 44.386408$; $k_{BC} = 65.027941$; $K_{CB} = 0.701052$; $k_{CB} = 26.091557$; $K_{uA} = 0.464248$; $k_{uA} = 1.882348$

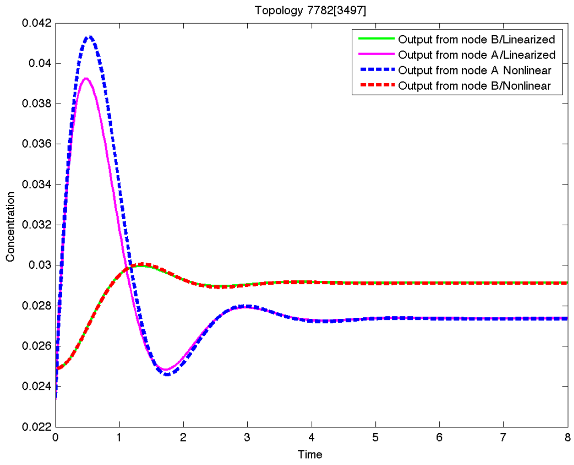

(a) Dynamics of A and B in linearized model

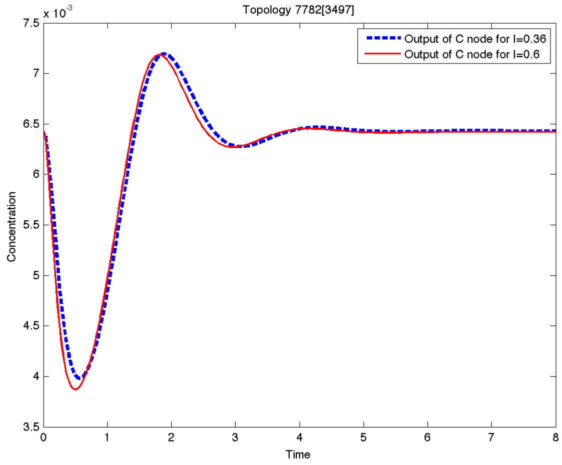

(b) Output from C nonlinear model

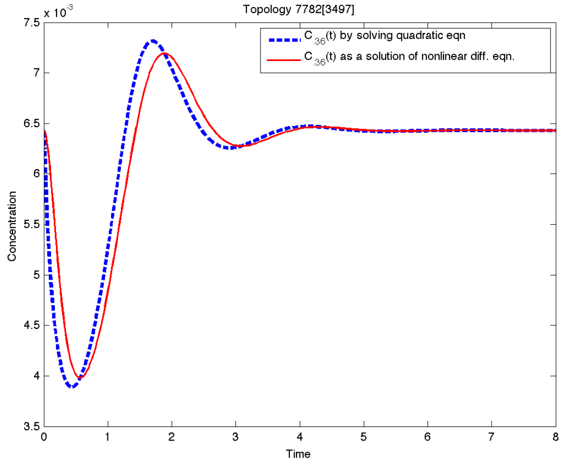

(c) Quadratic approx. and output of nonlinear system

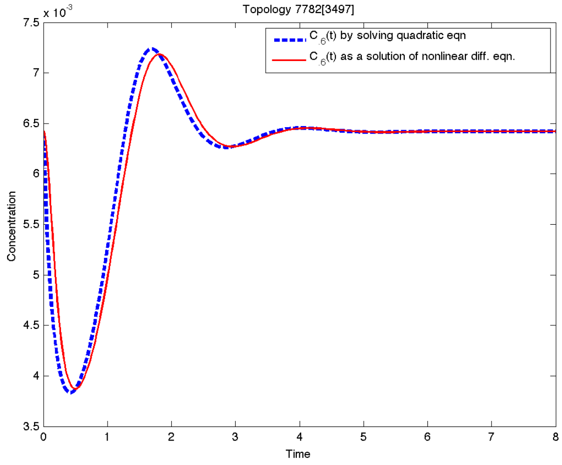

(d) Quadratic approx. and output of nonlinear system

Figure S4. Circuit 3.

Circuit 4.

$$
\begin{aligned}
\dot{x}_A &= k_{uA} u \frac{\widetilde{x}_A}{\widetilde{x}_A + K_{uA}} - k_{BA} x_B \frac{x_A}{x_A + K_{BA}} - k_{AA} x_A \frac{x_A}{x_A + K_{AA}} \\
\dot{x}_B &= k_{AB} x_A \frac{\widetilde{x}_B}{\widetilde{x}_B + K_{AB}} - k_{CB} x_C \frac{x_B}{x_B + K_{CB}} \\
\dot{x}_C &= k_{BC} x_B \frac{\widetilde{x}_C}{\widetilde{x}_C + K_{BC}} - k_{AC} x_A \frac{x_C}{x_C + K_{AC}}
\end{aligned}
$$

Parameters: $K_{AA} = 7.633962$; $k_{AA} = 86.238263$; $K_{AB} = 20.265158$; $k_{AB} = 5.428752$; $K_{AC} = 0.258375$; $k_{AC} = 62.416585$; $K_{BA} = 0.003960$; $k_{BA} = 17.705166$; $K_{BC} = 44.386408$; $k_{BC} = 65.027941$; $K_{CB} = 0.701052$; $k_{CB} = 26.091557$; $K_{uA} = 0.464248$; $k_{uA} = 1.882348$

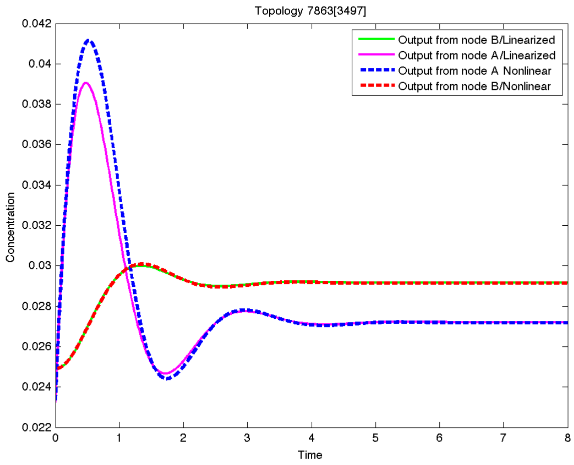

(a) Dynamics of A and B in linearized model

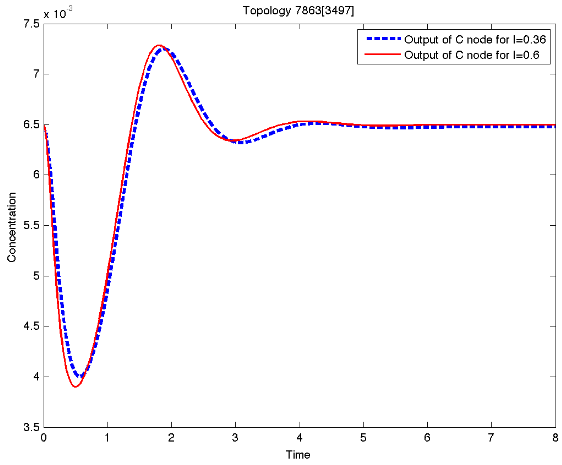

(b) Output from C nonlinear model

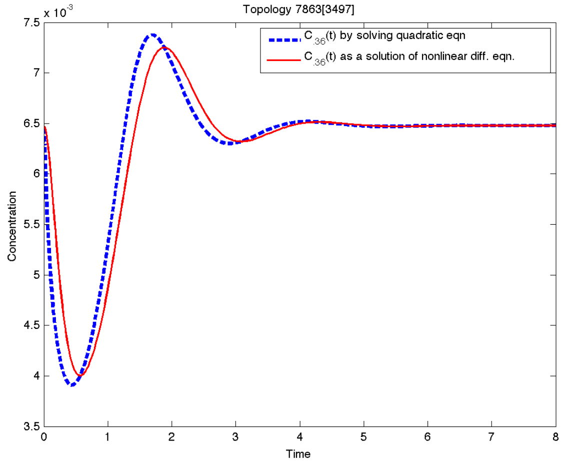

(c) Quadratic approx. and output of nonlinear system

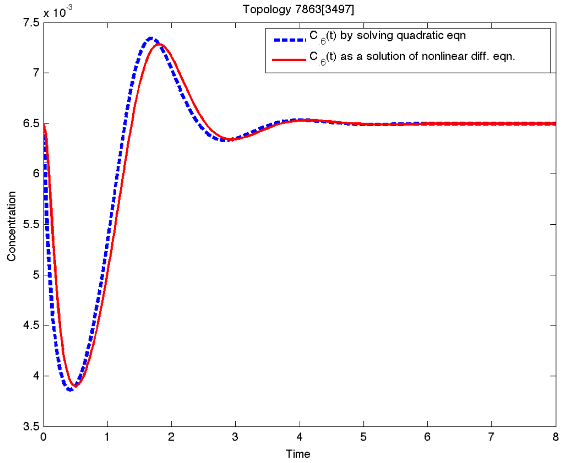

(d) Quadratic approx. and output of nonlinear system

Figure S5. Circuit 4.

Circuit 5.

$$
\begin{aligned}
\dot{x}_A &= k_{uA} u \frac{\widetilde{x}_A}{\widetilde{x}_A + K_{uA}} - k_{BA} x_B \frac{x_A}{x_A + K_{BA}} \\
\dot{x}_B &= k_{AB} x_A \frac{\widetilde{x}_B}{\widetilde{x}_B + K_{AB}} - k_{CB} x_C \frac{x_B}{x_B + K_{CB}} \\
\dot{x}_C &= k_{BC} x_B \frac{\widetilde{x}_C}{\widetilde{x}_C + K_{BC}} - k_{AC} x_A \frac{x_C}{x_C + K_{AC}}
\end{aligned}
$$

Parameters:$K_{AB} = 63.277600$; $k_{AB} = 6.638959$; $K_{AC} = 0.133429$; $k_{AC} = 55.731406$; $K_{BA} = 0.011188$; $k_{BA} = 2.749793$; $K_{BC} = 0.013374$; $k_{BC} = 45.175191$; $K_{CB} = 1.457975$; $k_{CB} = 2.114949$; $K_{uA} = 24.589517$; $k_{uA} = 5.346875$

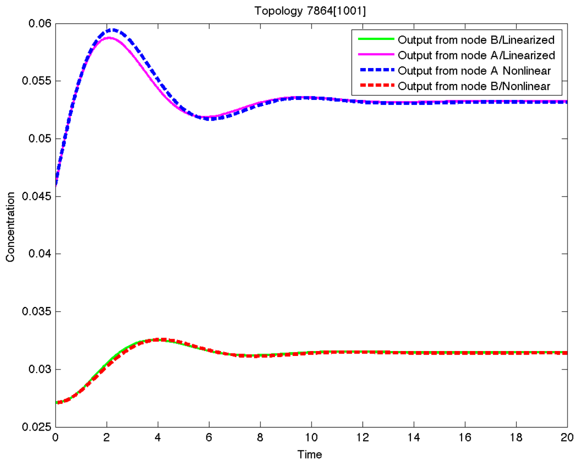

(a) Dynamics of A and B in linearized model

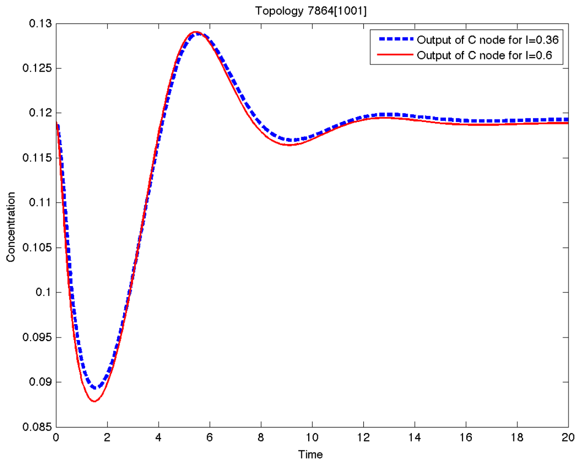

(b) Output from C nonlinear model

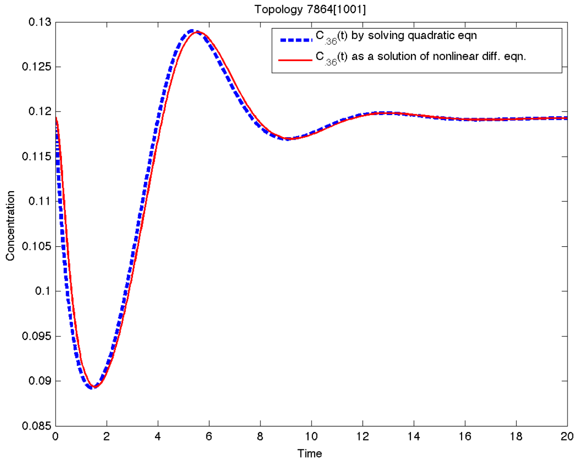

(c) Quadratic approx. and output of nonlinear system

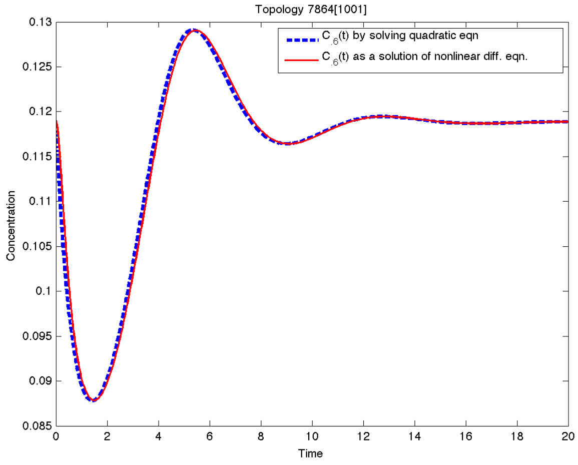

(d) Quadratic approx. and output of nonlinear system

Figure S6. Circuit 5.

Circuit 6.

$$
\begin{aligned}
\dot{x}_A &= k_{uA}u\frac{\widetilde{x}_A}{\widetilde{x}_A + K_{uA}} - k_{BA}x_B\frac{x_A}{x_A + K_{BA}} - k_{AA}x_A\frac{x_A}{x_A + K_{AA}} - k_{CA}x_C\frac{x_A}{x_A + K_{CA}} \\
\dot{x}_B &= k_{AB}x_A\frac{\widetilde{x}_B}{\widetilde{x}_B + K_{AB}} - k_{CB}x_C\frac{x_B}{x_B + K_{CB}} - k_{BB}x_B\frac{x_B}{x_B + K_{BB}} \\
\dot{x}_C &= k_{BC}x_B\frac{\widetilde{x}_C}{\widetilde{x}_C + K_{BC}} - k_{AC}x_A\frac{x_C}{x_C + K_{AC}}
\end{aligned}
$$

Parameters: $K_{AA} = 7.633962$; $k_{AA} = 86.238263$; $K_{AB} = 20.265158$; $k_{AB} = 5.428752$; $K_{AC} = 0.258375$; $k_{AC} = 62.416585$; $K_{BA} = 0.003960$; $k_{BA} = 17.705166$; $K_{BB} = 31.604578$; $k_{BB} = 3.692326$; $K_{BC} = 44.386408$; $k_{BC} = 65.027941$; $K_{CA} = 26.714681$; $k_{CA} = 2.806080$; $K_{CB} = 0.701052$; $k_{CB} = 26.091557$; $K_{uA} = 0.464248$; $k_{uA} = 1.882348$

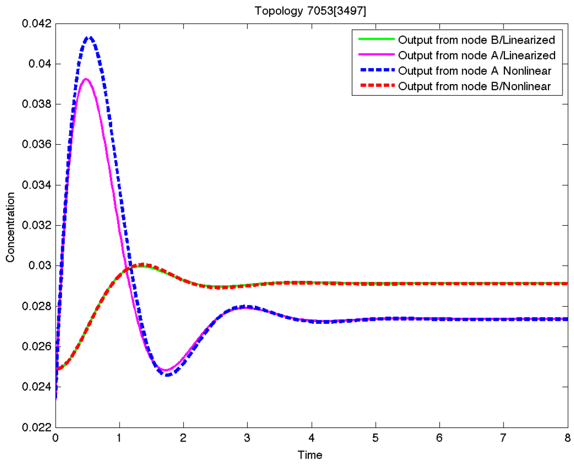

(a) Dynamics of A and B in linearized model

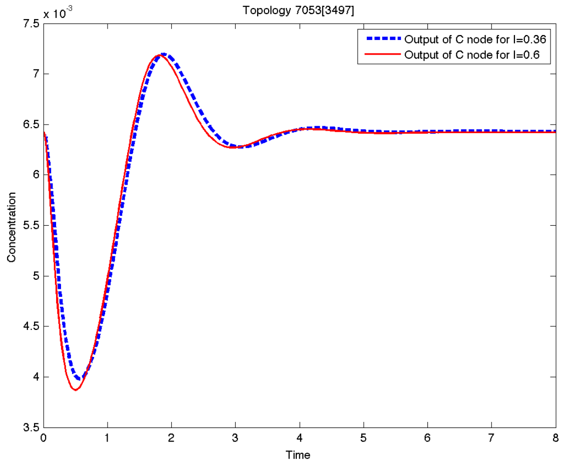

(b) Output from C nonlinear model

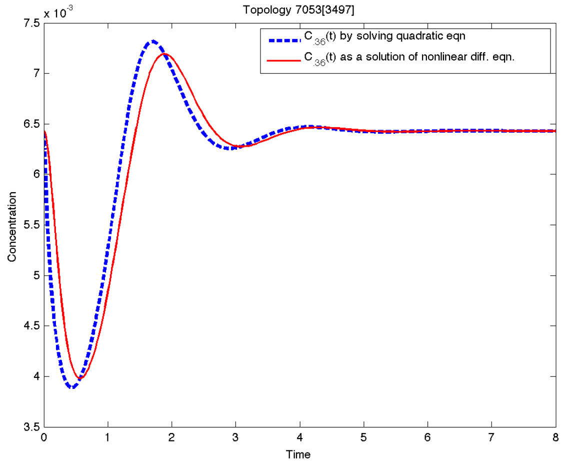

(c) Quadratic approx. and output of nonlinear system

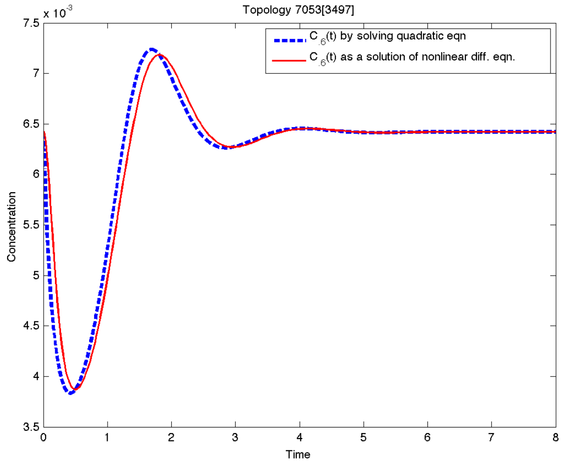

(d) Quadratic approx. and output of nonlinear system

Figure S7. Circuit 6.

Circuit 7.

$$\dot{x}_A = k_{uA}u\frac{\widetilde{x}_A}{\widetilde{x}_A + K_{uA}} - k_{BA}x_B\frac{x_A}{x_A + K_{BA}} - k_{AA}x_A\frac{x_A}{x_A + K_{AA}} - k_{CA}x_C\frac{x_A}{x_A + K_{CA}}$$
$$\dot{x}_B = k_{AB}x_A\frac{\widetilde{x}_B}{\widetilde{x}_B + K_{AB}} - k_{CB}x_C\frac{x_B}{x_B + K_{CB}}$$
$$\dot{x}_C = k_{BC}x_B\frac{\widetilde{x}_C}{\widetilde{x}_C + K_{BC}} - k_{AC}x_A\frac{x_C}{x_C + K_{AC}}$$

Parameters: $K_{AA} = 7.633962$; $k_{AA} = 86.238263$; $K_{AB} = 20.265158$; $k_{AB} = 5.428752$; $K_{AC} = 0.258375$; $k_{AC} = 62.416585$; $K_{BA} = 0.003960$; $k_{BA} = 17.705166$; $K_{BC} = 44.386408$; $k_{BC} = 65.027941$; $K_{CA} = 26.714681$; $k_{CA} = 2.806080$; $K_{CB} = 0.701052$; $k_{CB} = 26.091557$; $K_{uA} = 0.464248$; $k_{uA} = 1.882348$

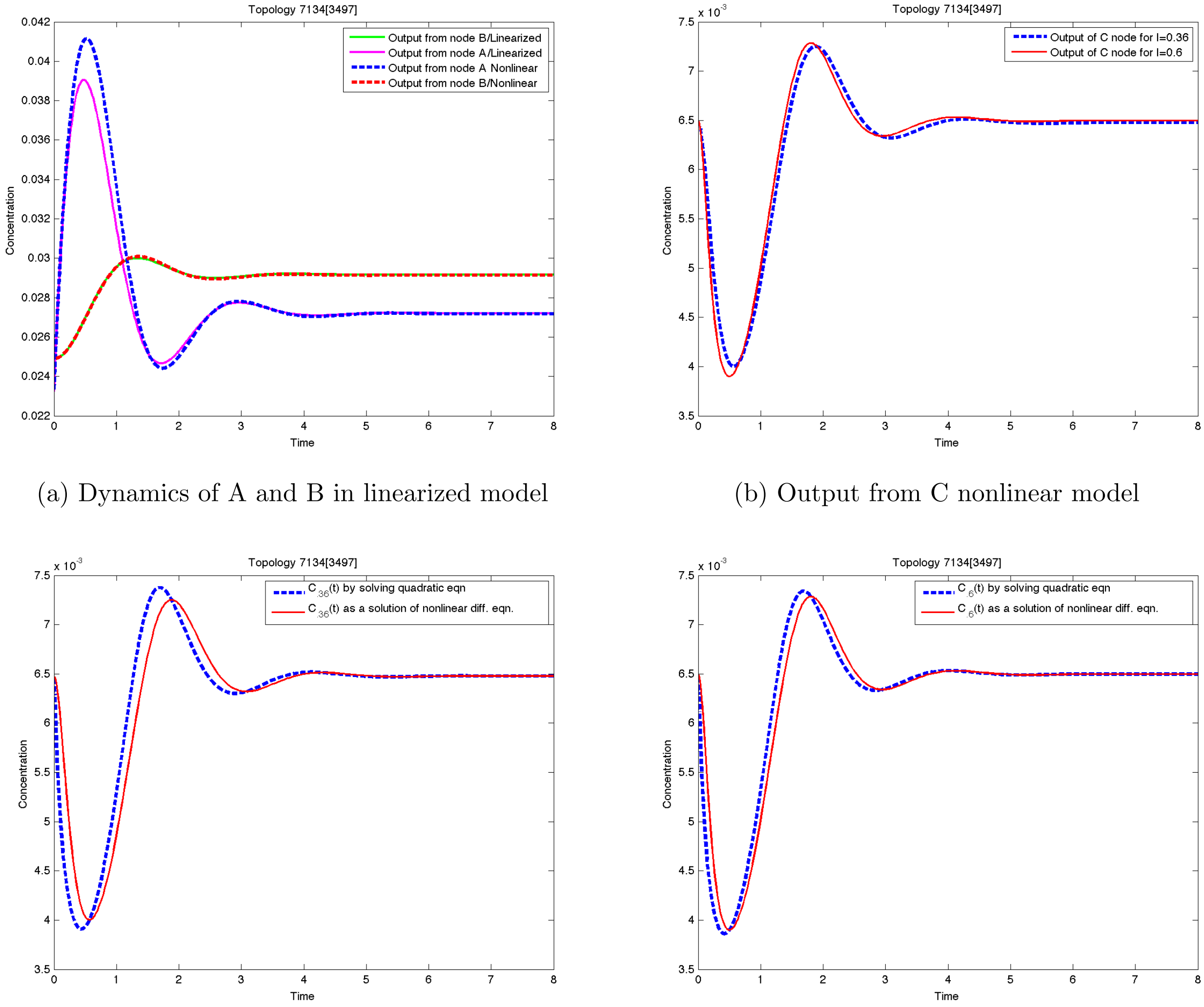

(a) Dynamics of A and B in linearized model

(b) Output from C nonlinear model

(c) Quadratic approx. and output of nonlinear system

(d) Quadratic approx. and output of nonlinear system

Figure S8. Circuit 7.

Circuit 8.

$$
\begin{aligned}
\dot{x}_A &= k_{uA} u \frac{\widetilde{x}_A}{\widetilde{x}_A + K_{uA}} - k_{BA} x_B \frac{x_A}{x_A + K_{BA}} \\
\dot{x}_B &= k_{AB} x_A \frac{\widetilde{x}_B}{\widetilde{x}_B + K_{AB}} - k_{F_B B} x_{F_B} \frac{x_B}{x_B + K_{F_B B}} + k_{CB} x_C \frac{\widetilde{x}_B}{\widetilde{x}_B + K_{CB}} \\
\dot{x}_C &= k_{AC} x_A \frac{\widetilde{x}_C}{\widetilde{x}_C + K_{AC}} - k_{BC} x_B \frac{x_C}{x_C + K_{BC}} - k_{CC} x_C \frac{x_C}{x_C + K_{CC}}
\end{aligned}
$$

Parameters: $K_{uA} = 0.093918$; $k_{uA} = 11.447219$; $K_{BA} = 0.001688$; $k_{BA} = 44.802268$; $K_{AB} = 0.001191$; $k_{AB} = 1.466561$; $K_{F_B} = 9.424319$; $k_{F_B} = 22.745736$; $K_{AC} = 0.113697$; $k_{AC} = 1.211993$; $K_{BC} = 0.009891$; $k_{BC} = 7.239357$; $K_{CB} = 30.602013$; $k_{CB} = 3.811536$; $K_{CC} = 0.189125$; $k_{CC} = 17.910182$

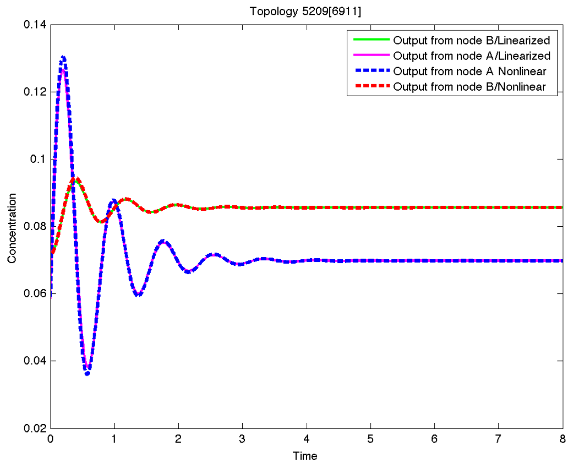

(a) Dynamics of A and B in linearized model

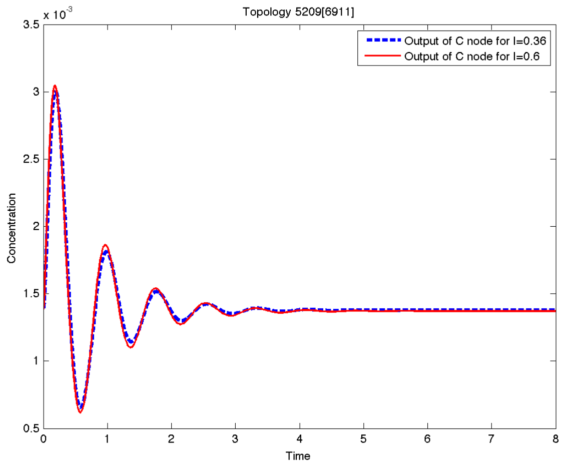

(b) Output from C nonlinear model

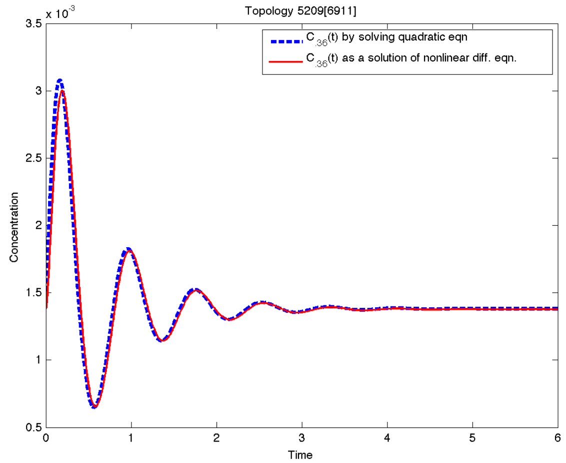

(c) Quadratic approx. and output of nonlinear system

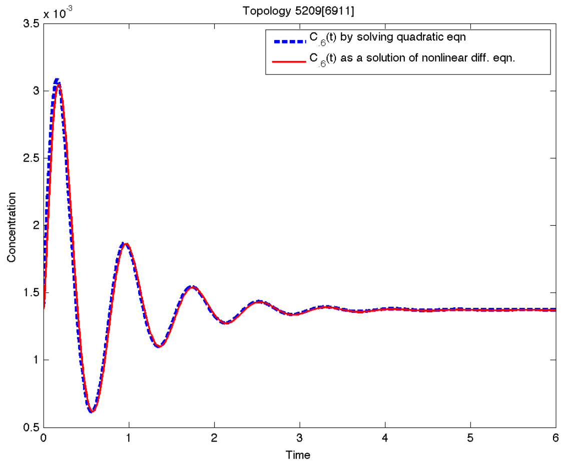

(d) Quadratic approx. and output of nonlinear system

Figure S9. Circuit 8.

Circuit 9.

$$
\begin{aligned}
\dot{x}_A &= k_{uA} u \frac{\widetilde{x}_A}{\widetilde{x}_A + K_{uA}} - k_{BA} x_B \frac{x_A}{x_A + K_{BA}} - k_{CA} x_C \frac{x_A}{x_A + K_{CA}} \\
\dot{x}_B &= k_{AB} x_A \frac{\widetilde{x}_B}{\widetilde{x}_B + K_{AB}} + k_{CB} x_C \frac{\widetilde{x}_B}{\widetilde{x}_B + K_{CB}} - k_{F_B B} x_{F_B} \frac{x_B}{x_B + K_{F_B B}} \\
\dot{x}_C &= k_{AC} x_A \frac{\widetilde{x}_C}{\widetilde{x}_C + K_{AC}} - k_{BC} x_B \frac{x_C}{x_C + K_{BC}} - k_{CC} x_C \frac{x_C}{x_C + K_{CC}}
\end{aligned}
$$

Parameters: $K_{uA} = 0.093918$; $k_{uA} = 11.447219$; $K_{BA} = 0.001688$; $k_{BA} = 44.802268$; $K_{CA} = 90.209027$; $k_{CA} = 96.671843$; $K_{AB} = 0.001191$; $k_{AB} = 1.466561$; $K_{F_B} = 9.424319$; $k_{F_B} = 22.745736$; $K_{Ac} = 0.113697$; $k_{AC} = 1.211993$; $K_{BC} = 0.009891$; $k_{BC} = 7.239357$; $K_{CB} = 30.602013$; $k_{CB} = 3.811536$; $K_{CC} = 0.189125$; $k_{CC} = 17.910182$

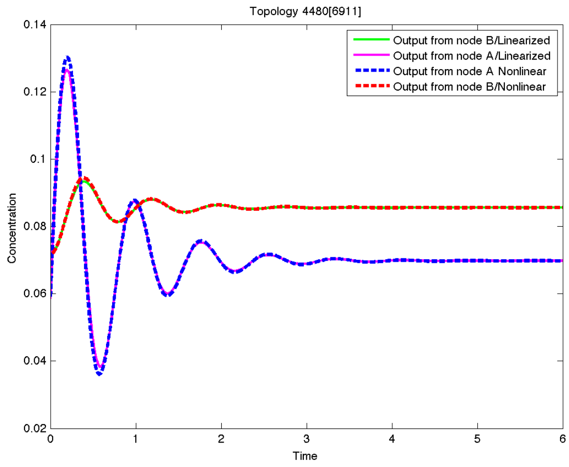

(a) Dynamics of A and B in linearized model

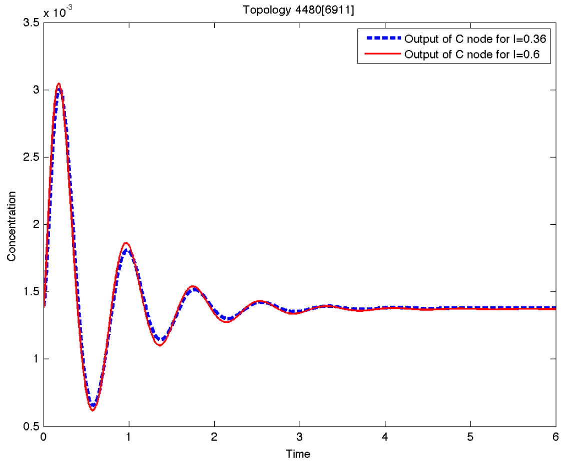

(b) Output from C nonlinear model

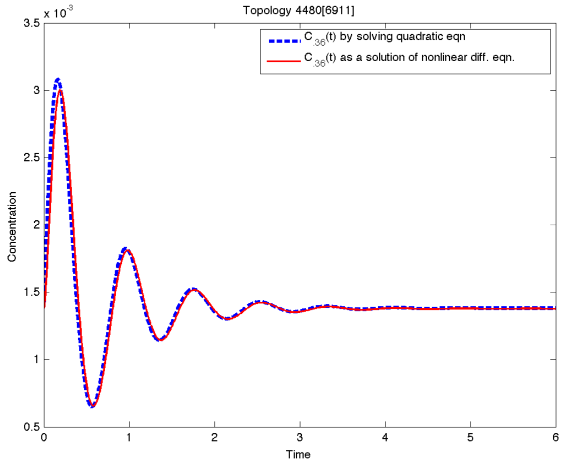

(c) Quadratic approx. and output of nonlinear system

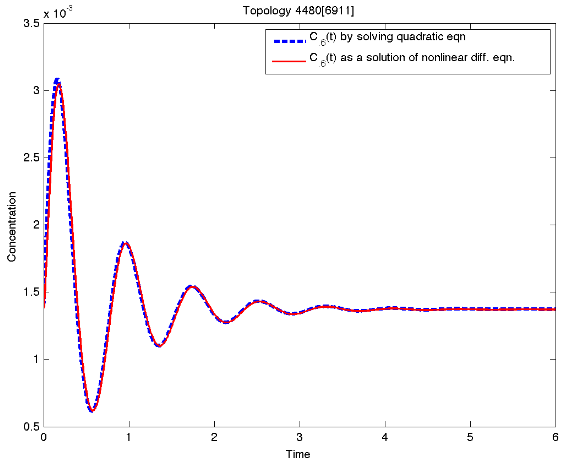

(d) Quadratic approx. and output of nonlinear system

Figure S10. Circuit 9.

Circuit 10.

$$\dot{x}_A = k_{uA}u\frac{\widetilde{x}_A}{\widetilde{x}_A + K_{uA}} - k_{BA}x_B\frac{x_A}{x_A + K_{BA}} - k_{AA}x_A\frac{x_A}{x_A + K_{AA}}$$

$$\dot{x}_B = k_{AB}x_A\frac{\widetilde{x}_B}{\widetilde{x}_B + K_{AB}} + k_{CB}x_C\frac{\widetilde{x}_B}{\widetilde{x}_B + K_{CB}} - k_{F_B B}x_{F_B}\frac{x_B}{x_B + K_{F_B B}}$$

$$\dot{x}_C = k_{BC}x_B\frac{\widetilde{x}_C}{\widetilde{x}_C + K_{BC}} - k_{AC}x_A\frac{x_C}{x_C + K_{AC}} - k_{CC}x_C\frac{x_C}{x_C + K_{CC}}$$

Parameters: $K_{AA} = 24.989065$; $k_{AA} = 53.174082$; $K_{AB} = 0.444375$; $k_{AB} = 12.053134$; $K_{F_B} = 1.716920$; $k_{F_B} = 11.601122$; $K_{AC} = 0.013988$; $k_{AC} = 8.521185$; $K_{BA} = 0.005461$; $k_{BA} = 7.103952$; $K_{BC} = 51.850148$; $k_{BC} = 80.408137$; $K_{CB} = 5.392001$; $k_{CB} = 3.086740$; $K_{CC} = 1.962230$; $k_{CC} = 17.382010$; $K_{uA} = 4.387832$; $k_{uA} = 19.638124$

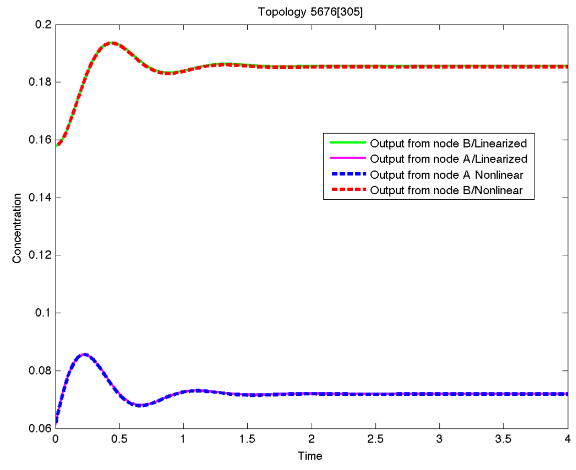

(a) Dynamics of A and B in linearized model

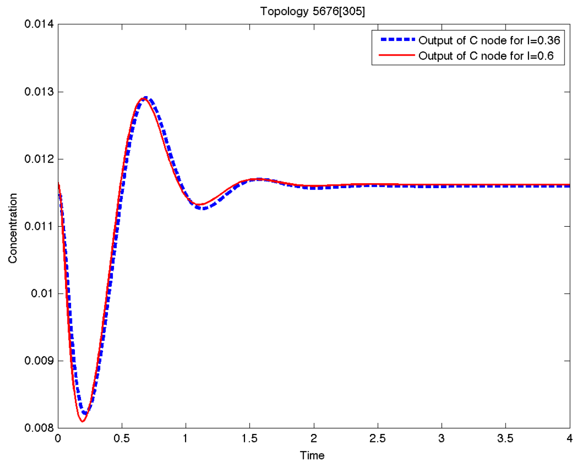

(b) Output from C nonlinear model

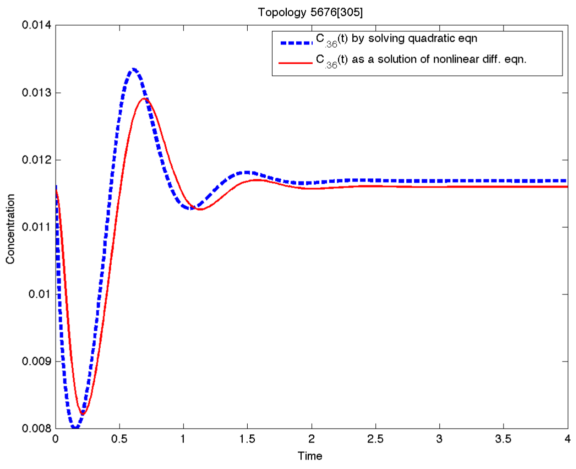

(c) Quadratic approx. and output of nonlinear system

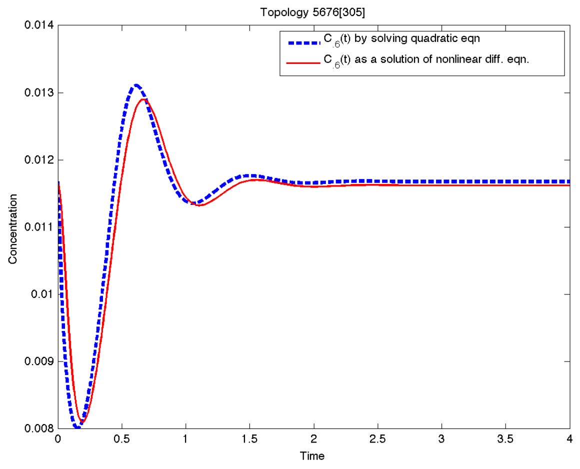

(d) Quadratic approx. and output of nonlinear system

Figure S11. Circuit 10.

Circuit 11.

$$
\begin{aligned}
\dot{x}_A &= k_{uA} u \frac{\widetilde{x}_A}{\widetilde{x}_A + K_{uA}} - k_{BA} x_B \frac{x_A}{x_A + K_{BA}} \\
\dot{x}_B &= k_{AB} x_A \frac{\widetilde{x}_B}{\widetilde{x}_B + K_{AB}} + k_{CB} x_C \frac{\widetilde{x}_B}{\widetilde{x}_B + K_{CB}} - k_{F_B B} x_{F_B} \frac{x_B}{x_B + K_{F_B B}} \\
\dot{x}_C &= k_{BC} x_B \frac{\widetilde{x}_C}{\widetilde{x}_C + K_{BC}} - k_{AC} x_A \frac{x_C}{x_C + K_{AC}} - k_{CC} x_C \frac{x_C}{x_C + K_{CC}}
\end{aligned}
$$

Parameters: $K_{AB} = 0.444375$; $k_{AB} = 12.053134$; $K_{F_B} = 1.716920$; $k_{F_B} = 11.601122$; $K_{AC} = 0.013988$; $k_{AC} = 8.521185$; $K_{BA} = 0.005461$; $k_{BA} = 7.103952$; $K_{BC} = 51.850148$; $k_{BC} = 80.408137$; $K_{CB} = 5.392001$; $k_{CB} = 3.086740$; $K_{CC} = 1.962230$; $k_{CC} = 17.382010$; $K_{uA} = 4.387832$; $k_{uA} = 19.638124$

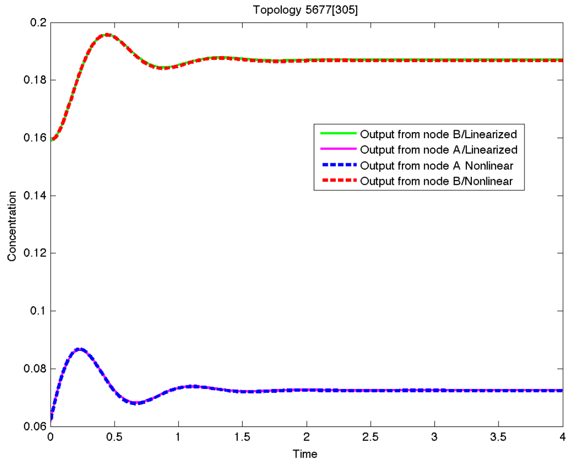

(a) Dynamics of A and B in linearized model

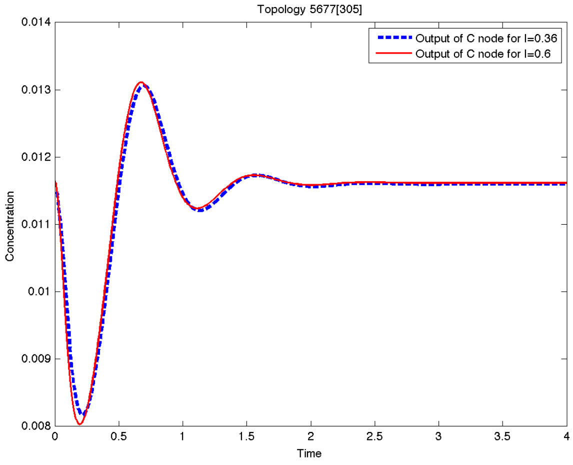

(b) Output from C nonlinear model

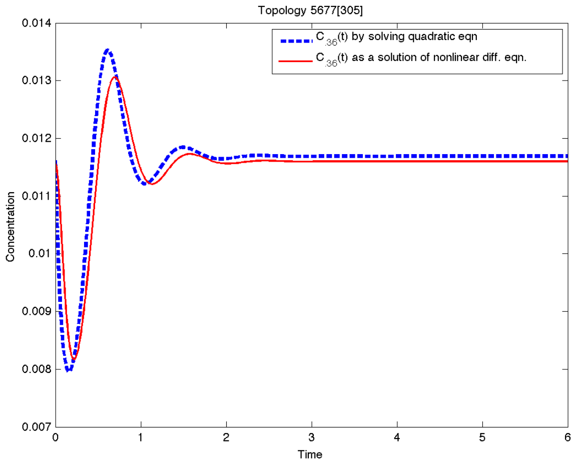

(c) Quadratic approx. and output of nonlinear system

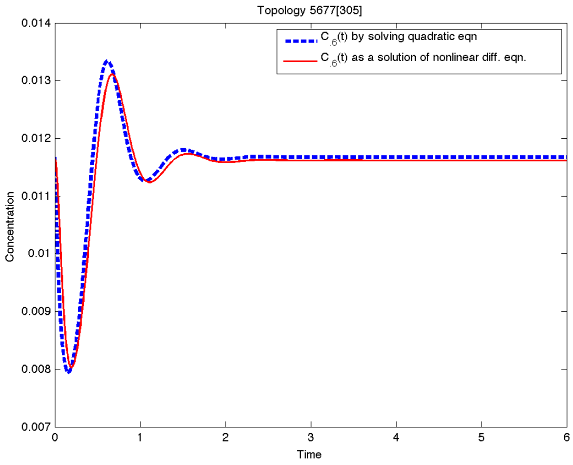

(d) Quadratic approx. and output of nonlinear system

Figure S12. Circuit 11.

Circuit 12.

$$
\begin{aligned}
\dot{x}_A &= k_{uA}u\frac{\widetilde{x}_A}{\widetilde{x}_A + K_{uA}} - k_{BA}x_B\frac{x_A}{x_A + K_{BA}} + k_{CA}x_C\frac{\widetilde{x}_A}{\widetilde{x}_A + K_{CA}} \\
\dot{x}_B &= k_{AB}x_A\frac{\widetilde{x}_B}{\widetilde{x}_B + K_{AB}} + k_{CB}C\frac{\widetilde{x}_B}{\widetilde{x}_B + K_{CB}} - k_{F_B B}x_{F_B}\frac{x_B}{x_B + K_{F_B B}} \\
\dot{x}_C &= k_{AC}x_A\frac{\widetilde{x}_C}{\widetilde{x}_C + K_{AC}} - k_{BC}x_B\frac{x_C}{x_C + K_{BC}} - k_{CC}x_C\frac{x_C}{x_C + K_{CC}}
\end{aligned}
$$

Parameters: $K_{uA} = 0.093918$; $k_{uA} = 11.447219$; $K_{BA} = 0.001688$; $k_{BA} = 44.802268$; $K_{CA} = 5.026318$; $k_{CA} = 45.803641$; $K_{AB} = 0.001191$; $k_{AB} = 1.466561$; $K_{F_B} = 9.424319$; $k_{F_B} = 22.745736$; $K_{AC} = 0.113697$; $k_{AC} = 1.211993$; $K_{BC} = 0.009891$; $k_{BC} = 7.239357$; $K_{CB} = 30.602013$; $k_{CB} = 3.811536$; $K_{CC} = 0.189125$; $k_{CC} = 17.910182$

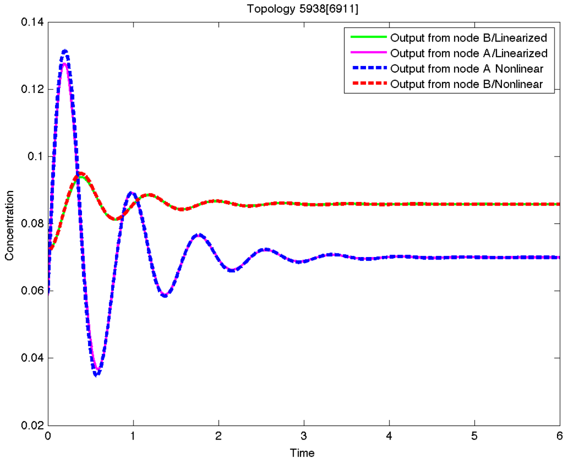

(a) Dynamics of A and B in linearized model

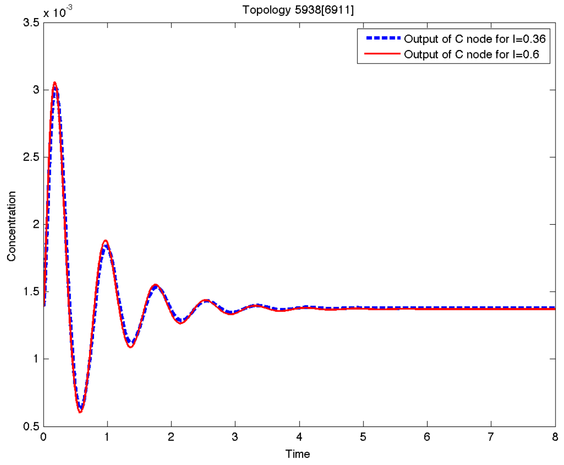

(b) Output from C nonlinear model

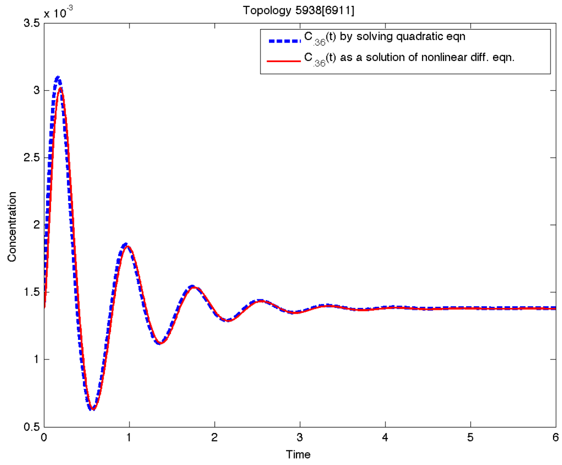

(c) Quadratic approx. and output of nonlinear system

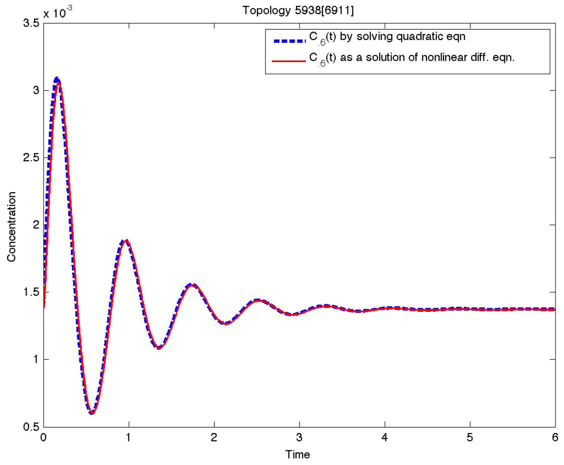

(d) Quadratic approx. and output of nonlinear system

Figure S13. Circuit 12.

Circuit 13.

$$
\begin{aligned}
\dot{x}_A &= k_{uA}u\frac{\widetilde{x}_A}{\widetilde{x}_A + K_{uA}} - k_{BA}x_B\frac{x_A}{x_A + K_{BA}} - k_{AA}x_A\frac{x_A}{x_A + K_{AA}} + k_{CA}x_C\frac{\widetilde{x}_A}{\widetilde{x}_A + K_{CA}} \\
\dot{x}_B &= k_{AB}x_A\frac{\widetilde{x}_B}{\widetilde{x}_B + K_{AB}} + k_{CB}x_C\frac{\widetilde{x}_B}{\widetilde{x}_B + K_{CB}} - k_{BB}x_B\frac{x_B}{x_B + K_{BB}} \\
\dot{x}_C &= k_{BC}x_B\frac{\widetilde{x}_C}{\widetilde{x}_C + K_{BC}} - k_{AC}x_A\frac{x_C}{x_C + K_{AC}} - k_{CC}x_A\frac{x_C}{x_C + K_{CC}}
\end{aligned}
$$

Parameters: $K_{AA} = 24.989065$; $k_{AA} = 53.174082$; $K_{AB} = 0.444375$; $k_{AB} = 12.053134$; $K_{F_B} = 1.716920$; $k_{F_B} = 11.601122$; $K_{AC} = 0.013988$; $k_{AC} = 8.521185$; $K_{BA} = 0.005461$; $k_{BA} = 7.103952$; $K_{BC} = 51.850148$; $k_{BC} = 80.408137$; $K_{CB} = 5.392001$; $k_{CB} = 3.086740$; $K_{CC} = 1.962230$; $k_{CC} = 17.382010$; $K_{uA} = 4.387832$; $k_{uA} = 19.638124$; $K_{CA} = 15.479253$; $k_{CA} = 4.903430$

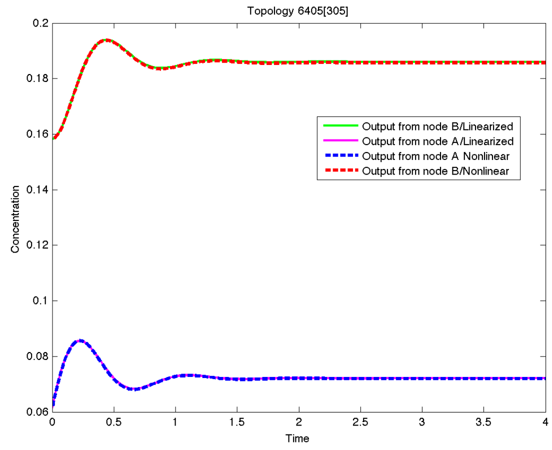

(a) Dynamics of A and B in linearized model

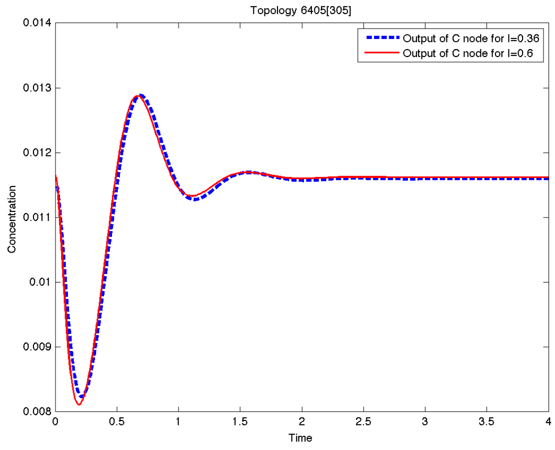

(b) Output from C nonlinear model

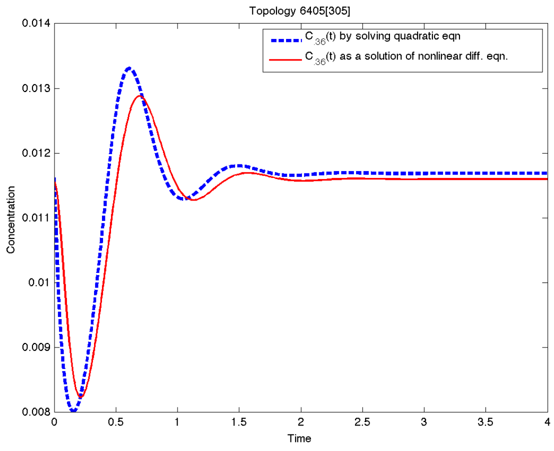

(c) Quadratic approx. and output of nonlinear system

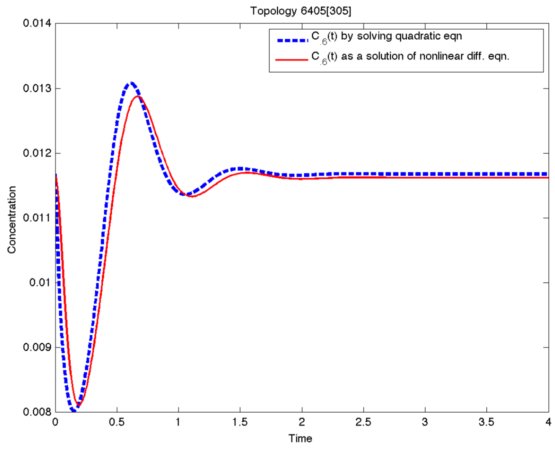

(d) Quadratic approx. and output of nonlinear system

Figure S14. Circuit 13.

Circuit 14.

$$
\begin{aligned}
\dot{x}_A &= k_{uA}u\frac{\widetilde{x}_A}{\widetilde{x}_A + K_{uA}} - k_{BA}x_B\frac{x_A}{x_A + K_{BA}} + k_{CA}x_C\frac{\widetilde{x}_A}{\widetilde{x}_A + K_{CA}} \\
\dot{x}_B &= k_{AB}x_A\frac{\widetilde{x}_B}{\widetilde{x}_B + K_{AB}} + k_{CB}x_C\frac{\widetilde{x}_B}{\widetilde{x}_B + K_{CB}} - k_{BB}x_B\frac{x_B}{x_B + K_{BB}} \\
\dot{x}_C &= k_{BC}x_B\frac{\widetilde{x}_C}{\widetilde{x}_C + K_{BC}} - k_{AC}x_A\frac{x_C}{x_C + K_{AC}} - k_{CC}x_A\frac{x_C}{x_C + K_{CC}}
\end{aligned}
$$

Parameters: $K_{AB} = 0.444375$; $k_{AB} = 12.053134$; $K_{F_B}1.716920$; $k_{F_B} = 11.601122$; $K_{AC} = 0.013988$; $k_{AC} = 8.521185$; $K_{BA} = 0.005461$; $k_{BA} = 7.103952$; $K_{BC} = 51.850148$; $k_{BC} = 80.408137$; $K_{CB} = 5.392001$; $k_{CB} = 3.086740$; $K_{CC} = 1.962230$; $k_{CC} = 17.382010$; $K_{uA} = 4.387832$; $k_{uA} = 19.638124$; $K_{CA} = 15.479253$; $k_{CA} = 4.903430$

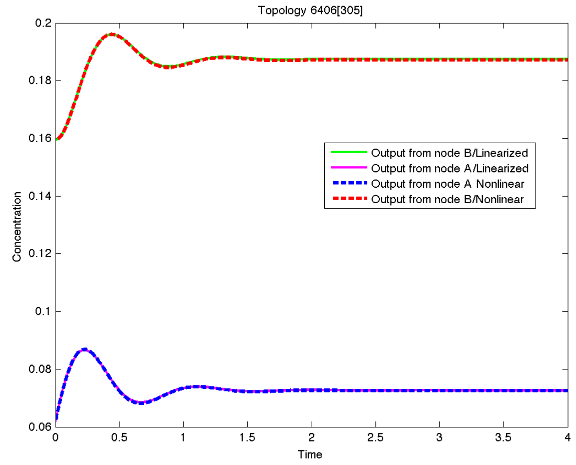

(a) Dynamics of A and B in linearized model

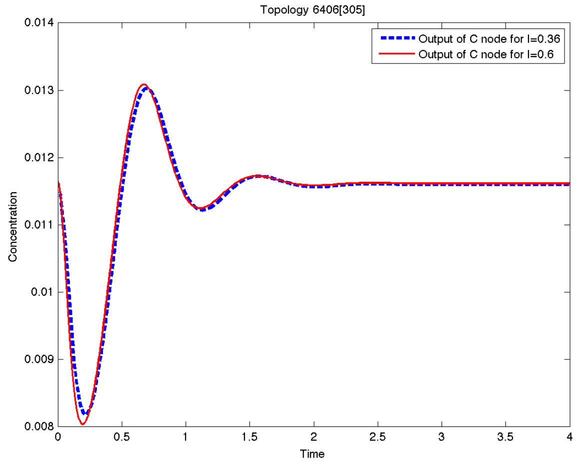

(b) Output from C nonlinear model

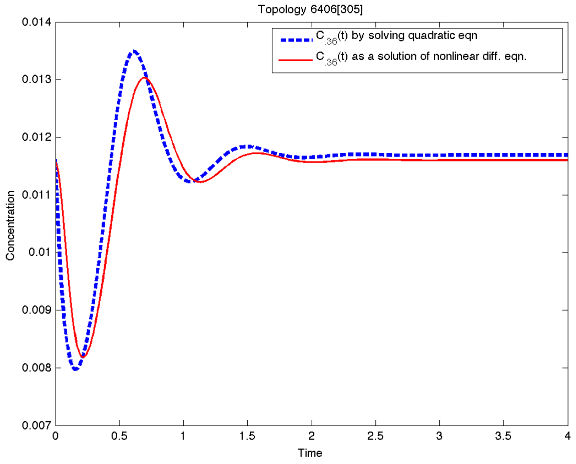

(c) Quadratic approx. and output of nonlinear system

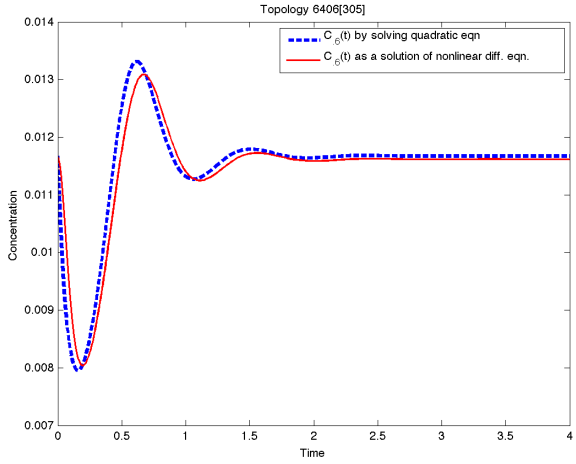

(d) Quadratic approx. and output of nonlinear system

Figure S15. Circuit 14.

Circuit 15.

$$
\begin{aligned}
\dot{x}_A &= k_{uA}u\frac{\widetilde{x}_A}{\widetilde{x}_A + K_{uA}} - k_{BA}x_B\frac{x_A}{x_A + K_{BA}} \\
\dot{x}_B &= k_{AB}x_A\frac{\widetilde{x}_B}{\widetilde{x}_B + K_{AB}} - k_{F_B B}x_{F_B}\frac{x_B}{x_B + K_{F_B B}} \\
\dot{x}_C &= k_{AC}x_A\frac{\widetilde{x}_C}{\widetilde{x}_C + K_{AC}} - k_{BC}x_B\frac{x_C}{x_C + K_{BC}} - k_{CC}x_A\frac{x_C}{x_C + K_{CC}}
\end{aligned}
$$

Parameters: $K_{AB} = 0.709169$; $k_{AB} = 7.445605$; $K_{F_B} = 1.495375$; $k_{F_B} = 7.282827$; $K_{AC} = 0.002566$; $k_{AC} = 1.115065$; $K_{BA} = 0.002522$; $k_{BA} = 5.753075$; $K_{BC} = 0.017051$; $k_{BC} = 2.777794$; $K_{CC} = 0.195997$; $k_{CC} = 1.480130$; $K_{uA} = 0.225814$; $k_{uA} = 2.492872$

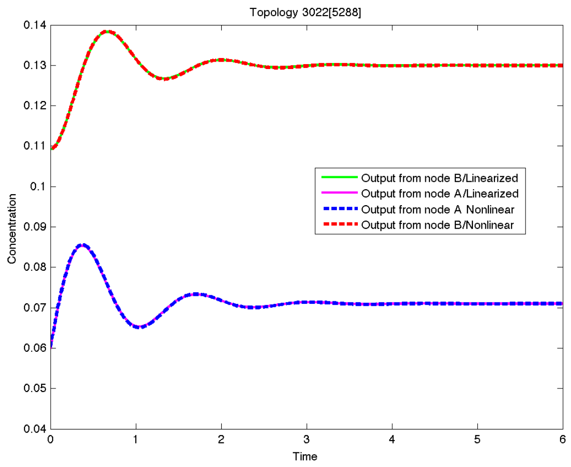

(a) Dynamics of A and B in linearized model

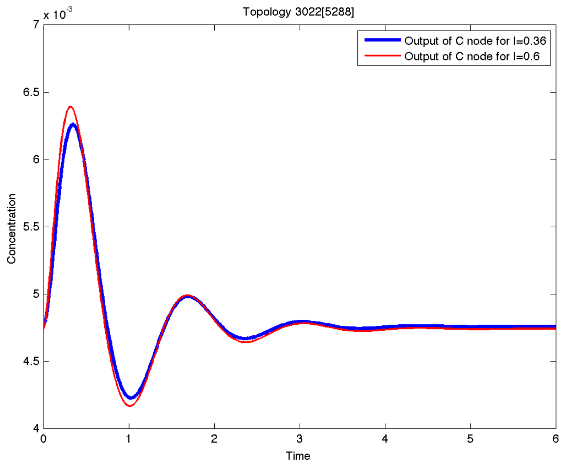

(b) Output from C nonlinear model

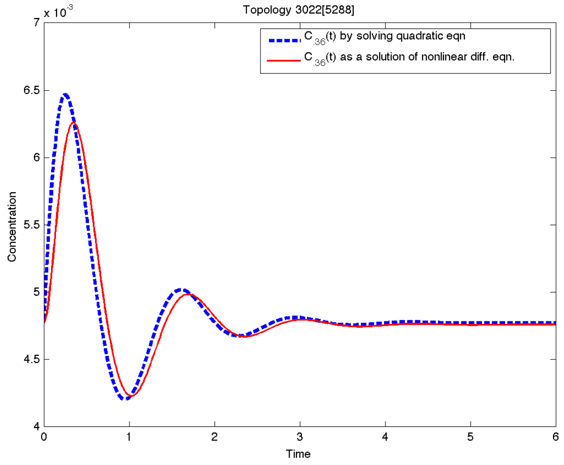

(c) Quadratic approx. and output of nonlinear system

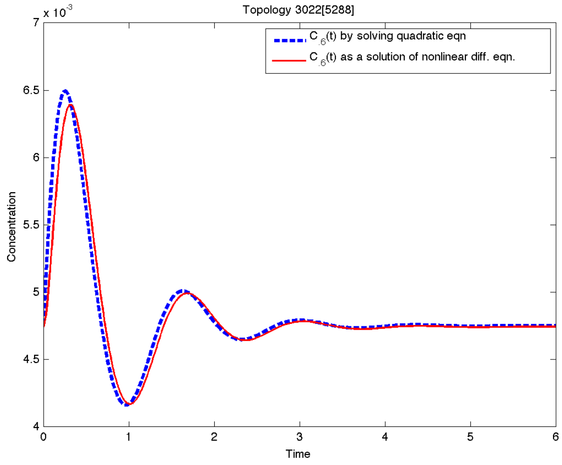

(d) Quadratic approx. and output of nonlinear system

Figure S16. Circuit 15.

Circuit 16.

This is the same topology as in the previous case, only a different parameter set was used:

Parameters: $K_{AB} = 0.001191$; $k_{AB} = 1.466561$; $K_{F_B} = 9.424319$; $k_{F_B} = 22.745736$; $K_{AC} = 0.113697$; $k_{AC} = 1.211993$; $K_{BA} = 0.001688$; $k_{BA} = 44.802268$; $K_{BC} = 0.009891$; $k_{BC} = 7.239357$; $K_{CC} = 0.189125$; $k_{CC} = 17.910182$; $K_{uA} = 0.093918$; $k_{uA} = 11.447219$

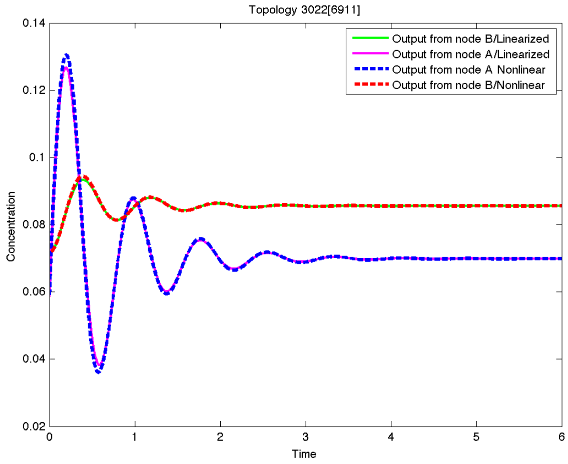

(a) Dynamics of A and B in linearized model

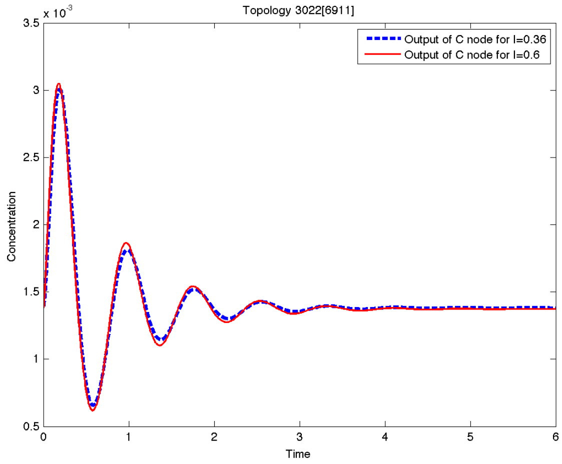

(b) Output from C nonlinear model

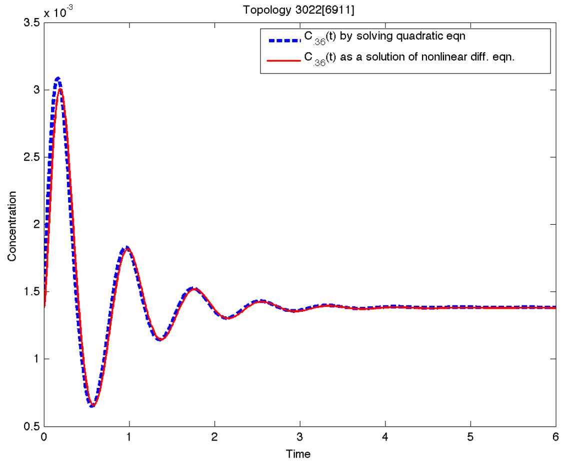

(c) Quadratic approx. and output of nonlinear system

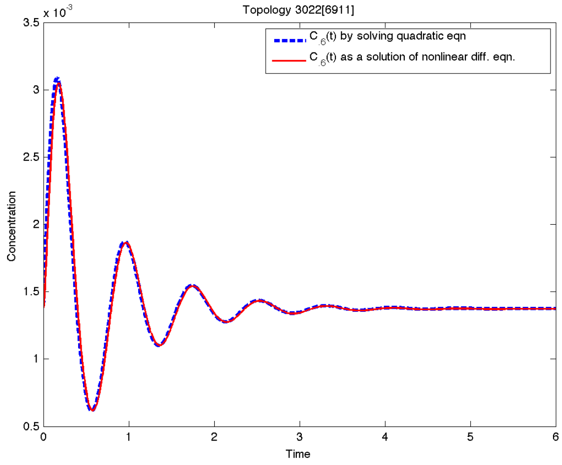

(d) Quadratic approx. and output of nonlinear system

Figure S17. Circuit 16.

Circuit 17.

This is the same topology as in the previous case, only a different parameter set was used:

Parameters: $K_{AB} = 1.620877$; $k_{AB} = 2.306216$; $K_{F_B} = 2.012565$; $k_{F_B} = 2.700847$; $K_{AC} = 0.010933$; $k_{AC} = 8.968091$; $K_{BA} = 0.001812$; $k_{BA} = 10.039221$; $K_{BC} = 0.014199$; $k_{BC} = 17.762333$; $K_{CC} = 2.686891$; $k_{CC} = 4.139044$; $K_{uA} = 0.161715$; $k_{uA} = 1.933303$

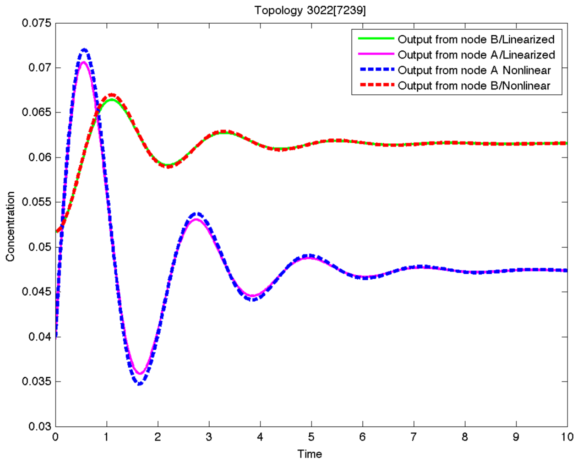

(a) Dynamics of A and B in linearized model

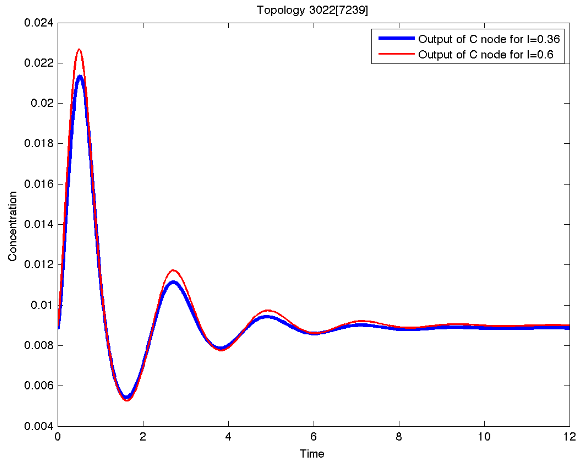

(b) Output from C nonlinear model

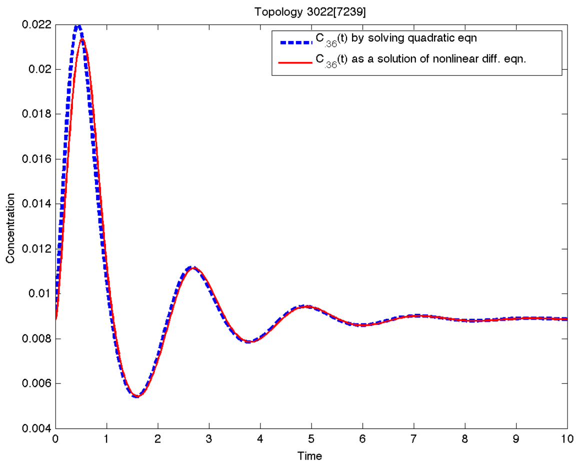

(c) Quadratic approx. and output of nonlinear system

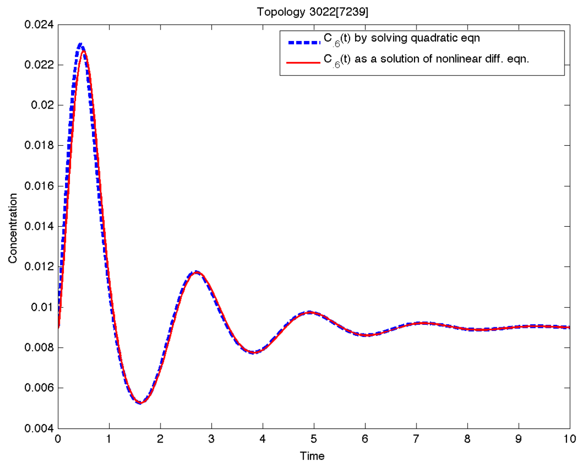

(d) Quadratic approx. and output of nonlinear system

Figure S18. Circuit 17.

Circuit 18.

$$\begin{aligned}
\dot{x}_A &= k_{uA} u \frac{\widetilde{x}_A}{\widetilde{x}_A + K_{uA}} - k_{BA} x_B \frac{x_A}{x_A + K_{BA}} - k_{AA} x_A \frac{x_A}{x_A + K_{AA}} \\
\dot{x}_B &= k_{AB} x_A \frac{\widetilde{x}_B}{\widetilde{x}_B + K_{AB}} + k_{BB} x_B \frac{\widetilde{x}_B}{\widetilde{x}_B + K_{BB}} - k_{F_B B} x_{F_B} \frac{x_B}{x_B + K_{F_B B}} \\
\dot{x}_C &= k_{AC} x_A \frac{\widetilde{x}_C}{\widetilde{x}_C + K_{AC}} - k_{BC} x_B \frac{x_C}{x_C + K_{BC}} - k_{CC} x_C \frac{x_C}{x_C + K_{CC}}
\end{aligned}$$

Parameters: $K_{AA} = 17.569120$; $k_{AA} = 2.198366$; $K_{AB} = 9.435176$; $k_{AB} = 3.134007$; $K_{F_B} = 0.469083$; $k_{F_B} = 1.934194$; $K_{AC} = 0.062914$; $k_{AC} = 2.742206$; $K_{BA} = 0.003245$; $k_{BA} = 75.352905$; $K_{BB} = 27.463128$; $k_{BB} = 10.551155$; $K_{BC} = 0.041615$; $k_{BC} = 61.333818$; $K_{CC} = 0.039332$; $k_{CC} = 4.756637$; $K_{uA} = 0.005167$; $k_{uA} = 8.186533$

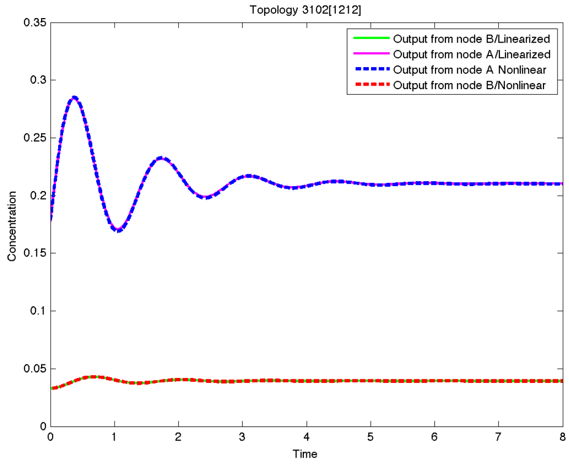

(a) Dynamics of A and B in linearized model

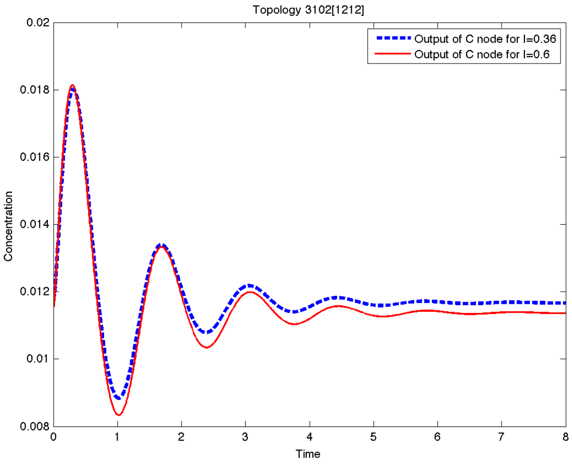

(b) Output from C nonlinear model

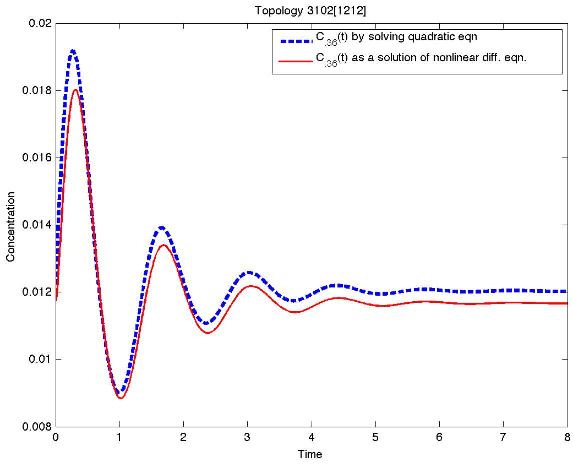

(c) Quadratic approx. and output of nonlinear system

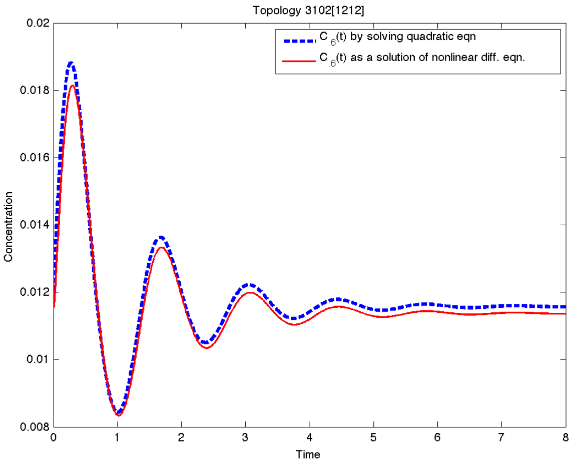

(d) Quadratic approx. and output of nonlinear system

Figure S19. Circuit 18.

Circuit 19.

$$\dot{x}_A = k_{uA} u \frac{\widetilde{x}_A}{\widetilde{x}_A + K_{uA}} - k_{BA} x_B \frac{x_A}{x_A + K_{BA}} - k_{AA} x_A \frac{x_A}{x_A + K_{AA}}$$

$$\dot{x}_B = k_{AB} x_A \frac{\widetilde{x}_B}{\widetilde{x}_B + K_{AB}} - k_{F_B B} x_{F_B} \frac{x_B}{x_B + K_{F_B B}}$$

$$\dot{x}_C = k_{BC} x_B \frac{\widetilde{x}_C}{\widetilde{x}_C + K_{BC}} - k_{AC} x_A \frac{x_C}{x_C + K_{AC}} - k_{CC} x_C \frac{x_C}{x_C + K_{CC}}$$

Parameters: $K_{uA} = 4.387832$; $k_{uA} = 19.638124$; $K_{BA} = 0.005461$; $k_{BA} = 7.103952$; $K_{AA} = 24.989065$; $k_{AA} = 53.174082$; $K_{AB} = 0.444375$; $k_{AB} = 12.053134$; $K_{F_B} = 1.716920$; $k_{F_B} = 11.601122$; $K_{BC} = 51.850148$; $k_{BC} = 80.408137$; $K_{AC} = 0.013988$; $k_{AC} = 8.521185$; $K_{CC} = 1.962230$; $k_{CC} = 17.382010$

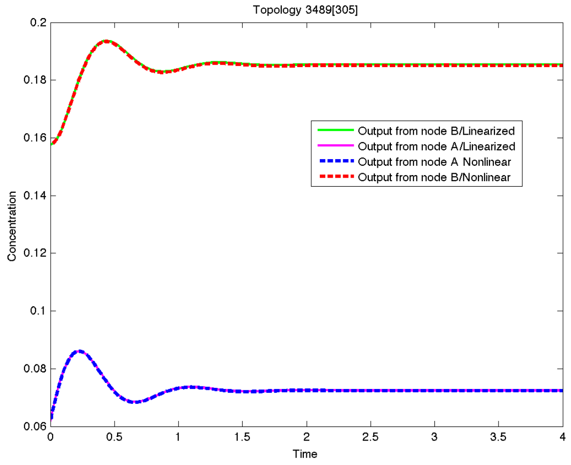

(a) Dynamics of A and B in linearized model

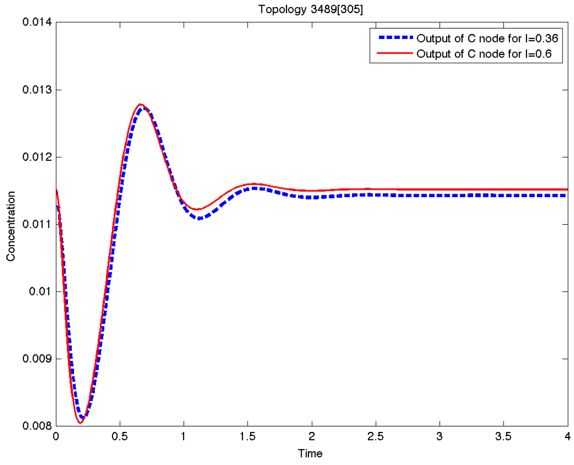

(b) Output from C nonlinear model

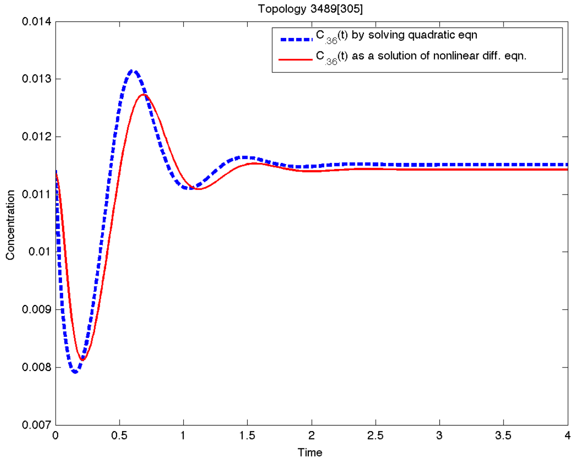

(c) Quadratic approx. and output of nonlinear system

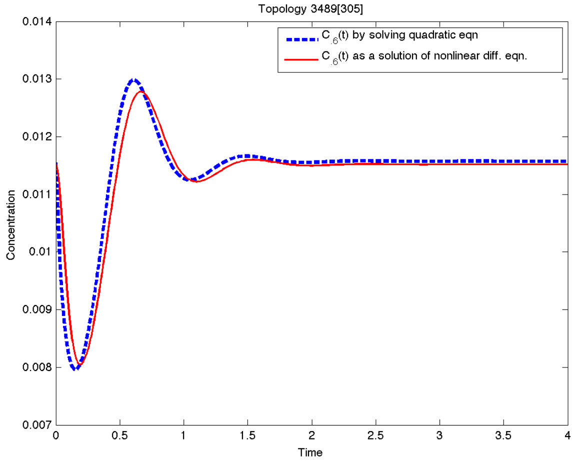

(d) Quadratic approx. and output of nonlinear system

Figure S20. Circuit 19.

Circuit 20.

$$\dot{x}_A = k_{uA}u\frac{\widetilde{x}_A}{\widetilde{x}_A + K_{uA}} - k_{BA}x_B\frac{x_A}{x_A + K_{BA}}$$
$$\dot{x}_B = k_{AB}x_A\frac{\widetilde{x}_B}{\widetilde{x}_B + K_{AB}} - k_{F_BB}x_{F_B}\frac{x_B}{x_B + K_{F_BB}}$$
$$\dot{x}_C = k_{BC}x_B\frac{\widetilde{x}_C}{\widetilde{x}_C + K_{BC}} - k_{AC}x_A\frac{x_C}{x_C + K_{AC}} - k_{CC}x_C\frac{x_C}{x_C + K_{CC}}$$

Parameters: $K_{uA} = 4.387832$; $k_{uA} = 19.638124$; $K_{BA} = 0.005461$; $k_{BA} = 7.103952$; $K_{AB} = 0.444375$; $k_{AB} = 12.053134$; $K_{F_B} = 1.716920$; $k_{F_B} = 11.601122$; $K_{BC} = 51.850148$; $k_{BC} = 80.408137$; $K_{AC} = 0.013988$; $k_{AC} = 8.521185$; $K_{CC} = 1.962230$; $k_{CC} = 17.382010$

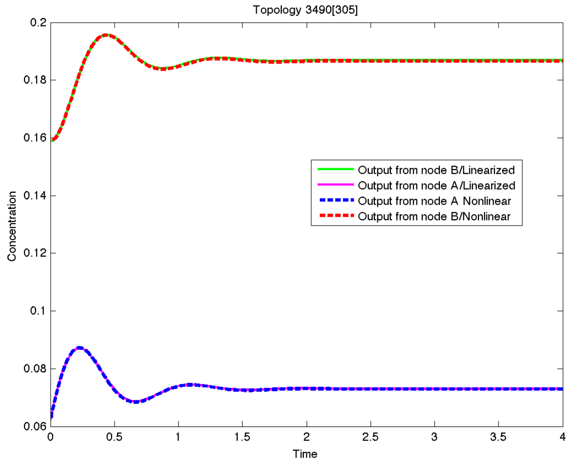

(a) Dynamics of A and B in linearized model

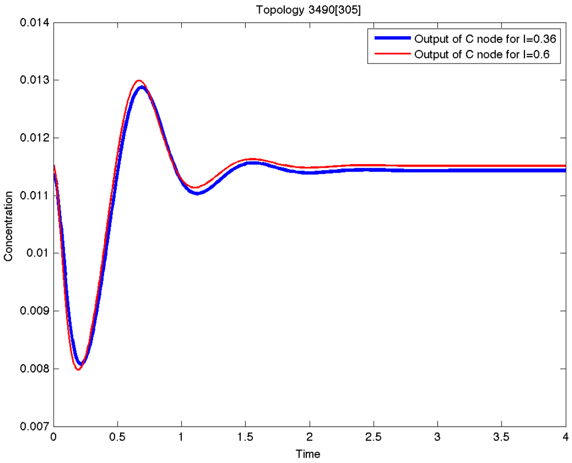

(b) Output from C nonlinear model

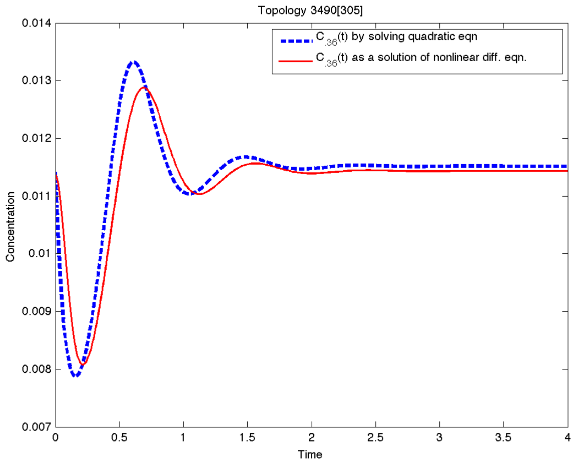

(c) Quadratic approx. and output of nonlinear system

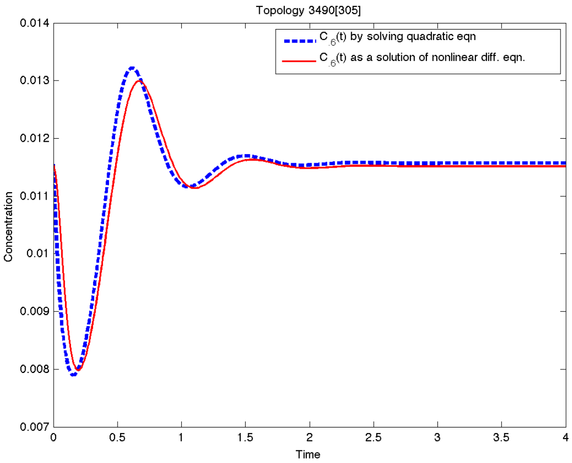

(d) Quadratic approx. and output of nonlinear system

Figure S21. Circuit 20.

Circuit 21.

$$
\begin{aligned}
\dot{x}_A &= k_{uA} u \frac{\widetilde{x}_A}{\widetilde{x}_A + K_{uA}} - k_{BA} x_B \frac{x_A}{x_A + K_{BA}} + k_{CA} x_C \frac{\widetilde{x}_A}{\widetilde{x}_A + K_{CA}} \\
\dot{x}_B &= k_{AB} x_A \frac{\widetilde{x}_B}{\widetilde{x}_B + K_{AB}} - k_{F_B B} x_{F_B} \frac{x_B}{x_B + K_{F_B B}} \\
\dot{x}_C &= k_{AC} x_A \frac{\widetilde{x}_C}{\widetilde{x}_C + K_{AC}} - k_{BC} x_B \frac{x_C}{x_C + K_{BC}} - k_{CC} x_C \frac{x_C}{x_C + K_{CC}}
\end{aligned}
$$

Parameters: $K_{uA} = 0.093918$; $k_{uA} = 11.447219$; $K_{BA} = 0.001688$; $k_{BA} = 44.802268$; $K_{CA} = 5.026318$; $k_{CA} = 45.803641$; $K_{AB} = 0.001191$; $k_{AB} = 1.466561$; $K_{F_B} = 9.424319$; $k_{F_B} = 22.745736$; $K_{AC} = 0.113697$; $k_{AC} = 1.211993$; $K_{BC} = 0.009891$; $k_{BC} = 7.239357$; $K_{CC} = 0.189125$; $k_{CC} = 17.910182$

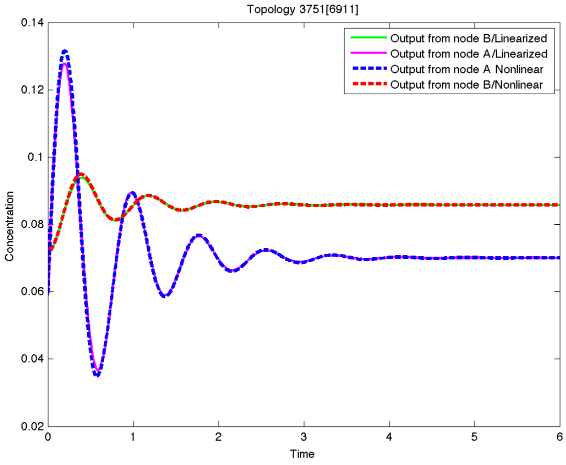

(a) Dynamics of A and B in linearized model

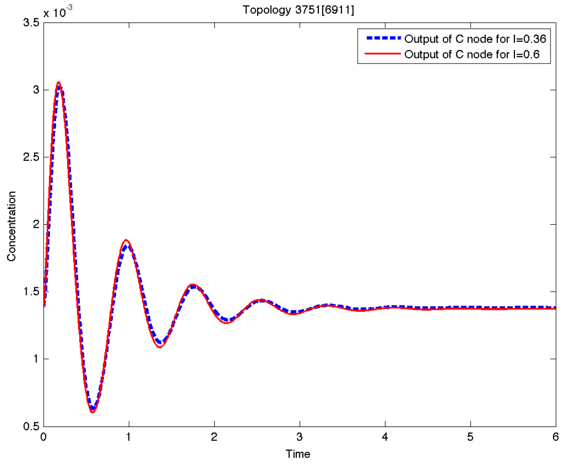

(b) Output from C nonlinear model

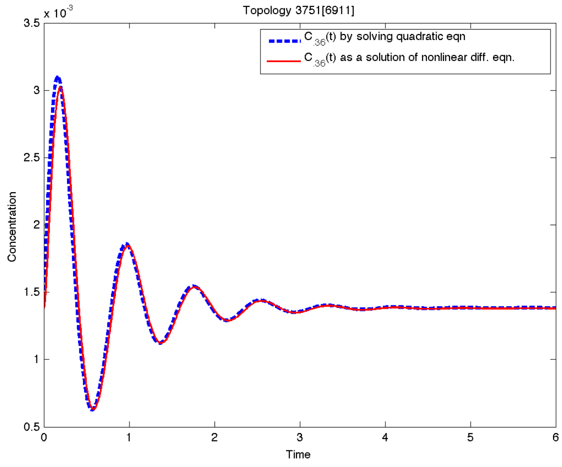

(c) Quadratic approx. and output of nonlinear system

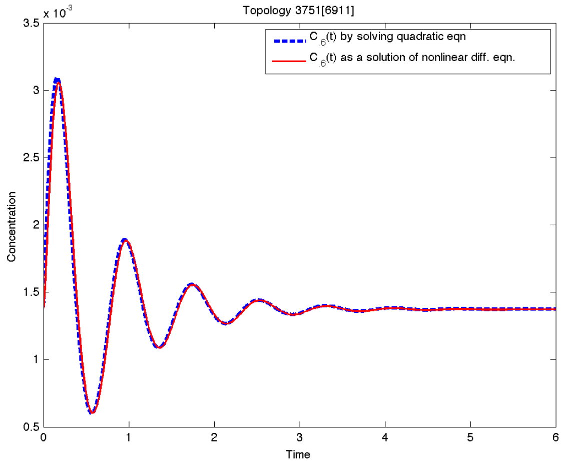

(d) Quadratic approx. and output of nonlinear system

Figure S22. Circuit 21.

Circuit 22.

This is the same topology as in the previous case, only a different parameter set was used:

Parameters: $K_{AB} = 1.620877$; $k_{AB} = 2.306216$; $K_{F_B} = 2.012565$; $k_{F_B} = 2.700847$; $K_{AC} = 0.010933$; $k_{AC} = 8.968091$; $K_{BA} = 0.001812$; $k_{BA} = 10.039221$; $K_{BC} = 0.014199$; $k_{BC} = 17.762333$; $K_{CA} = 0.002690$; $k_{CA} = 1.506954$; $K_{CC} = 2.686891$; $k_{CC} = 4.139044$; $K_{uA} = 0.161715$; $k_{uA} = 1.933303$

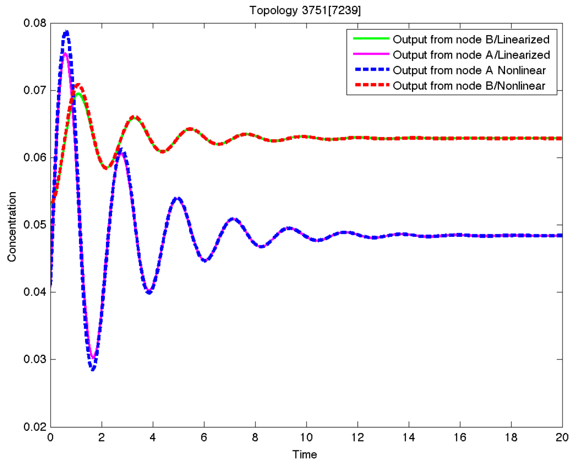

(a) Dynamics of A and B in linearized model

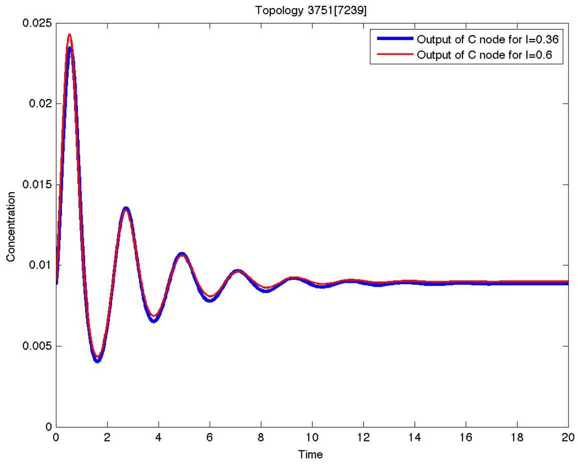

(b) Output from C nonlinear model

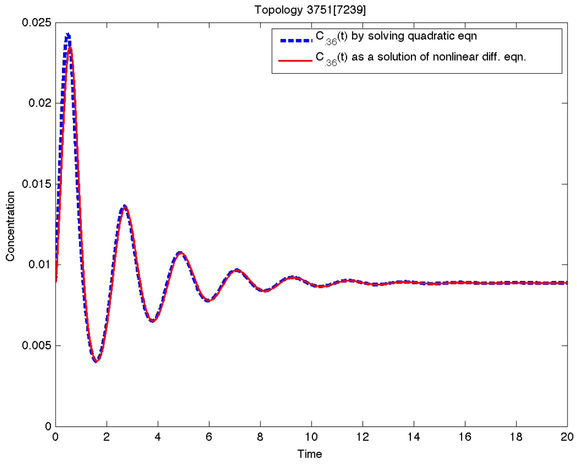

(c) Quadratic approx. and output of nonlinear system

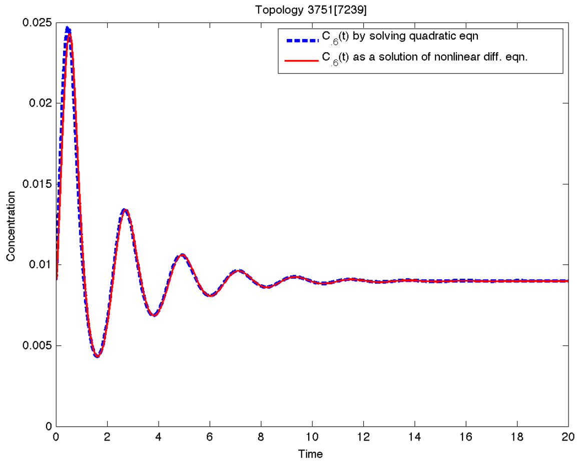

(d) Quadratic approx. and output of nonlinear system

Figure S23. Circuit 22.

Circuit 23.

$$\begin{aligned}
\dot{x}_A &= k_{uA}u\frac{\widetilde{x}_A}{\widetilde{x}_A + K_{uA}} - k_{BA}x_B\frac{x_A}{x_A + K_{BA}} - k_{CA}x_C\frac{x_A}{x_A + K_{CA}} \\
\dot{x}_B &= k_{AB}x_A\frac{\widetilde{x}_B}{\widetilde{x}_B + K_{AB}} - k_{F_BB}x_{F_B}\frac{x_B}{x_B + K_{F_BB}} \\
\dot{x}_C &= k_{AC}x_A\frac{\widetilde{x}_C}{\widetilde{x}_C + K_{AC}} - k_{BC}x_B\frac{x_C}{x_C + K_{BC}} - k_{CC}x_C\frac{x_C}{x_C + K_{CC}}
\end{aligned}$$

Parameters: $K_{uA} = 0.093918$; $k_{uA} = 11.447219$; $K_{BA} = 0.001688$; $k_{BA} = 44.802268$; $K_{CA} = 90.209027$; $k_{CA} = 96.671843$; $K_{AB} = 0.001191$; $k_{AB} = 1.466561$; $K_{F_B} = 9.424319$; $k_{F_B} = 22.745736$; $K_{AC} = 0.113697$; $k_{AC} = 1.211993$; $K_{BC} = 0.009891$; $k_{BC} = 7.239357$; $K_{CC} = 0.189125$; $k_{CC} = 17.910182$

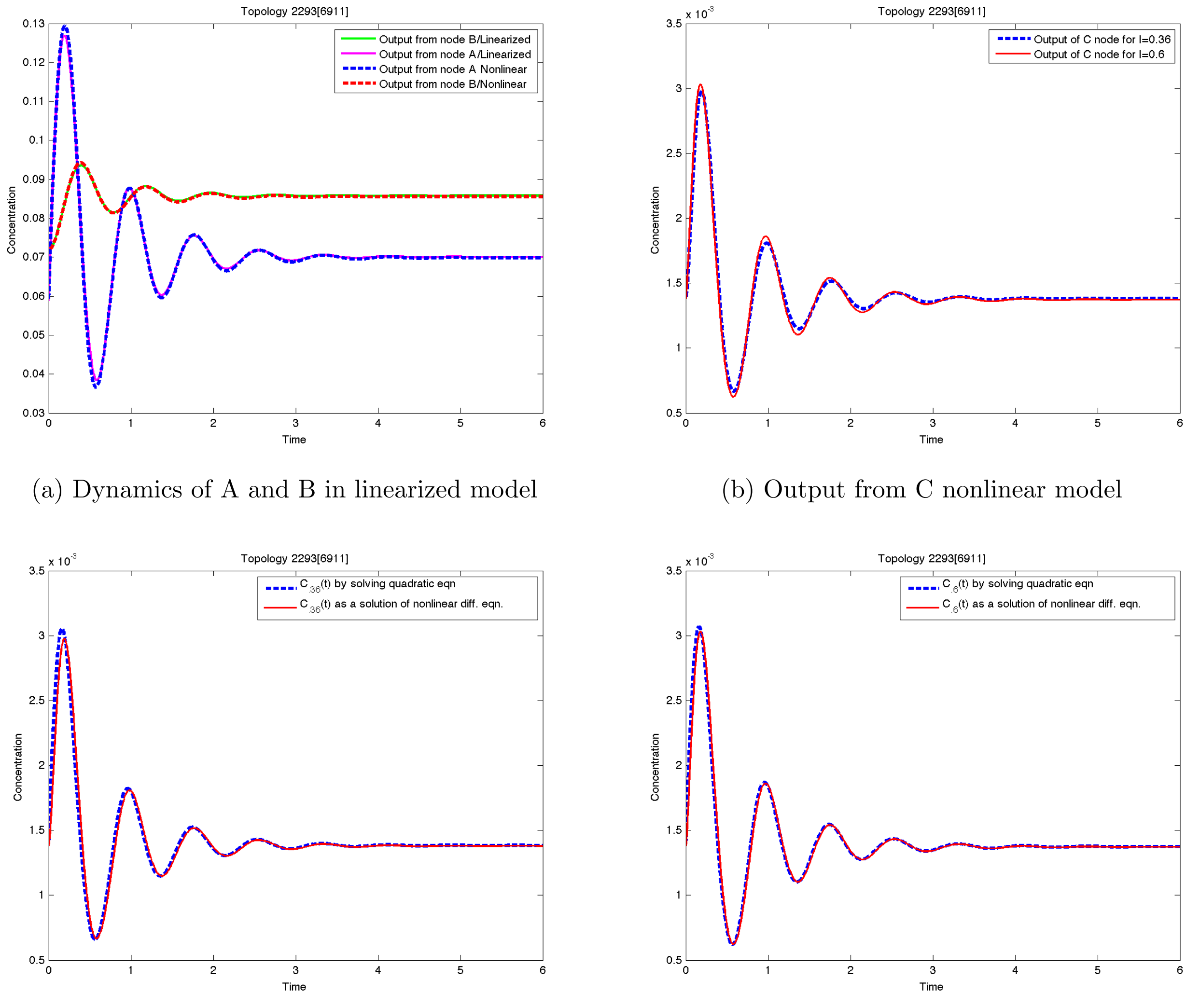

(a) Dynamics of A and B in linearized model

(b) Output from C nonlinear model

(c) Quadratic approx. and output of nonlinear system

(d) Quadratic approx. and output of nonlinear system

Figure S24. Circuit 23.

Circuit 24.

$$
\begin{aligned}
\dot{x}_A &= k_{uA} u \frac{\widetilde{x}_A}{\widetilde{x}_A + K_{uA}} - k_{BA} x_B \frac{x_A}{x_A + K_{BA}} + k_{CA} x_C \frac{\widetilde{x}_A}{\widetilde{x}_A + K_{CA}} \\
\dot{x}_B &= k_{AB} x_A \frac{\widetilde{x}_B}{\widetilde{x}_B + K_{AB}} - k_{F_B B} x_{F_B} \frac{x_B}{x_B + K_{F_B B}} \\
\dot{x}_C &= k_{AC} x_A \frac{\widetilde{x}_C}{\widetilde{x}_C + K_{AC}} - k_{BC} x_B \frac{x_C}{x_C + K_{BC}}
\end{aligned}
$$

Parameters: $K_{uA} = 0.093918$; $k_{uA} = 11.447219$; $K_{BA} = 0.001688$; $k_{BA} = 44.802268$; $K_{CA} = 5.026318$; $k_{CA} = 45.803641$; $K_{AB} = 0.001191$; $k_{AB} = 1.466561$; $K_{F_B} = 9.424319$; $k_{F_B} = 22.745736$; $K_{AC} = 0.113697$; $k_{AC} = 1.211993$; $K_{BC} = 0.009891$; $k_{BC} = 7.239357$

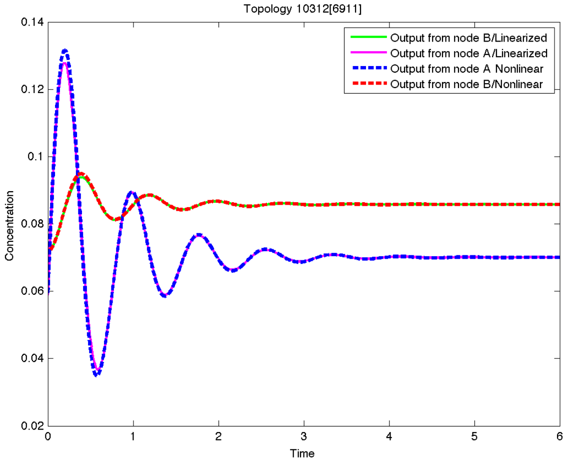

(a) Dynamics of A and B in linearized model

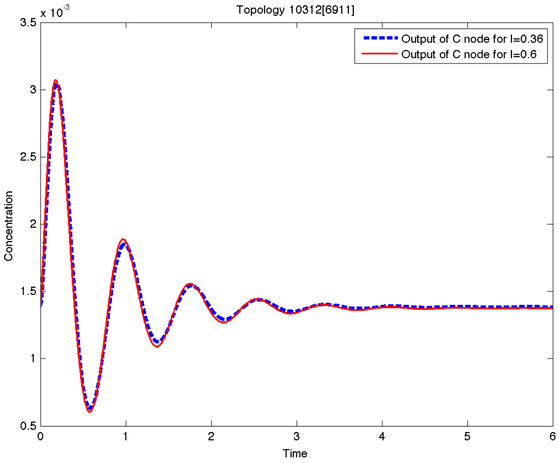

(b) Output from C nonlinear model

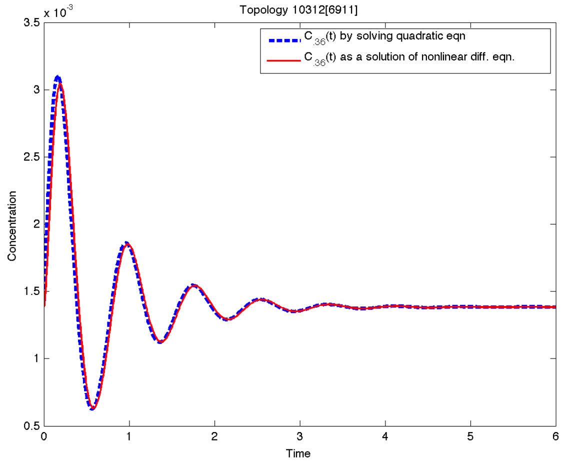

(c) Quadratic approx. and output of nonlinear system

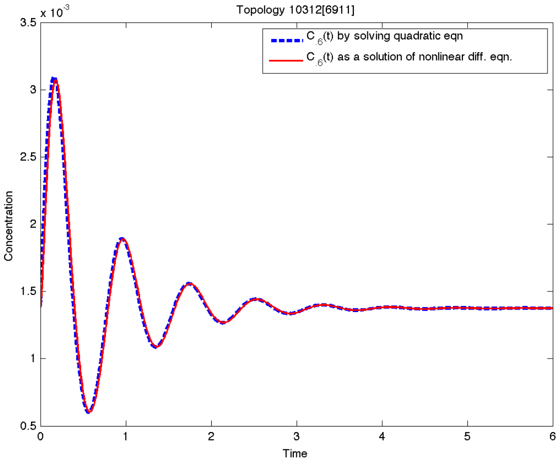

(d) Quadratic approx. and output of nonlinear system

Figure S25. Circuit 24.

Circuit 25.

This is the same topology as in the previous case, only a different parameter set was used:

$K_{AB} = 1.620877$; $k_{AB} = 2.306216$; $K_{F_B} = 2.012565$; $k_{F_B} = 2.700847$; $K_{AC} = 0.010933$; $k_{AC} = 8.968091$; $K_{BA} = 0.001812$; $k_{BA} = 10.039221$; $K_{BC} = 0.014199$; $k_{BC} = 17.762333$; $K_{CA} = 0.002690$; $k_{CA} = 1.506954$; $K_{uA} = 0.161715$; $k_{uA} = 1.93330$

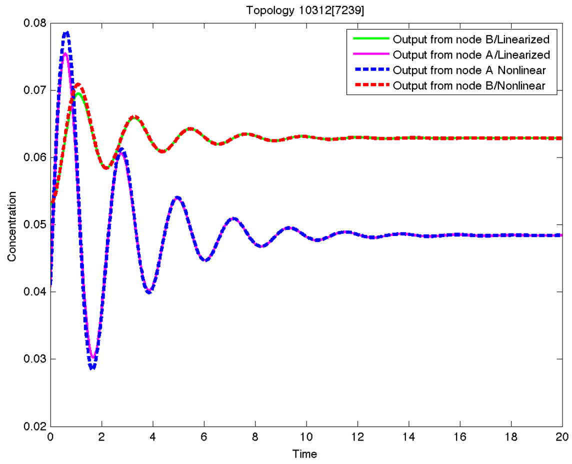

(a) Dynamics of A and B in linearized model

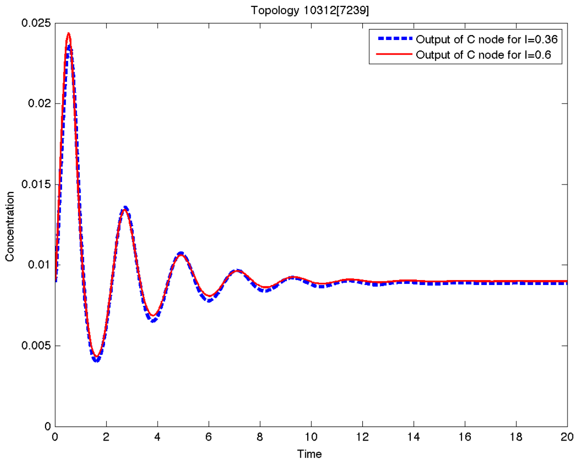

(b) Output from C nonlinear model

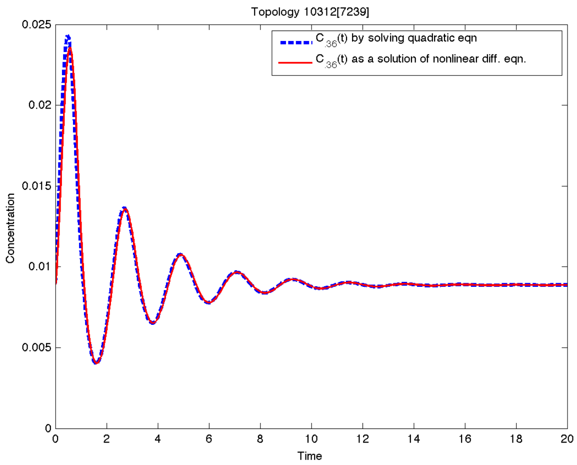

(c) Quadratic approx. and output of nonlinear system

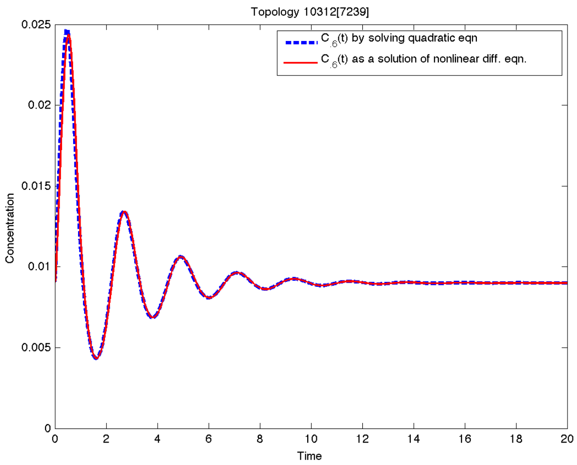

(d) Quadratic approx. and output of nonlinear system

Figure S26. Circuit 25.

2 Ratios $x_A(t)/x_B(t)$

In this section, for each ASI circuit, we show that the ratio $x_A(t)/x_B(t)$ is approximately invariant when inputs are scaled, as discussed in the Main Text.

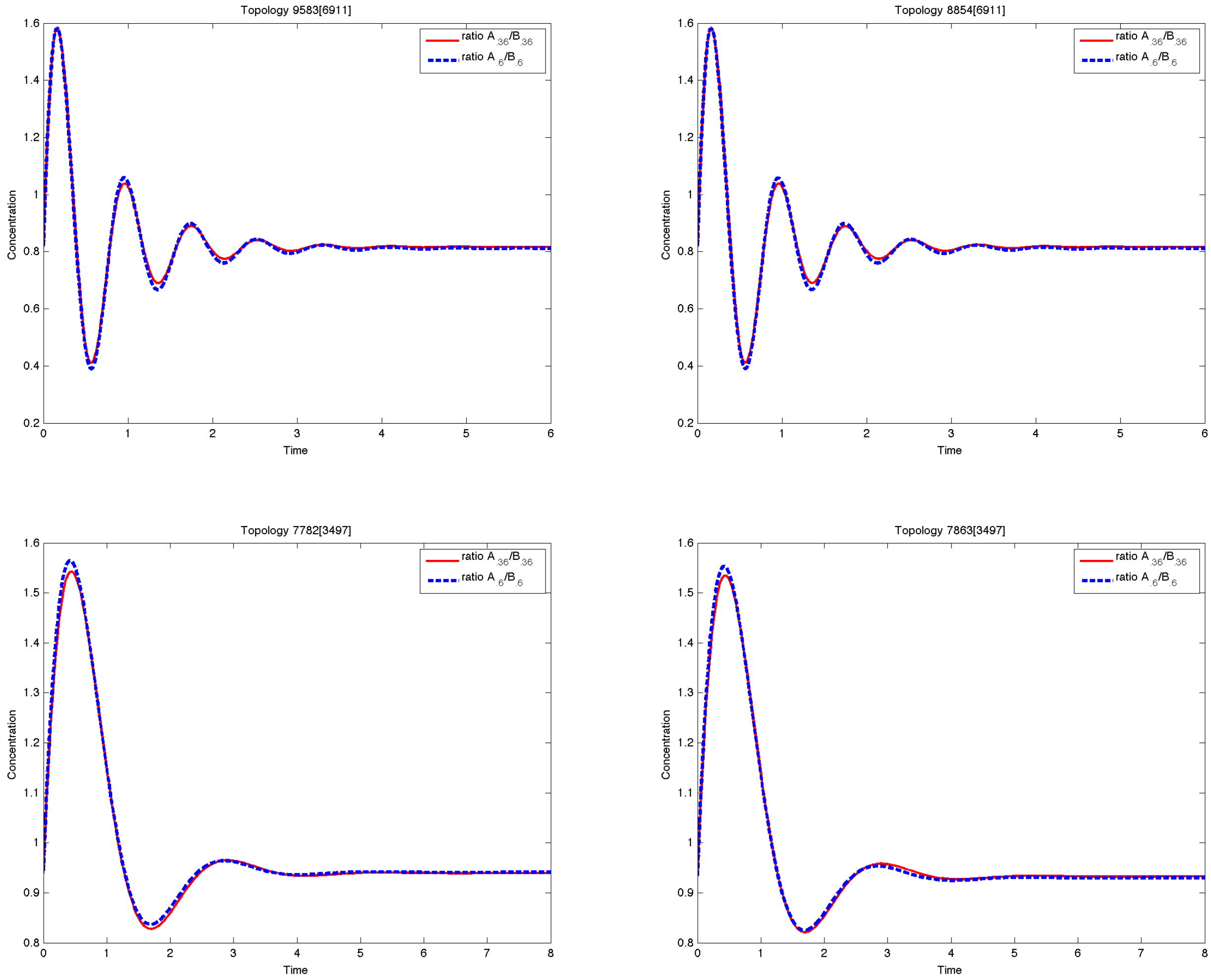

Figure S27. $x_A(t)/x_B(t)$ for Circuits 1-4

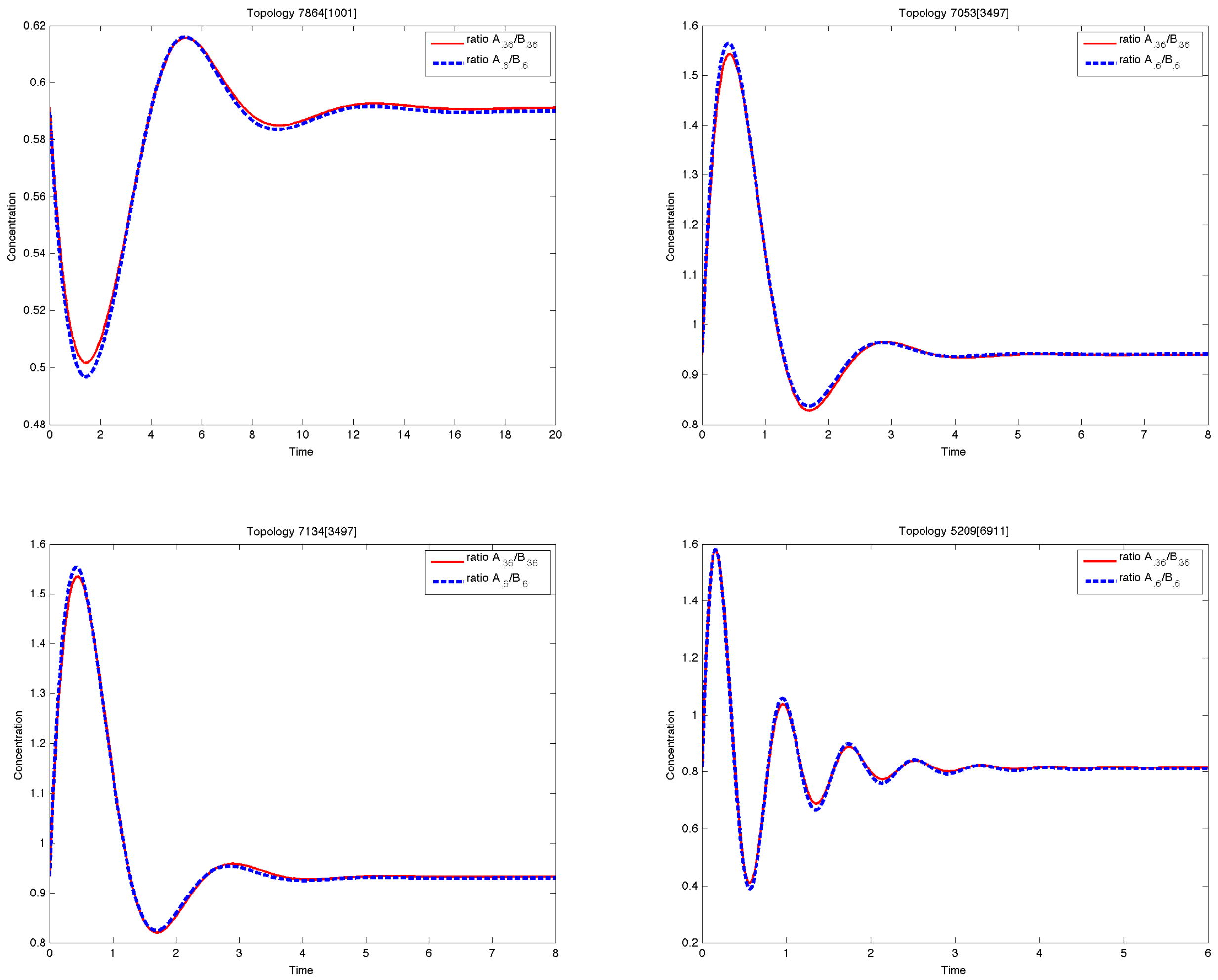

Figure S28. $x_A(t)/x_B(t)$ for Circuits 5-8

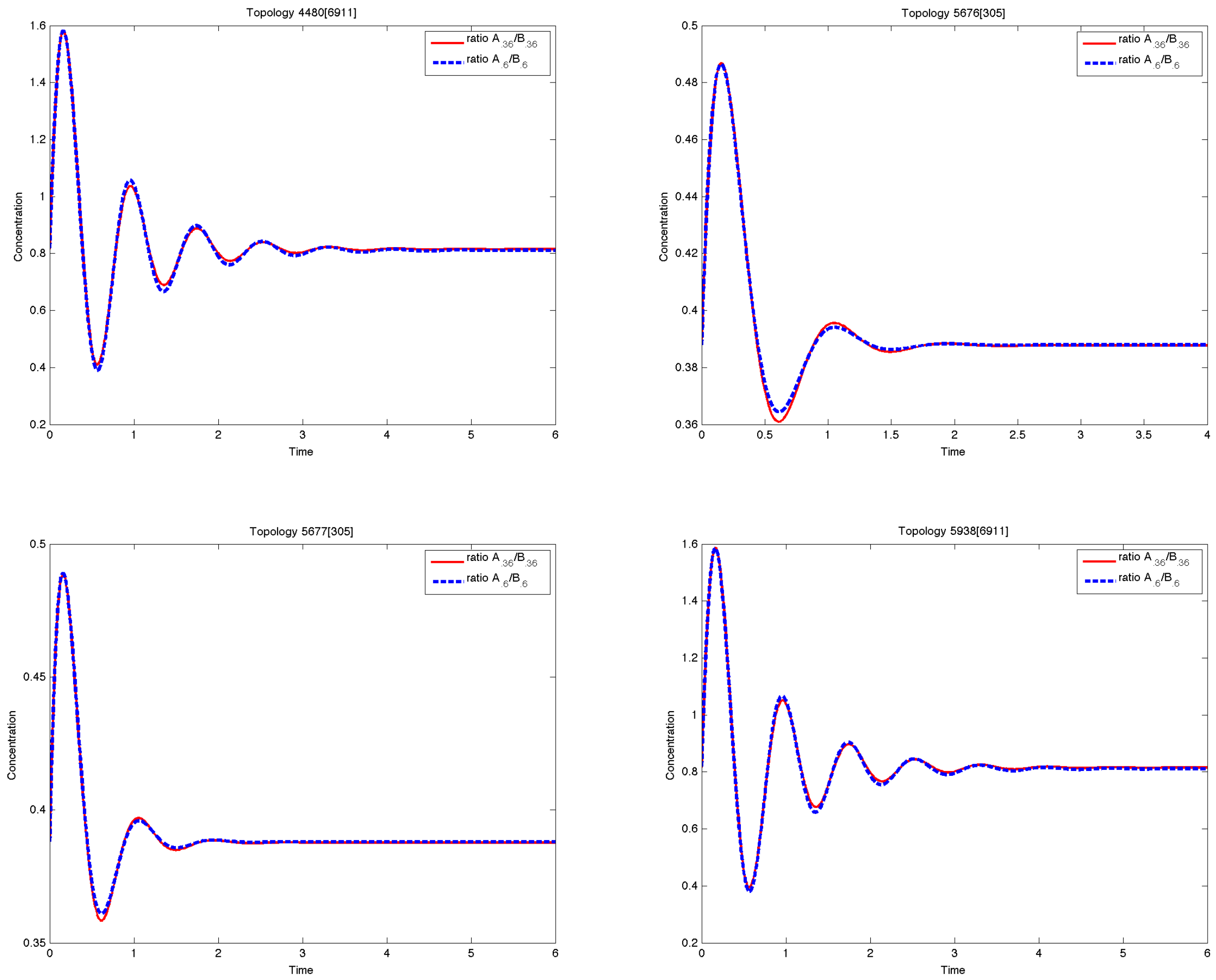

Figure S29. $x_A(t)/x_B(t)$ for Circuits 9-12

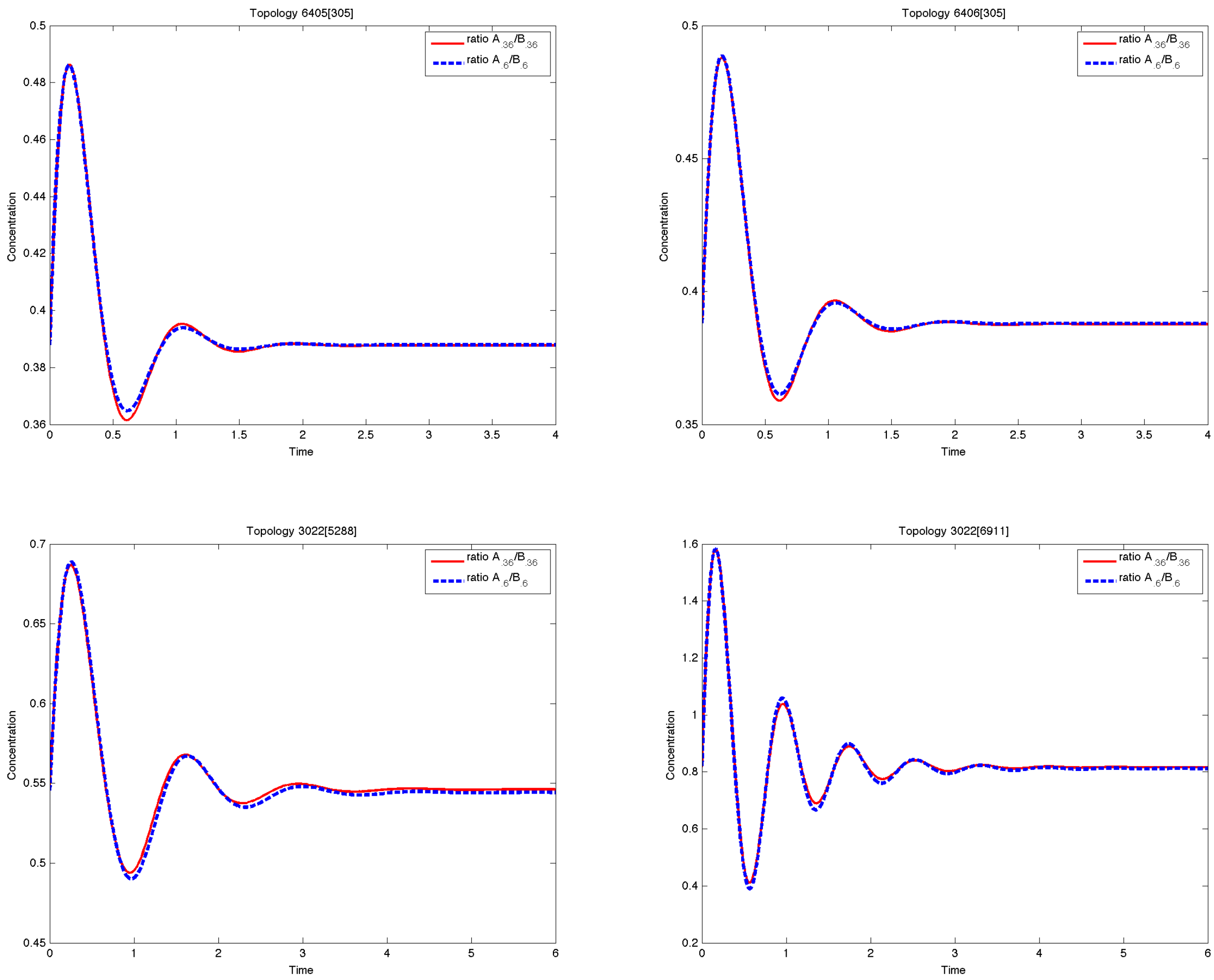

Figure S30. $x_A(t)/x_B(t)$ for Circuits 13-16

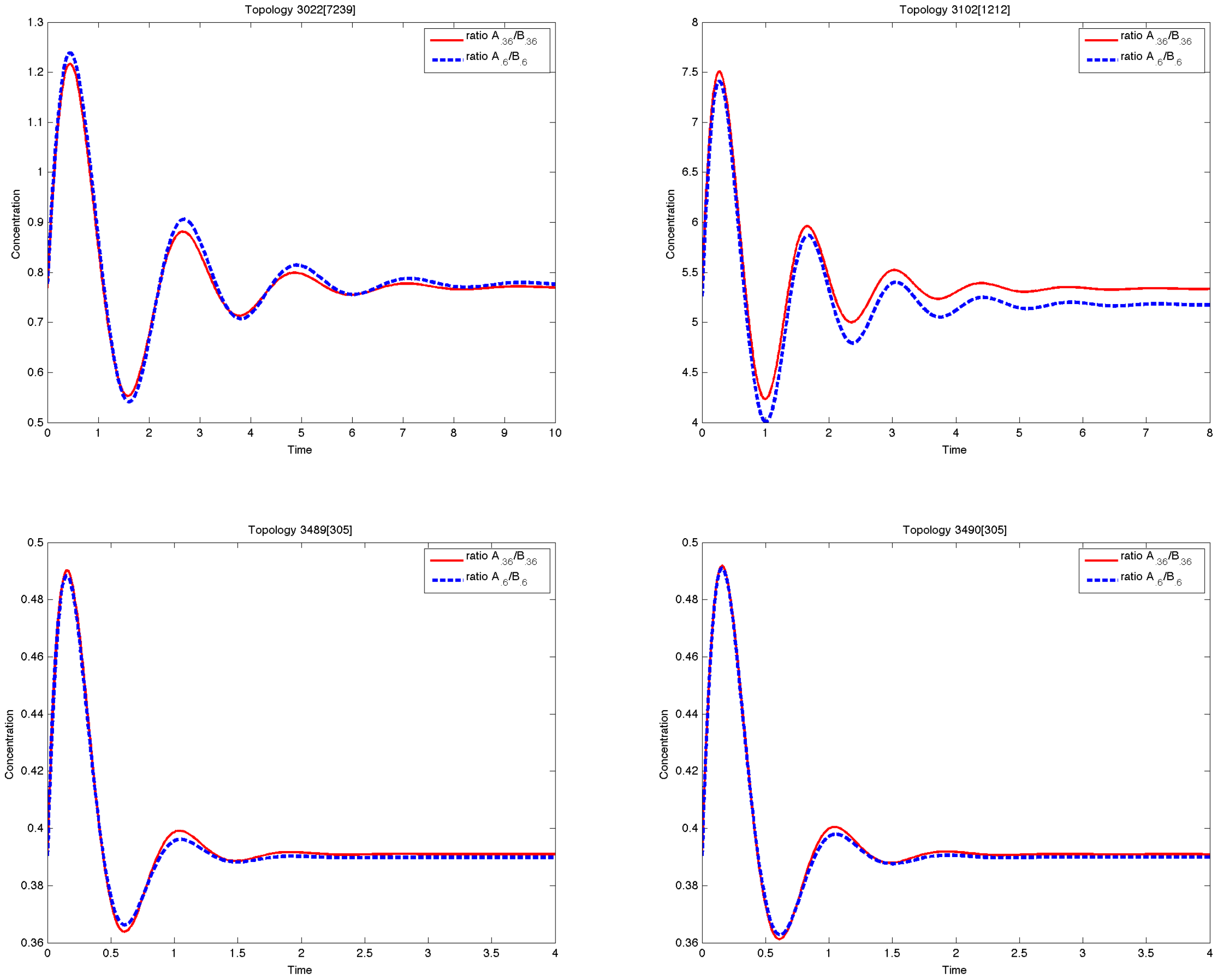

Figure S31. $x_A(t)/x_B(t)$ for Circuits 17-20

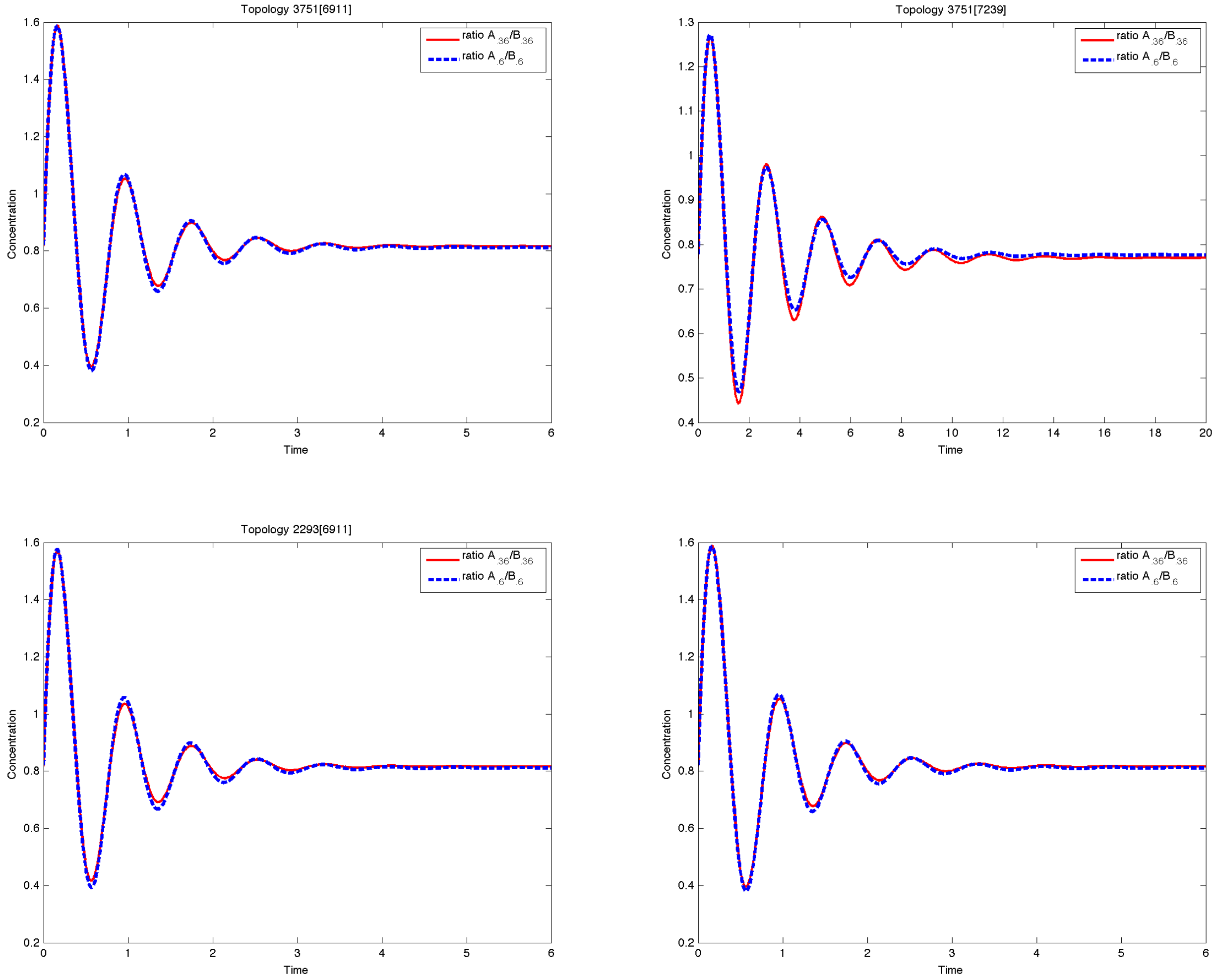

Figure S32. $x_A(t)/x_B(t)$ for Circuits 21-24

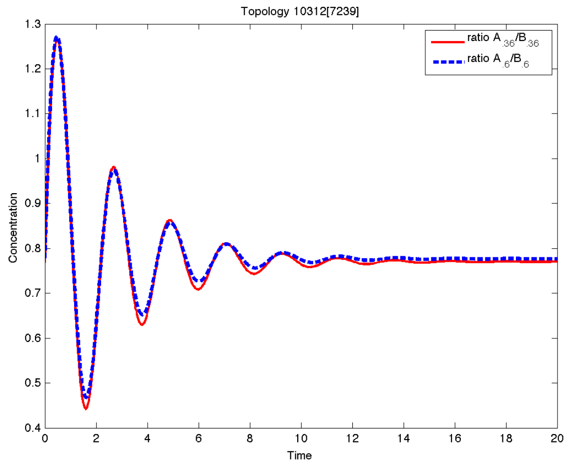

Figure S33. $x_A(t)/x_B(t)$ for Circuit 25.

3 Tables

In this section the following three tables for the 25 identified ASI circuits are shown:

- Table 1. Relative differences in linearization matrices corresponding to different linearizations, $\mathcal{A}_{0.3} = \mathcal{A}(0.3)$, $\mathcal{A}_{0.4} = \mathcal{A}(0.4)$, $\ldots$, $\mathcal{B}_{0.6} = \mathcal{B}(0.6)$, rounded to 3 decimal places. The corresponding expressions are given by:

$$\mathcal{A}_{ij}^{\mathrm{err}} = \sum_{u=0.3,0.4,0.5,0.6} \left| \frac{(\mathcal{A}_u)_{ij} - (\mathcal{A}_{0.45})_{ij}}{(\mathcal{A}_{0.45})_{ij}} \right|$$

  and similarly for $\mathcal{B}^{\mathrm{err}}$. These relative differences are very small. The entries in the table are of the following form: $\mathcal{A}^{\mathrm{err}}$ displayed as $[a_{11}\ a_{12}; a_{21}\ a_{22}]$ and $\mathcal{B}^{\mathrm{err}}$ displayed as $[b_1\ b_2]^T$.

- Table 2. Relative error between original (nonlinear) system with an initial state $\xi = (x_A, x_B)$ corresponding to $u = 0.3$, and applied input $u = 0.36$, and the approximation is $\xi + z(t)$, where z solves the linear system with an initial condition of zero and a constant input of 0.06. Additionally, we provide relative errors between the original (nonlinear) system with an initial state corresponding to $u = 0.5$, and applied input of $u = 0.6$, and the approximation given by $\xi + z(t)$, where z solves the linear system with an initial condition of zero and a constant input of 0.1. The corresponding expressions are given by: $x_{A_{max,u=0.36}}^{err} = \max_{t\geq 0} \left| \frac{x_{A_{0.36}^{\mathrm{L}}}(t) - x_{A_{0.36}^{\mathrm{N}}}(t)}{x_{A_{0.36}^{\mathrm{N}}}(t)} \right|$, $x_{A_{max,u=0.6}}^{err} = \max_{t\geq 0} \left| \frac{x_{A_{0.6}^{\mathrm{L}}}(t) - x_{A_{0.6}^{\mathrm{N}}}(t)}{x_{A_{0.6}^{\mathrm{N}}}(t)} \right|$,
  where N denotes the nonlinear system, and L denotes the linear system.
  We define similarly for $x_{B_{max,u=0.36}}^{err}$ and $x_{B_{max,u=0.6}}^{err}$.

- Table 3. Homogeneity property of the states x_A and x_B. For a constant input u, it holds that $\sigma(pu) \approx p\sigma(u)$, where $\sigma(u)$ is a unique steady state (x_A, x_B).

**Circuit**	$\mathcal{A}^{\mathbf{err}}$	$\mathcal{B}^{\mathbf{err}}$
1	$[0.069\ 0.004; 0\ 0.005]$	$[0.002\ 0]^T$
2	$[0.084\ 0.006; 0.019\ 0.015]$	$[0.004\ 0]^T$
3	$[0.069\ 0.004; 0\ 0.005]$	$[0.002\ 0]^T$
4	$[0.114\ 0.007; 0.011\ 0.003]$	$[0.002\ 0]^T$
5	$[0.045\ 0.003; 0.01\ 0.033]$	$[0\ 0]^T$
6	$[0.075\ 0.012; 0.021\ 0.012]$	$[0.015\ 0]^T$
7	$[0.057\ 0.012; 0.021\ 0.012]$	$[0.012\ 0]^T$
8	$[0.055\ 0.012; 0.019\ 0.009]$	$[0.016\ 0]^T$
9	$[0.069\ 0.004; 0\ 0.005]$	$[0.002\ 0]^T$
10	$[0.037\ 0.022; 0.009\ 0.0707]$	$[0.002\ 0]^T$
11	$[0.037\ 0.022; 0.007\ 0.009]$	$[0.002\ 0]^T$
12	$[0.025\ 0.029; 0.007\ 0.006]$	$[0.012\ 0]^T$
13	$[0.037\ 0.022; 0.009\ 0.007]$	$[0.002\ 0]^T$
14	$[0.036\ 0.022; 0.007\ 0.009]$	$[0.002\ 0]^T$
15	$[0.07\ 0.004; 0\ 0.005]$	$[0.002\ 0]^T$
16	$[0.07\ 0.004; 0\ 0.005]$	$[0.002\ 0]^T$
17	$[0.073\ 0.012; 0.017\ 0.009]$	$[0.015\ 0]^T$
18	$[0.051\ 0.004; 0\ 0.005]$	$[0.002\ 0]^T$
19	$[0.066\ 0.013; 0.018\ 0.009]$	$[0.015\ 0]^T$
20	$[0.048\ 0.013; 0.018\ 0.009]$	$[0.016\ 0]^T$
21	$[0.051\ 0.004; 0\ 0.005]$	$[0.002\ 0]^T$
22	$[0.233\ 0; 0.011\ 0.003]$	$[0.002\ 0]^T$
23	$[0.069\ 0.004; 0\ 0.005]$	$[0.002\ 0]^T$
24	$[0.051\ 0.004; 0\ 0.005]$	$[0.002\ 0]^T$
25	$[0.233\ 0; 0.011\ 0.003]$	$[0.002\ 0]^T$

Table S1. Relative error in linearization matrices

**Circuit**	$x_{A_{max,u=0.36}}^{err}$	$x_{B_{max,u=0.36}}^{err}$	$x_{A_{max,u=0.6}}^{err}$	$x_{B_{max,u=0.6}}^{err}$
1	0.055	0.011	0.028	0.005
2	0.008	0.007	0	0.002
3	0.055	0.010	0.028	0.005
4	0.03	0.007	0.012	0.004
5	0.031	0.006	0.003	0
6	0.015	0.016	0.011	0.005
7	0.023	0.021	0.005	0.004
8	0.023	0.021	0.004	0.004
9	0.055	0.01	0.028	0.005
10	0.097	0.020	0.081	0.016
11	0.010	0.020	0.084	0.016
12	0.033	0.021	0.024	0.010
13	0.097	0.020	0.081	0.016
14	0.010	0.02	0.084	0.016
15	0.056	0.010	0.028	0.005
16	0.056	0.010	0.028	0.005
17	0.027	0.022	0.004	0.004
18	0.047	0.010	0.028	0.006
19	0.027	0.023	0.005	0.004
20	0.023	0.021	0.005	0.004
21	0.04	0.009	0.034	0.004
22	0.116	0.027	0.05	0.013
23	0.055	0.010	0.028	0.005
24	0.045	0.01	0.027	0.005
25	0.117	0.03	0.05	0.013

Table S2. Relative error between nonlinear and linearized system

**Circuit**	$\sigma(u_{0.3})/0.3$	$\sigma(u_{0.4})/0.4$	$\sigma(u_{0.5})/0.5$	$\sigma(u_{0.6})/0.6$
1	$(0.195, 0.239)$	$(0.193, 0.237)$	$(0.192, 0.236)$	$(0.19, 0.234)$
2	$(0.199, 0.364)$	$(0.197, 0.359)$	$(0.194, 0.356)$	$(0.192, 0.353)$
3	$(0.195, 0.239)$	$(0.193, 0.237)$	$(0.192, 0.236)$	$(0.191, 0.234)$
4	$(0.132, 0.172)$	$(0.131, 0.170)$	$(0.131, 0.169)$	$(0.13, 0.168)$
5	$(0.591, 0.11)$	$(0.58, 0.109)$	$(0.57, 0.109)$	$(0.561, 0.108)$
6	$(0.206, 0.526)$	$(0.198, 0.507)$	$(0.192, 0.493)$	$(0.188, 0.481)$
7	$(0.208, 0.529)$	$(0.2, 0.512)$	$(0.194, 0.498)$	$(0.19, 0.486)$
8	$(0.206, 0.530)$	$(0.199, 0.512)$	$(0.193, 0.499)$	$(0.189, 0.486)$
9	$(0.195, 0.239)$	$(0.194, 0.237)$	$(0.192, 0.236)$	$(0.190, 0.234)$
10	$(0.078, 0.083)$	$(0.075, 0.08)$	$(0.073, 0.078)$	$(0.071, 0.076)$
11	$(0.077, 0.083)$	$(0.074, 0.08)$	$(0.072, 0.078)$	$(0.071, 0.076)$
12	$(0.153, 0.09)$	$(0.145, 0.086)$	$(0.139, 0.082)$	$(0.135, 0.08)$
13	$(0.078, 0.083)$	$(0.075, 0.08)$	$(0.073, 0.078)$	$(0.071, 0.076)$
14	$(0.077, 0.083)$	$(0.074, 0.08)$	$(0.072, 0.078)$	$(0.071, 0.076)$
15	$(0.195, 0.239)$	$(0.193, 0.237)$	$(0.191, 0.235)$	$(0.190, 0.234)$
16	$(0.195, 0.239)$	$(0.193, 0.237)$	$(0.191, 0.236)$	$(0.19, 0.234)$
17	$(0.204, 0.526)$	$(0.197, 0.508)$	$(0.191, 0.494)$	$(0.186, 0.48)$
18	$(0.196, 0.24)$	$(0.193, 0.238)$	$(0.192, 0.236)$	$(0.19, 0.235)$
19	$(0.205, 0.528)$	$(0.197, 0.509)$	$(0.192, 0.494)$	$(0.187, 0.481)$
20	$(0.206, 0.532)$	$(0.199, 0.513)$	$(0.193, 0.5)$	$(0.189, 0.487)$
21	$(0.196, 0.24)$	$(0.194, 0.237)$	$(0.192, 0.236)$	$(0.191, 0.235)$
22	$(0.136, 0.177)$	$(0.134, 0.173)$	$(0.133, 0.171)$	$(0.132, 0.17)$
23	$(0.195, 0.239)$	$(0.193, 0.237)$	$(0.192, 0.236)$	$(0.191, 0.234)$
24	$(0.196, 0.240)$	$(0.194, 0.237)$	$(0.192, 0.236)$	$(0.190, 0.235)$
25	$(0.136, 0.178)$	$(0.134, 0.173)$	$(0.133, 0.171)$	$(0.132, 0.17)$

Table S3. $\sigma(u)/u$ for constant inputs $u = 0.3, 0.4, 0.5, 0.6$
